# Chalcogen‐Transfer Rearrangement: Exploring Inter‐ versus Intramolecular P−P Bond Activation

**DOI:** 10.1002/chem.202002481

**Published:** 2020-11-09

**Authors:** Roman Franz, Sina Nasemann, Clemens Bruhn, Zsolt Kelemen, Rudolf Pietschnig

**Affiliations:** ^1^ Institute for Chemistry and CINSaT University of Kassel Heinrich Plett-Strasse 40 34132 Kassel Germany; ^2^ Department of Inorganic and Analytical Chemistry Budapest University of Technology and Economics and MTA-BME, Computation Driven Chemistry Research Group Szent Gellért tér 4 1111 Budapest Hungary

**Keywords:** bond activation, chalcogens, ferrocenes, multinuclear NMR spectroscopy, phosphorus

## Abstract

*tert*‐Butyl‐substituted diphospha[2]ferrocenophane has been used as a stereochemically confined diphosphane to explore the addition of O, S, Se and Te. Although the diphosphanylchalcogane has been obtained for tellurium, all other chalcogens give diphosphane monochalcogenides. The latter transform via chalcogen‐transfer rearrangement to the corresponding diphosphanylchalcoganes upon heating. The kinetics of this rearrangement has been followed with NMR spectroscopy supported by DFT calculations. Intermediates during rearrangement point to a disproportionation/synproportionation mechanism for the S and Se derivatives. Cyclic voltammetry together with DFT studies indicate ferrocene‐centred oxidation for most of the compounds presented.

## Graphical abstract

**Chalcogen transfer**: Investigation of the addition of O, S, Se and Te to a model diphosphane and subsequent thermal rearrangement point to a disproportionation/synproportionation mechanism (see scheme).

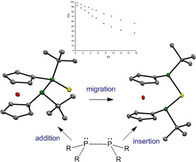

## Introduction

**Scheme 1:**
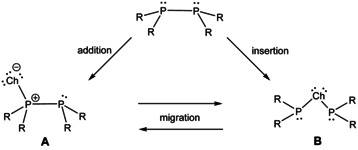
Illustration of addition, insertion and migration of chalcogen atoms for the formation and interconversion of isomeric diphosphane monochalcogenides **A** and diphosphanylchalcoganes **B**.

Chalcogen transfer is a fundamental reaction in molecular chemistry and chalcogenophosphoranes (phosphine chalcogenides) are well established reagents for this purpose.^[1]^ Although the preparation of phosphorus sulfides, as an example, is known since centuries, mechanistic details on how chalcogen atoms are transferred are still quite limited with some light shed by a study addressing the oxidation of white phosphorus with sulfur in the melt and at low temperature under thermal and photochemical conditions.^[2]^ The scenario is complicated by initial chalcogen addition to either a phosphorus lone pair, or insertion into a P‐P bond, potentially followed by subsequent interconversion via chalcogen migration proceeding intra‐ or intermolecularly (Scheme 1). Diphosphanes and diphosphane monochalcogenides are suitable model compounds for the study of such processes featuring a phosphorus lone pair as well as a P‐P bond. For the case of sulfur, diphosphane monosulfides have been obtained via desulfurization of diphosphane disulfides,^[3]^ oxidation of diphosphanes with elemental chalcogen,[^4^, ^5^] or synproportionation of diphosphanes and diphosphane disulfides.^[4]^ For the heavier chalcogen selenium, the corresponding diphosphane monoselenide was reported using the oxidation route,^[6]^ and via metathesis of metal selenide and halophosphanes.[^7^, ^8^] In a comprehensive study,^[8]^ the steric influence on the relative ratio of the isomeric diphosphane monochalcogenides **A** and diphosphanylchalcoganes **B** has been explored for Ch=S, Se and Te, including the thermal conversion **A→B** which had been studied independently for Ch=O before.^[9]^ The aspect of ring‐strain on this transformation has been demonstrated elegantly for an *o*‐carborane bridged diphosphane with Ch=Se.^[10]^ While diphosphanylchalcogane isomer **B** is observed exclusively for Ch=Te,^[8]^ the opposite is true for oxygen, where diphosphane monochalcogenide **A** is dominant, except for stereo‐electronically unique organofluoro substituents.^[11]^

Based on previous results,^[12]^ we considered *tert‐*butyl substituted diphospha[2]ferrocenophane **1** as suitable diphosphane to study chalcogen‐transfer reactions as it combines two stereogenic P^III^ centres with lone pairs and a non‐polar >P‐P< bond. This compound is available as a single diastereomer with *trans* orientation of the *tert‐*butyl groups adjacent to the P‐stereogenic centres,^[13]^ which should allow facile monitoring of epimerization via ^31^P NMR spectroscopy. Here we report our investigation concerning addition vs. insertion of chalcogen atoms to the diphosphane unit in **1** and its stereochemical course.

## Results and Discussion

**Scheme 2:**
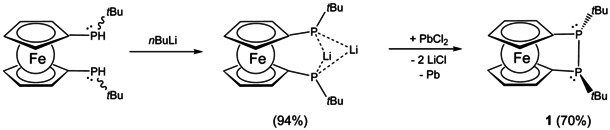
Oxidative formation of **1** with lead(II) chloride by lithiation of Fe(C_5_H_4_P*t*BuH)_2_.

As a starting point of our investigation an improved synthesis of diphospha[2]ferrocenophane **1** has been developed furnishing analytically pure **1** by simple recrystallization in good yields (70 %). Our approach employs oxidative P‐P bond formation from the corresponding bisphosphanide using various oxidants (SiBr_4_, CCl_4_, CHCl_3_, CHBr_3_, PbCl_2_) out of which PbCl_2_ gave best results (Scheme 2). The advantage over a published route via reductive P‐P bond formation accompanied by formation of by‐products, is the ease of purification requiring no column chromatography as in the previous method.^[13]^

**Scheme 3:**
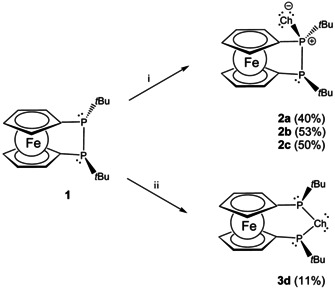
Monooxidation of **1** to **2 a**–**c** and **3 d** (Ch=O (**a**), S (**b**), Se (**c**), Te (**d**); i: 1 equiv H_2_O_2_, rt, 30 min (**2 a**); 1/8 S_8_, rt, overnight (**2 b**); 1 equiv. Se (grey), rt, overnight (**2 c**). ii: 1 equiv Te, 120 °C, overnight (**3 d**)).

To explore whether one chalcogen atom (Ch=O, S, Se, Te) can be transferred selectively to diphosphane **1** by stoichiometric control, we employed reagents containing the respective chalcogen–chalcogen bonds. While H_2_O_2_ or *tert‐*butyl hydroperoxide, S_8_ and grey selenium furnish cleanly monooxidized diphosphane monochalcogenides **2 a**–**c** at room temperature, no reaction is observed with elemental tellurium under these conditions (Scheme 3). Nevertheless, at elevated temperature partial oxidation of **1** by tellurium can be observed (33 % yield besides unreacted **1**). Unlike for the other chalcogens, oxidation of **1** with tellurium leads exclusively to the ring expanded telluradiphospha[3]ferrocenophane **3 d**.

**Figure 1:**
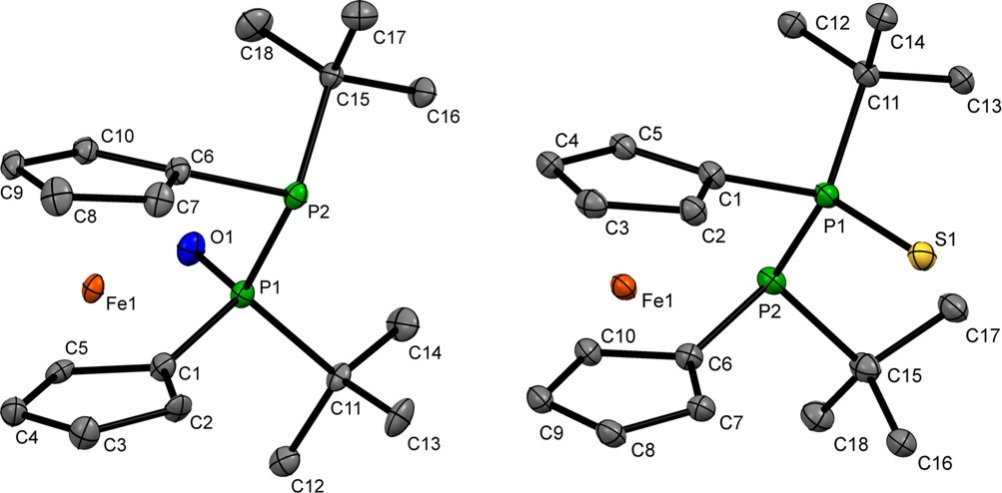
Molecular structure of **2 a** (left) and **2 b** (right) in the solid state. Ellipsoids were shown at 30 % (**2 a**) or 10 % (**2 b**) probability. Selected bond lengths and angles of **2 a**: twist angle *δ*=19.5(6)°, ΣP2=309.3(9)°; bond lengths: P1−P2 2.240(3) Å, P1−O1 1.487(6) Å, P1−C_*t*Bu_ 1.858(8) Å, P2−C_*t*Bu_ 1.884(9) Å; **2 b**: twist angle *δ*=20.3(6)°, ΣP2=311.0(8)°; bond lengths: P1−P2 2.246(2) Å, P1−S1 1.965(2) Å, P1−C_*t*Bu_ 1.863(7) Å, P2−C_*t*Bu_ 1.881(8) Å.

Upon monooxidation, the ^31^P NMR chemical shift of 20.6 ppm in **1**,^[13]^ changes to lower field for the tetracoordinate phosphorus atom in **2**, while its trivalent counterpart is shifted to higher field (Table 1). Chemical inequivalence of the ^31^P nuclei in **2** entails signal splitting, for which the value of ^1^ *J*_PP_ increases with the size of the chalcogen (Table 1). For **2 c** the ^77^Se chemical shift at −311.7 ppm shows coupling to the directly bonded tetracoordinate and the trivalent phosphorus atoms (^1^ *J*_SeP_=725 Hz, ^2^ *J*_SeP_=30 Hz). Identity and purity of the above mentioned monooxidized diphosphanes was further corroborated by ^1^H, ^13^C NMR, mass spectrometry and elemental analysis. Single crystals suitable for X‐ray diffraction have been obtained for **2 a**,**b** (Figure 1). Remarkably the P‐P bond lengths, 2.240(3) Å in **2 a** and 2.246(2) Å in **2 b** are almost identical to the one in non‐oxidized **1**.^[13a]^ Compared with the latter, the tetracoordinate phosphorus atoms in **2 a** and **2 b** increase to adapt to the quasi‐tetrahedral coordination geometry. The tilt angle α slightly decreases in the series **1** (13.6(1)°),^[13a]^ **2 a** (11.9(4)°) and **2 b** (12.8(3)°) indicating further release of angular strain upon oxidation.

**Table 1:** ^31^P NMR chemical shifts and coupling constants of **2 a**–**c**.

	δ(^31^P) [ppm]		^1^ *J*_PP_ [Hz]
	>P−	>P<	
**2 a**	7.3	79.6	272
**2 b**	15.6	86.2	301
**2 c**	18.3	71.3	309

**Scheme 4:**
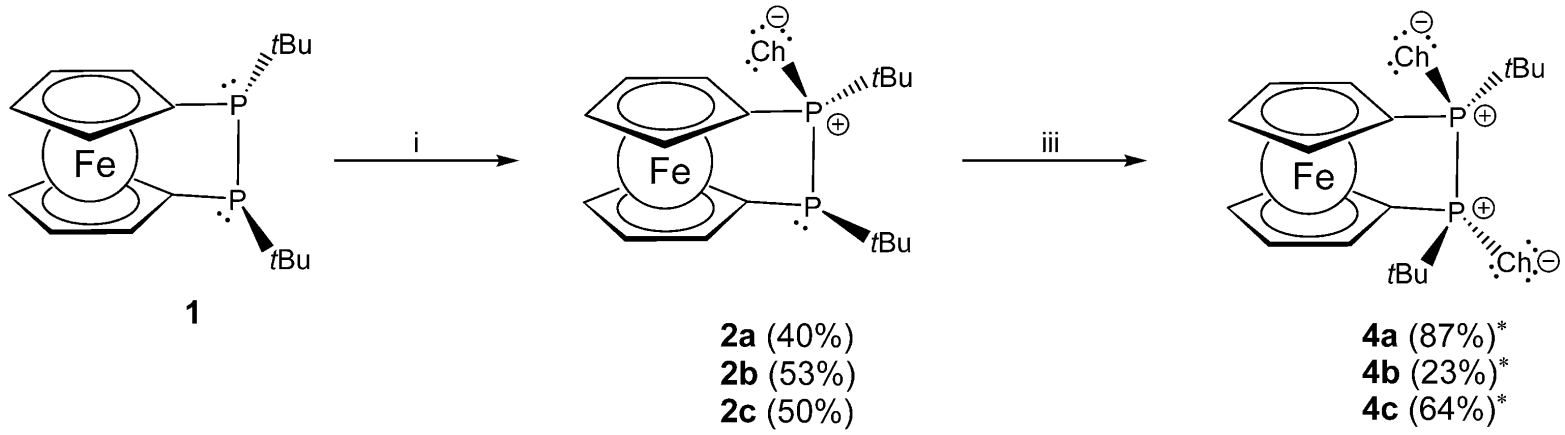
Formation of twofold oxidized **4** via oxidation of **2** (Ch=O (**a**), S (**b**), Se (**c**); i: 1 equiv H_2_O_2_, rt, 30 min (**2 a**); 1/8 S_8_, rt, overnight (**2 b**); 1 equiv Se (grey), rt, overnight (**2 c**); iii: 1 equiv H_2_O_2_, rt, 30 min (**4 a**); 1/8 S_8_, 110 °C, overnight (**4 b**); 1 equiv Se (grey), rt, 6 h (**4 c**)). [*] Yields refer to the direct conversion of **1** with 2 equiv of the chalcogen source.

With an excess of the oxidation reagents mentioned above, twofold oxidation of **1** with chalcogens can be achieved as well. The resulting product is identical regardless whether **1** or **2** are exposed to excess oxidant, which indicates a stepwise oxidation of **1** via **2** to **4** (Scheme 4). The transformation can be followed easily by ^31^P NMR spectroscopy and product **4** shows a single resonance indicating two chemically equivalent phosphorus nuclei in **4**. These resonances in the ^31^P NMR spectra at 68.3 ppm (**4 a**), 81.8 ppm (**4 b**) and 65.6 ppm **(4 c)** are deshielded with respect to **1** but appear at slightly higher field than the respective tetracoordinate phosphorus nuclei in **2**. The resulting selenophosphorane **4 c** shows a resonance at −275.6 ppm in the ^77^Se NMR spectra showing couplings of ^1^ *J*_SeP_=741 Hz and ^2^ *J*_SeP_=17 Hz which is comparable to the values obtained for **2 c**.

**Figure 2:**
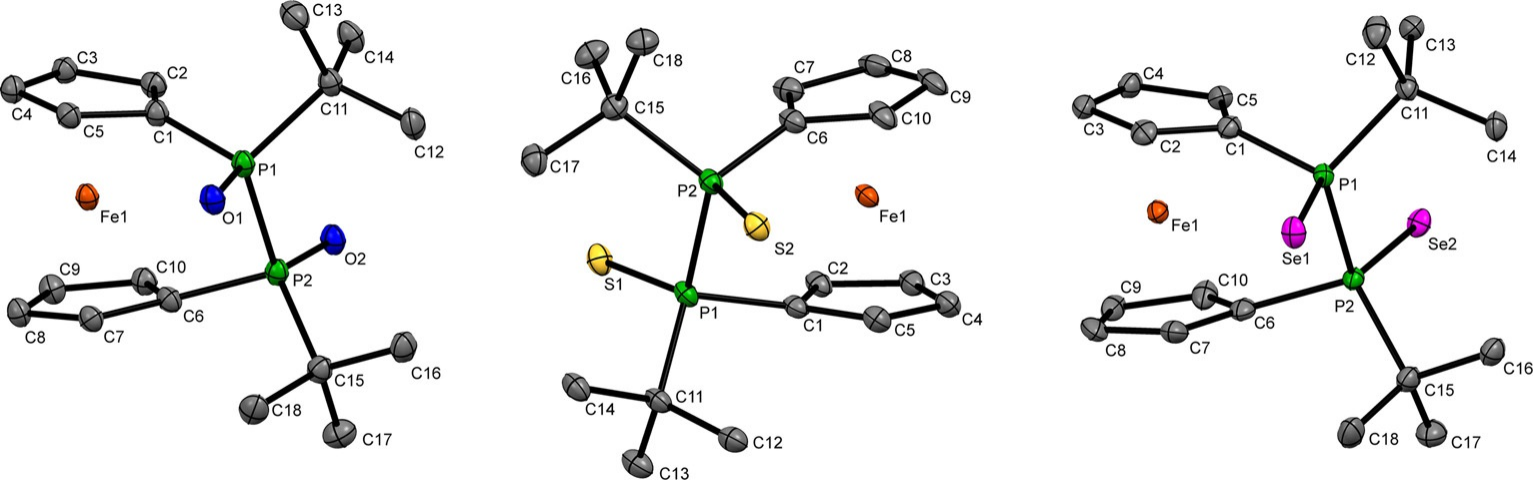
Molecular structure of **4 a** (left), **4 b** (middle) and **4 c** (right) in the solid state. For **4 c** two molecules were found within the unit cell, from which one is depicted. The phosphorus and selenium atoms in **4 c** show a small disorder (ca. 8–16 %) over two positions, which is not shown in the Figure. Ellipsoids were shown at 30 % probability. Important bond lengths and angles of **4 a**: twist angle *δ*=14.6(2)°; bond lengths: P1−P2 2.2516(9) Å, P1−O1 1.4895(16) Å, P1−C_*t*Bu_ 1.838(2) Å; **4 b**: twist angle *δ*=16.7(2)°; bond lengths: P1−P2 2.2695(9) Å, P1−S1 1.9597(10) Å, P1−C_*t*Bu_ 1.872(3) Å; **4 c**: twist angle *δ*=19.6(4)°; bond lengths: P1−P2 2.2793(16) Å, P1−Se1 2.1066(13) Å, P1−C_*t*Bu_ 1.866(5) Å.

The identity of **4 a**–**c** has been further corroborated by single crystal X‐ray diffraction (Figure 2). In the series **4 a**–**c**, the *tert‐*butyl groups are oriented to opposite sides of the [2]ferrocenophane ring adopting a *trans* configuration. Remarkably, the P‐P bond lengths 2.2516(9) Å in **4 a**, 2.2695(9) Å in **4 b** and 2.2793(16) Å in **4 c** are almost identical to the one in non‐oxidized **1**.^[13a]^ The tilt angle *α*=11.0(1)° **(4 a)**, 10.6(1)° **(4 b)**, 12.0(2)° **(4 c)** is slightly lower than in **1** and **2** (v.s.).

### Rearrangement

**Figure 3:**
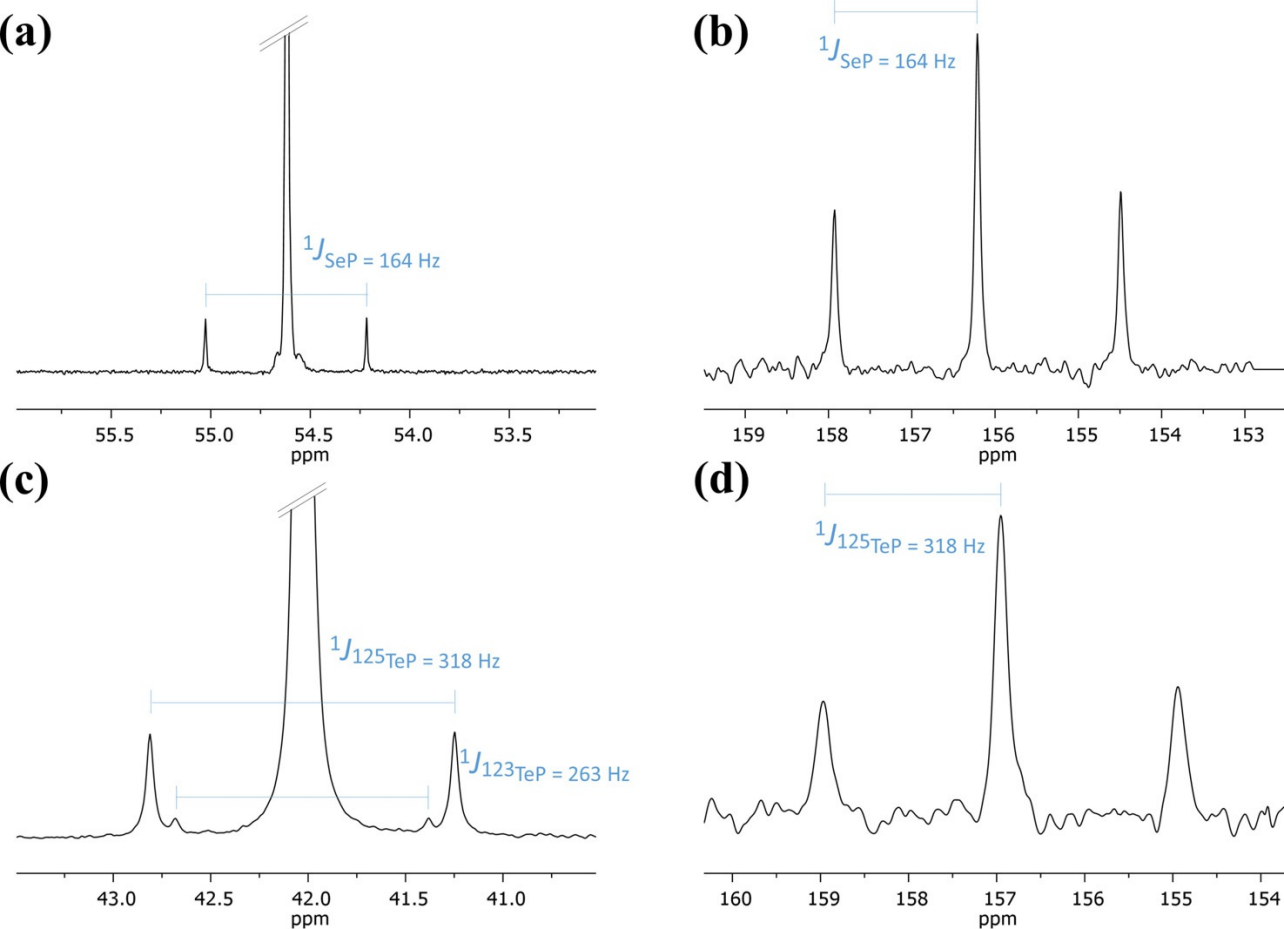
^31^P{^1^H} NMR spectra of **3 c** (a) and **3 d** (c); ^77^Se NMR spectrum of **3 c** (b) and ^125^Te NMR spectrum of **3 d** (d; C_6_D_6_, 300 K).

An interesting feature is observed for diphosphane monochalcogenides **2 b**,**c** which transform slowly into the respective diphosphanylchalcoganes **3 b**,**c** upon heating. While this migratory insertion requires boiling mesitylene over two weeks in the case of sulfur compound **2 b**, its selenium analogue **2 c** rearranges more facile, at 120 °C within few hours. Compounds **3 b**,**c** obtained by rearrangement of **2 b**,**c**, as well as **3 d**, directly obtained by oxidation of **1** (v.s.), are formed in a stereospecific manner showing a single resonance in the ^31^P{^1^H} NMR spectra at 51.8 ppm **(3 b)**, 54.6 ppm **(3 c)** and 42.0 ppm **(3 d)**. The chemical equivalence of the *tert‐*butyl phosphanyl groups in the ^31^P‐NMR spectra is indicative for the *meso*‐form with a *cis* configuration in which both substituents are pointing to the same side of the [3]ferrocenophane ring. The corresponding ^77^Se resonance of **3 c** at 156.2 ppm is drastically deshielded by more than 460 ppm upon ring expansion compared with **2 c**, while the ^1^ *J*_SeP_ coupling drops to 164 Hz. The tellurium expanded [3]ferrocenophane **3 d** shows a ^125^Te resonance at 157.0 ppm which is split into a triplet arising from ^1^ *J* ${}_{{}^{125}\mathrm{TeP}}$ coupling. Two sets of satellites are found in the ^31^P{^1^H} NMR spectrum owing to the two ^123^Te (^1^ *J* ${}_{{}^{123}\mathrm{TeP}}$ =263 Hz) and ^125^Te (^1^ *J* ${}_{{}^{125}\mathrm{TeP}}$ =318 Hz) isotopologues (Figure 3).

**Figure 4:**
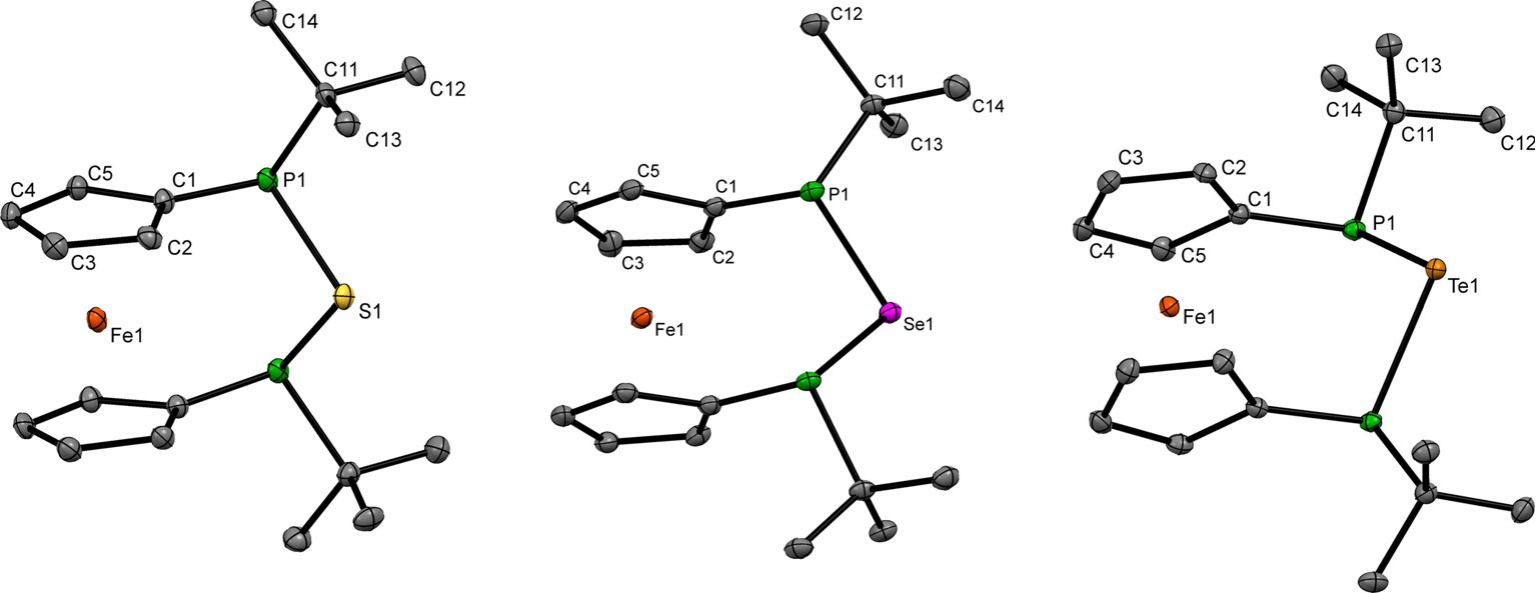
Solid‐state structure of **3 b** (left), **3 c** (middle) and **3 d** (right). Ellipsoids shown at 30 % probability. Important bond lengths and angles of **3 b**: twist angle *δ*=0.3(3)°, ΣP1=305.5(4)°, ΣP2=306.2(4)°; bond lengths: P1−S1 2.1360(13) Å, P1−C11 1.874(4) Å; **3 c**: twist angle *δ*=0.4(2)°, ΣP1=307.5(3)°, ΣP2=305.2(3)°; bond lengths: P1−Se1 2.2771(8) Å, P1−C11 1.882(3) Å; **3 d**: twist angle *δ*=1.1(3)°, ΣP1=308.5(4)°, ΣP2=305.9(4)°; bond lengths: P1−Te1 2.4894(8) Å, P1−C11 1.883(3) Å.

The findings for **3 b**–**d** in solution are corroborated for the solid state by single crystal X‐ray crystallography. The P‐Ch‐P angles of 91.50(5)° in **3 b**, 86.48(3)° in **3 c** and 82.63(3)° in **3 d** are close to the values observed for P‐E^II^‐P angles in divalent tetrylene adducts with [3]ferrocenophane scaffold (E=Si, Sn, Pb).^[14]^ The tilt angle (α) shows values of 4.6(2)° **(3 b)**, 3.6(2)° **(3 c)** and 2.9(2)° **(3 d)** which is in a typical range for phosphorus rich [3]ferrocenophanes.[^12a^, ^12b^, ^13b^, ^14^, ^15^] For compounds **3 b**‐**d** no intermolecular Ch⋅⋅⋅Ch interactions were observed (Figure 4).

**Scheme 5:**
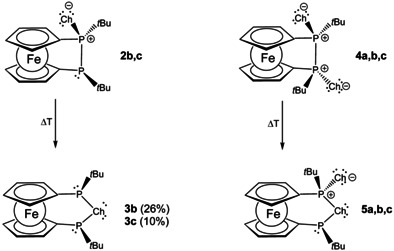
Thermal rearrangement of **2** to **3** (left; Ch=S (**b**), Se (**c**)) and **4** to **5** (right; Ch=O (**a**), S (**b**), Se (**c**)).

In light of the thermally induced rearrangement of diphosphanyl monochalcogenides **2 b,c**→**3 b,c**, outlined above, we wondered, whether twofold oxidized compounds **4** would behave likewise (Scheme 5). Indeed, thiodiphosphorane **4 b** rearranges in a similar and stereospecific fashion to **5 b**. The transformation seems to be much more facile since it occurs at lower temperature (170 °C) and is completed after a few hours. The rearrangement of the selenodiphosphorane **4 c** proceeds accordingly yielding **5 c**. Surprisingly, the isomerization of **4 c** takes place at higher temperature (150 °C) compared with **2 c**. In contrast to the monooxidized phosphorane **2 a** which shows no rearrangement to the corresponding diphosphanylchalcogane, a rearrangement of twofold oxidized **4 a** to **5 a** could be observed at high temperature (190 °C) over a period of several days.

Upon rearrangement, the ^31^P NMR chemical shift of **4 a** (68.3 ppm), **4 b** (81.8 ppm) and **4 c** (65.6 ppm) changes to lower field for the tetracoordinate phosphorus atom (Table 2), which is even more deshielded than in **2 a,b,c**. As expected, their trivalent counterparts in **5** resonate at higher field with ^2^ *J*_PP_ coupling constants in the range of 43–60 Hz (Table 2). These assignments are also consistent with ^77^Se NMR of **5 c**, showing a doublet of doublets at 366.6 ppm (^1^ *J*_SeP_=336 Hz and ^1^ *J*_SeP_=180 Hz) and a doublet of doublets at −200.3 ppm (^1^ *J*_SeP_=783 Hz and ^3^ *J*_SeP_=37 Hz), assigned to >P−Se−P≡ and ≡P=Se, respectively.

**Table 2:** ^31^P NMR chemical shifts and coupling constants of **5 a**–**c**.

	δ(^31^P) [ppm]		^2^ *J*_PP_ [Hz]
	P^III^	P^V^	
**5 a**	41.6	141.4	60
**5 b**	52.3	89.8	43
**5 c**	68.8	74.2	56

**Figure 5:**
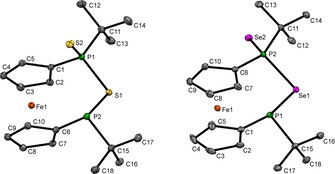
Molecular structure of **5 b** (left) and **5 c** (right) in the solid state. Ellipsoids shown at 30 % probability. Important bond lengths and angles of **5 b**: twist angle *δ*=3.3(2)°; tilt angle *α*=3.4(8)°; bond lengths: P1−S1 2.1031(6) Å, P1−S2 1.9487(6) Å, P1−C11 1.8543(17) Å; **5 c**: twist angle *δ*=6.8(2)°; tilt angle *α*=3.29(12)°; bond lengths: P1−Se1 2.2927(7) Å, P2−Se1 2.2553(6) Å, P2−Se2 2.1021(7) Å, P2−C11 1.854(2) Å.

Single crystal X‐ray diffraction of **5 b** and **5 c** (Figure 5) shows shorter P‐Ch contacts for the chalcogenophosphorane unit (**5 b**: 1.9487(6) Å (P1‐S2), **5 c**: 2.1021(7) Å (P2‐Se2)) as compared to the contacts associated with the bridging chalcogen atoms (2.1031(6) Å (P1‐S1), **5 b**; 2.2552(6) Å (P1‐Se1), **5 c**). These parameters are very close to the respective ones in **2**, **3** and **4**, as are the P‐Ch‐P angles (95.73(2)°, **5 b**; 93.61(2)°, **5 c**) indicating no significant ring strain in **5 b**,**c**.

### Mechanism

To understand the above mentioned chalcogen‐transfer rearrangement, we set out to explore the energetics of the reaction and its intermediates for the transformation **2**→**3**, described above. Investigating the mechanism of the insertion with DFT methods, established that the process is slightly exothermic and in general **3** is more stable than **2**. The energetic preference increases in the order **a<b<c<d**. (The calculated values vary between 11.3—(−30.5) kJ mol^−1^, depending on the applied DFT functionals Table S4–S6 in the Supporting Information). Firstly, we considered a monomolecular reaction mechanism for the transformation **2 b**→**3 b** as depicted in Scheme S1 in the Supporting Information. (Similar processes were calculated for **2 c**→**3 c** and **2 d**→**3 d** transformations, tabulated data in Table S4–S6 in the Supporting Information). The rate limiting step is the chalcogen insertion and the reaction barrier of this step (190.8 kJ mol^−1^) is comparable with the bonding energy of the P‐P bond (∼200 kJ mol^−1^). Similar mechanism and slightly lower barriers were obtained for the **4**→**5** rearrangements (more details in the Supporting Information Scheme S4). It should be highlighted that these barriers should be thermally accessible at the reaction temperature (over 100 °C), but do not rule out other mechanistic pathways. Besides a hypothetic intramolecular rearrangement also a bimolecular mechanism needs to be considered. In the literature a concerted rearrangement has been proposed for the rearrangement of acyclic diphosphane monochalcogenide to bisphosphanylchalcogane.^[8]^ Unfortunately, all our attempts to localize the corresponding transition states for the concerted bimolecular rearrangements failed in our hands. Based on the optimized molecular structures, steric repulsion between the *tert‐*butyl groups and the ferrocene moieties precludes a concerted bimolecular mechanism showing no energetic preference over the monomolecular rearrangement outlined in Scheme S1.

**Scheme 6:**
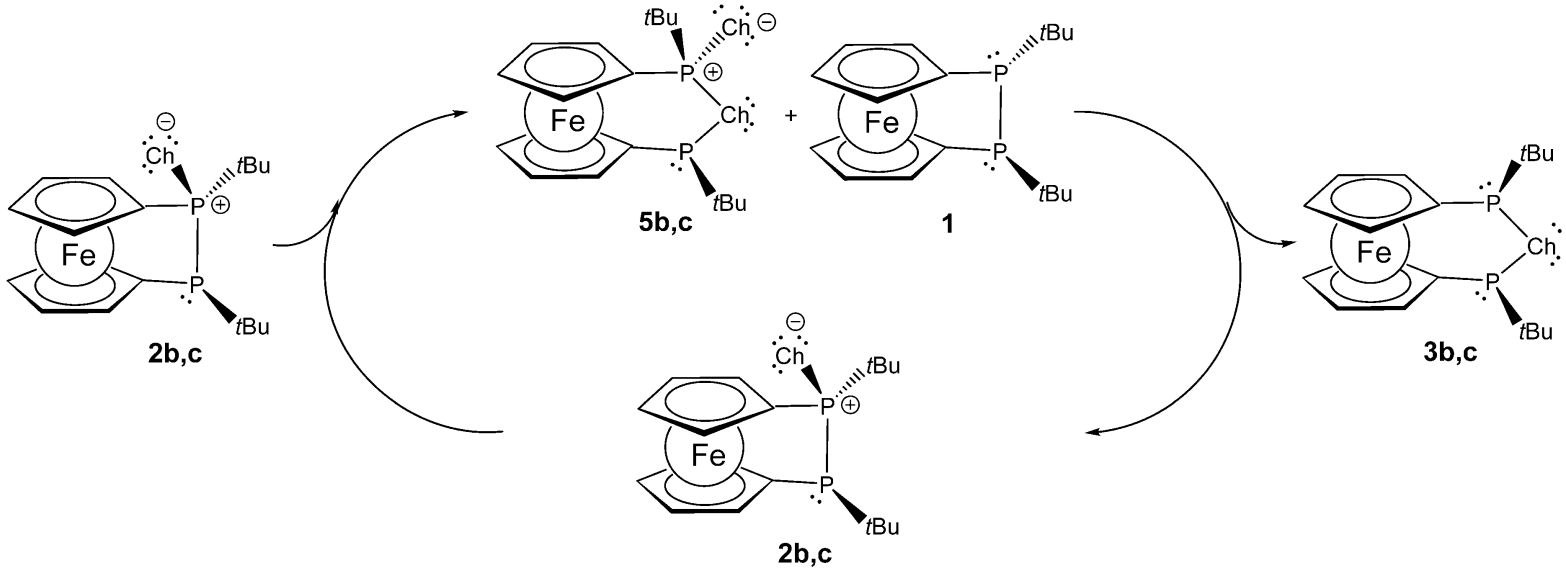
Disproportionation/synproportionation mechanism for chalcogen‐transfer rearrangement **2**→**3** (Ch=S (**b**), Se (**c**)).

**Figure 6:**
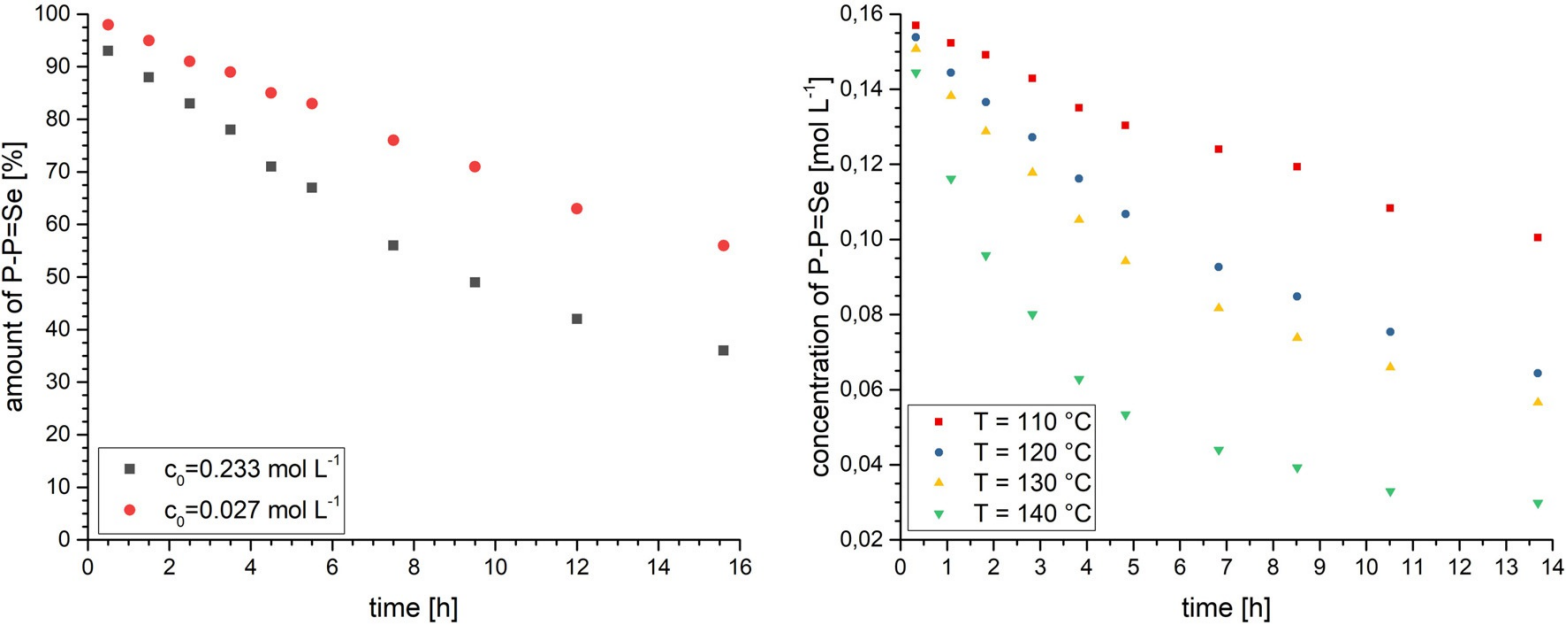
Concentration dependence of **2 c**→**3 c** at two different starting concentrations of **2 c** (*T*=120 °C; left). Temperature dependence of the rearrangement **2 c**→**3 c** over time with a starting concentration c_0_(**2 c**)=0.157 mol L^−1^ (right).

In support of a stepwise (non‐concerted) rearrangement, tracking the transformation **2 b**→**3 b** via ^31^P‐NMR spectroscopy revealed the transient occurrence of intermediate **5 b** along with desulfurized **1** in equal amounts. The identity of intermediately formed **5 b** has been confirmed by comparison with independently synthesized **5 b** (v.i.). Similarly, for the selenium transfer rearrangement **2 c**→**3 c**, tracking the rearrangement by NMR spectroscopy confirmed **5 c** along with deselenised **1** as intermediates. Consistent with the proposed reaction pathway, comproportionation of isolated **5 c** and **1** afforded **3 c** together with **2 c**, which were formed in equal amounts. Therefore, we conclude a disproportionation/synproportionation type mechanism for the chalcogen‐transfer rearrangement under discussion (Scheme 6). The bimolecular nature of the rate determining step of this non‐concerted chalcogen‐transfer rearrangement was established by NMR studies, running the reaction **2 c**→**3 c** at different concentrations which confirmed a second‐order kinetics for selenium transfer (Figure 6).

To enlighten the chalcogen‐transfer rearrangement also the temperature dependence of **2 c**→**3 c** was tracked by ^31^P‐NMR (Figure 6), which allows us to determine the power law (second order reaction) and rate constants *K*_T_ (0.75(2)×10^−4^ L mol^−1^⋅s^−1^ (*T*=110 °C); 1.89(2)×10^−4^ L mol^−1^⋅s^−1^ (*T*=120 °C); 2.34(3) ×10^−4^ L mol^−1^⋅s^−1^ (*T*=130 °C); 5.82(3)×10^−4^ L mol^−1^⋅s^−1^ (*T*=140 °C)) for the selenium transfer rearrangement. Plotting ln(K) against 1/T results in a linear relation, which allows the determination of the activation energy (*E*_A_=83(±13) kJ mol^−1^) from the slope of the linear regression (Figure S10, the Supporting information).

The suggested non‐concerted bimolecular mechanism was investigated computationally as well. We have considered different pathways (more details in the Supporting Information) and generally it could be established that, the chalcogen transfer steps exhibit moderate barriers (Δ*G* ^#^=57.7–69.0 kJ mol^−1^) for Ch=Se, which is comparable with the experimentally obtained value and the bimolecular nature of the rate limiting step. On the other hand, it should be noted that in all investigated cases the chalcogen insertion into the P‐P bond remain over 160 kJ mol^−1^ (Scheme S4 in Supporting Information), which is not consistent with our experimental observations.

To resolve this ambiguity, further calculation and experiments were carried out. TD‐DFT calculations indicate electron transfer from the lone pairs of the chalcogens towards the P‐P antibonding orbital in case of **2 b**,**c** and **4 b**,**c**, which results in elongation of the P‐P bond. Therefore, possible photoactivation cannot be excluded. Despite the promising DFT results, running the reaction **2 c** → **3 c** at different temperatures under exclusion of light (brown glass NMR tubes; Figure S11, the Supporting Information) reveal no significant influence by light and the determined value of the activation energy (*E*_A_ = 76 (±3) kJ mol^−1^) is comparable with the prior one. Also running the reaction **2 c**→**3 c** at room temperature, but under illumination with UV‐light (Hg‐lamp, 15 W) shows no [3]ferrocenophane formation after several hours of illumination.

Hypothetically the oxidant properties of chalcogens, may furnish oxidation of the ferrocene unit which subsequently may undergo electron transfer with the neighbouring diphosphane unit, thus giving rise to radical species even in the absence of light. Therefore, we explored the redox properties of central compounds of this investigation by cyclic voltammetry (CV). In the proposed disproportionation/synproportionation mechanism (Scheme 6) diphosphane **1** is oxidized by **5**, therefore we explored the electrochemical oxidation behaviour of **1** as a starting point. Diphosphane **1** shows two distinct oxidation events with peak potentials at 0.29(1) V and 0.97(1) V (vs. Fc^+^/Fc; blue line in Figure S1). The first current response shows reversible redox behaviour, if the voltage sweep is reverted before the second redox process. The second current response is reversible as well. However, with decreasing sweeping rate upon going through both redox processes in a single potential sweep, the nature of both processes and the first redox process in particular becomes irreversible in the reduction sweep (Figure S1). As iron or phosphorus centred oxidation is known to be feasible in related compounds,[^12b^, ^15d^] DFT calculations were performed where the calculated spin density (at B3LYP/6–311G**//*ω*B97XD/6–311+G**) of **1^+^** is localized at the ferrocene unit. Similarly, the CVs of chalcogeno and dichalcogeno phosphoranes **2 a**–**c** and **4 a**–**c** have been recorded (Figures S2–3 and Figure S5, The Supporting Information), indicating reversible iron centred oxidation as the primary redox event for most compounds (**2 a**: 0.43(2) V; **4 a**: 0.54(1) V; **2 b**: 0.48(1) V; **4 b**: 0.61(1) V; all vs. Fc^+^/Fc). The assignment to iron centred oxidation is in accordance with the calculated spindensity of the corresponding radical cations. An exception are selenophosphoranes **2 c** and **4 c** showing irreversible electro‐chemical responses for the oxidation (Figure S2 and S3) The reason of this observation can be explained with the increased energy level of the lone pairs of the selenium (compared to S or O), which have more significant contribution to the HOMO, than the ferrocene unit (Figure S7). The impact of the rearrangement **2**→**3** on the electronic situation of the ferrocene unit may also be assessed by CV for the sulfur species where the ferrocene‐based oxidation peak potential changes from 0.48(2) V (vs. Fc^+^/Fc) for **2 b** to 0.32(1) V (vs. Fc^+^/Fc) for **3 b**. In agreement with the reversible process the Fe centre has significant contribution to the spindensity distribution of the corresponding radical **3 b** ^+^. Selenium analogue **3 c** reveals a ferrocene‐centred oxidation at a peak potential of 0.33(1) V (vs. Fc^+^/Fc) as well, which is reversible if the potential sweep is switched to reversed scan direction right after first oxidation by avoiding the oxidation of the P‐Se‐P scaffold. The tellurium analogue **3 d** shows a quasi‐reversible ferrocene based redox process at a peak potential of 0.37(2) V (vs. Fc^+^/Fc), if the scan direction is switched right after the oxidation (Figure S4). In summary, the electrochemical investigation outlined above give no indication for redox based radical formation during oxidation and rearrangement of **1** or **2** with chalcogens. As a final check to prove or disprove the involvement of radical species in the thermal chalcogen‐transfer rearrangement **2**→**3** we performed the reaction in an EPR spectrometer at high temperature in a sealed EPR tube which did not give any indication for radical formation, unlike in other cases.^[12b]^

## Conclusion

We reported the addition versus insertion of chalcogens to a stereochemically confined diphosphane **1** taking advantage of the ferrocenophane scaffold. The resulting diphosphane monochalcogenides **2** undergo a chalcogen‐transfer rearrangement to the corresponding diphosphanylchalcoganes **3** for S and Se. The analogous behaviour is observed for twofold oxidized chalcogenodiphosphoranes **4** which rearrange stereospecifically to asymmetrically substituted **5**. The mechanism of the chalcogen‐transfer rearrangement was found to be bimolecular and non‐concerted based on NMR and DFT studies. Detection and identification of by‐products during rearrangement suggest a disproportionation/synproportionation mechanism for **2 b**→**3 b**, while no evidence for radical intermediates or photoactivation could be found. The general relevance of our results emerges from the prevalence of P^III^−P^III^ units in all phosphorus allotropes with the competition of addition to P^III^ atoms versus insertion into P‐P‐bonds being of utmost importance for P_4_ activation or phosphorene functionalization being a surging topic in sustainable and materials chemistry. In future work we intend to generalize the stereospecific addition/insertion of other amphiphilic species into P−P bonds of moderately strained oligophosphanes aiming at new routes to chiral organophosphanes.

## Conflict of interest

The authors declare no conflict of interest.

## Supporting information

As a service to our authors and readers, this journal provides supporting information supplied by the authors. Such materials are peer reviewed and may be re‐organized for online delivery, but are not copy‐edited or typeset. Technical support issues arising from supporting information (other than missing files) should be addressed to the authors.

Supplementary
