# Supplementary material for: Chalcogen‐Transfer Rearrangement: Exploring Inter‐ versus Intramolecular P−P Bond Activation
Source: Chemistry. 2020 Nov 9;27(2):641–8. doi: 10.1002/chem.202002481 (PMC7839705; doi:10.1002/chem.202002481)
Supplement: Supplementary file 1 — Supplementary [file CHEM-27-641-s001.pdf]

Chemistry–A European Journal

Supporting Information

Chalcogen-Transfer Rearrangement: Exploring Inter- versus Intramolecular P–P Bond Activation

Roman Franz,[a] Sina Nasemann,[a] Clemens Bruhn,[a] Zsolt Kelemen,*[b] and Rudolf Pietschnig*[a]

Content:

a) Experimental procedures
b) Synthetic protocols and characterization
c) Electrochemical measurements
d) X-ray crystallography
e) Computational details
f) Kinetic investigations
g) NMR spectra
h) XYZ coordinates and total energies of the investigated systems
i) References

Experimental procedures

All reactions were carried out by means of standard Schlenk or glovebox techniques under inert gas atmosphere (argon). Solvents were dried over Na/K alloy before use and were freshly distilled under inert gas. Deuterated solvents for NMR-spectroscopy were dried and stored over molecular sieves. $[Fe(C_5H_4P(t\text{Bu})H)_2]$[12b] and red selenium[16] were prepared according to the literature while other reagents were used as received without further purification. ^{1}H-, ^{13}C-, ^{31}P-, ^{77}Se- and ^{125}Te-NMR-data was recorded on Varian VNMRS-500 MHz or MR-400 MHz spectrometers at 25°C. Chemical shifts were referenced to residual protic impurities in the solvent (^{1}H) or the deuterio solvent (^{13}C) and reported relative to external $SiMe_4$ (^{1}H, ^{13}C), H_3PO_4 (^{31}P), Me_2Se (^{77}Se) or Me_2Te (^{125}Te). APCI-DIP mass determinations were performed on a Finnigan LCQ Deca (*ThermoQuest*). Mass calibration was carried out immediately before sample measurement on sodium formate clusters or by the ESI-Tune Mix standard (*Agilent*). Elemental analyses were performed with a HEKAtech Euro EA CHNS elemental analyzer. Samples were prepared in a Sn cup and analyzed with added V_2O_5. Cyclic voltammetric measurements were carried out in a MBraun acrylic glovebox GB2202-C-VAC under inert atmosphere (argon). The potential was driven on a WaveDriver 20 Bipotentiostat from Pine Research Instrumentation. Electrochemical data was recorded via AfterMath (Ver. 1.5.9807; *Pine Instruments*). The redox processes of all measurements were referenced using the half wave potentials and decamethyl ferrocene[17] as standard and evaluated with AfterMath and OriginPro (Ver. 8.6.0; *OriginLab Corporation*).

Crystallographic measurements were carried out on a *Stoe* IPDS2 or a *Stoe* StadiVari diffractometer with a STOE image plate detector and a Mo-Kα (λ = 0.71073 Å) monochromator or a *Stoe* StadiVari diffractometer with a Pilatus 200K image plate detector and Cu-Kα (λ = 1.54186 Å) radiation. Direct methods were used to solve the measurements and refined by "least-square" cycles (SHELXL-2017).[18] All non-hydrogen atoms were anisotropically refined without restriction. The evaluation of the data sets, as well as the graphical preparation of the structures was carried out using Olex2[19] and Mercury.[20] Details of the structure determinations and refinement are summarized in Table S1, Table S2 and Table S3. The CCDC depositions 2002689-2002698 contain the supplementary crystallographic data for this paper, which can be obtained free of charge via emailing data_request@ccdc.cam.ac.uk, or by contacting The Cambridge Crystallographic Data Centre at 12 Union Road, Cambridge CB2 1EZ, UK; fax: +44 1223 336033.

Synthetic protocols and characterization

Synthesis of 1

To a solution of 1,1'-Bis(*t*-butylphosphanyl)ferrocene (2.89 g, 8 mmol) in 20 mL toluene, a solution of *n*-BuLi in hexane (7.2 mL, 18 mmol, 2.5 M) and TMEDA (2.7 mL, 18 mmol) were added at room temperature. This mixture was stirred for 2 h. The solvent was removed under reduced pressure and the remaining solid was washed with 20 mL pentane. The solid was dissolved in 20 mL THF and was added dropwise to a suspension on 2.78 g (10 mmol) $PbCl_2$ which was cooled to -80°C. The mixture was allowed to warm to room temperature and was stirred overnight. All volatile components were removed *in vacuo*, the residue was extracted with 2x20 mL of toluene and the solid was removed and discarded. The toluene extract was evaporated to dryness yielding the raw product. Recrystallization from pentane at -20°C afforded 70% yield (2.05 g, 5.6 mmol) as orange crystals.

^{1}H-NMR (400 MHz, C_6D_6): δ 5.09 (m, 2H, Cp), 4.59 (m, 2H, Cp), 4.30 (m, 2H, Cp), 3.90 (m, 2H, Cp), 1.31 (pseudo t, J_{PH} = 6.6 Hz, 18H, *t*Bu CH_3). ^{13}C-NMR (101 MHz, C_6D_6): δ 82.6 (pseudo t, J_{PC} = 22.2 Hz, Cp), 75.9 (s, Cp), 75.1 (s, Cp), 70.7 (pseudo t, J_{PC} = 6.2 Hz, Cp), 68.7 (pseudo t, J_{PC} = 13 Hz, Cp C_{ipso}), 31.6 (pseudo t, J_{PC} = 2 Hz, *t*Bu C_q), 29.9 (pseudo t, J_{PC} = 10 Hz, *t*Bu CH_3). ^{31}P-NMR (202 MHz, C_6D_6): δ 20.3 ppm (s).

Synthesis of 2a

To a solution of **1** (72 mg, 0.2 mmol) in 5 mL pentane, 0.35 mL of an aqueous solution of H_2O_2 (0.6 M) and 1 mL THF were added at room temperature. This mixture was stirred for 30 min and the organic Phase was extracted with pentane. The pentane extract was dried with $MgSO_4$ and evaporated to dryness yielding the raw product. Recrystallization from pentane at -20°C afforded 40% yield (30 mg, 0.08 mmol) as orange crystals.

Alternative: to a solution of **1** (36 mg, 0.1 mmol) in 5 mL pentane, a solution of *t*-Butyl hydroperoxide (0.39 mL, 0.11 M) were added at room temperature. This mixture was stirred for 1 h and subjected to column separation on silica using diethyl ether (Rf ≈ 0.14) as eluent which afforded 80% yield (29 mg, 0.08 mmol) as orange crystals.

^{1}H-NMR (400 MHz, C_6D_6): δ 5.50 (m, 1H, Cp), 5.36 (m, 1H, Cp), 4.58 (m, 1H, Cp), 4.46 (m, 1H, Cp), 4.31 (m, 1H, Cp), 4.12 (m, 1H, Cp), 3.99 (m, 1H, Cp), 3.91 (m, 1H, Cp), 1.40 (m, 9H, P-*t*Bu CH_3), 1.26 (m, 9H, O=P-*t*Bu CH_3). ^{13}C-NMR (101 MHz, C_6D_6): δ 83.3 (m, Cp), 78.3 (m, Cp), 77.9 (m, Cp), 76.1 (m, Cp), 73.2 (m, Cp), 72.0 (m, Cp), 71.5 (m, Cp), 71.0 (m, Cp), 64.5 (m, Cp C_{ipso}), 64.3 (m, Cp C_{ipso}), 38.5 (m, O=P-*t*Bu C_q), 35.6 (m, P-*t*Bu C_q), 30.2 (m, P-*t*Bu CH_3), 25.2 (m, O=P-*t*Bu CH_3). ^{31}P-NMR (202 MHz, C_6D_6): δ 79.6 (d, $^1J_{PP}$ = 272 Hz, O=P-*t*Bu), 7.3 (d, $^1J_{PP}$ = 271 Hz, P-*t*Bu). Elemental analysis (%): calculated: C 57.47, H 6.97, found: C 57.27, H 6.87. MS (APCI-DIP-HR) m/z: 377.088107 ($[M+H]^+$ 100 %), calculated: 377.088122.

Synthesis of 2b

To a solution of **1** (90 mg, 0.25 mmol) in 5 mL toluene, 8 mg sulfur were added at room temperature. This mixture was stirred overnight. All volatile components were removed *in vacuo*, the residue was extracted with 20 mL of diethylether and the solid was removed and discarded. The raw product was subjected to flash column separation on silica using a mixture of pentane and methylene chloride as eluent which afforded 53% yield (56 mg, 0.13 mmol) as orange crystals.

^{1}H-NMR (400 MHz, C_6D_6): δ 5.75 (m, 1H, Cp), 5.54 (m, 1H, Cp), 4.76 (m, 1H, Cp), 4.45 (m, 1H, Cp), 4.34 (m, 1H, Cp), 4.18 (m, 1H, Cp), 3.94 (m, 1H, Cp), 3.88 (m, 1H, Cp), 1.35 (d, $^3J_{PH}$=14.4 Hz, 9H, P-*t*Bu CH_3), 1.33 (d, $^3J_{PH}$=17.2 Hz, 9H, S=P-*t*Bu CH_3). ^{13}C-NMR (101 MHz, C_6D_6): δ 84.0 (m, Cp), 83.5 (m, Cp), 80.6 (m, Cp), 78.7 (m, Cp), 76.6 (s, Cp), 74.5 (m, Cp), 73.9 (m, Cp C_{ipso}), 72.0 (m, Cp), 71.8 (m, Cp), 71.4 (m, Cp), 64.2 (dd, $^1J_{PC}$=23.6 Hz, $^2J_{PC}$ =5.8 Hz, Cp C_{ipso}), 40.5 (m S=P-*t*Bu C_q), 36.4 (dd, $^1J_{PC}$=19.1 Hz, $^2J_{PC}$ = 2.9 Hz, P-*t*Bu C_q), 29.3 (dd, $^2J_{PC}$=15.7 Hz, $^3J_{PC}$=5.9 Hz, P-*t*Bu CH_3), 26.0 (dd, $^2J_{PC}$=4.9 Hz, $^3J_{PC}$=2.7 Hz, S=P-*t*Bu CH_3). ^{31}P-NMR (202 MHz, C_6D_6): δ 86.2 (d, $^1J_{PP}$ = 301 Hz, S=P-*t*Bu), 15.6 (d, $^1J_{PP}$ = 301 Hz, P-*t*Bu). Elemental analysis (%): calculated: C 55.12, H 6.68, S 8.17, found: C 55.04, H 6.78 S 8.08. MS (APCI-DIP-HR) m/z: 393.065263 ($[M+H]^+$ 100 %), calculated: 393.065279.

Synthesis of 2c

To a solution of **1** (36 mg, 0.1 mmol) in 4 mL THF, 7.9 mg grey selenium powder were added at room temperature. This mixture was stirred overnight. All volatile components were removed *in vacuo*, the residue was extracted with 8 mL of pentane and the solid was removed and discarded. The pentane was evaporated to dryness which afforded 50% yield (22 mg, 0.05 mmol) as orange oil.

^{1}H-NMR (400 MHz, C_6D_6): δ 5.89 (m, 1H, Cp), 5.65 (m, 1H, Cp), 4.78 (m, 1H, Cp), 4.42 (m, 1H, Cp), 4.33 (m, 1H, Cp), 4.20 (m, 1H, Cp), 3.91 (m, 1H, Cp), 3.84 (m, 1H, Cp), 1.36 (m, 9H, P-*t*Bu CH_3), 1.35 (d, $^3J_{PH}$=17.4 Hz, 9H, Se=P-*t*Bu CH_3). ^{13}C-NMR (101 MHz, C_6D_6): δ 83.9 (m, Cp), 82.7 (m, Cp), 78.4 (m, Cp), 76.7 (m, Cp), 75.0 (s, Cp), 72.0 (m, Cp), 71.9 (m, Cp), 71.6 (dd, $^1J_{PC}$=50.6 Hz, $^2J_{PC}$=3.9 Hz, Cp C_{ipso}), 71.2 (m, Cp), 64.1 (dd, $^1J_{PC}$=24.0 Hz, $^2J_{PC}$=5.8 Hz, Cp C_{ipso}), 39.8 (dd, $^1J_{PC}$=30.9 Hz, $^2J_{PC}$=15.0 Hz Se=P-*t*Bu C_q), 36.7 (dd, $^1J_{PC}$=20.0 Hz, $^2J_{PC}$ = 2.7 Hz, P-*t*Bu C_q), 29.0 (dd, $^2J_{PC}$=15.8 Hz, $^3J_{PC}$=6.0 Hz, P-*t*Bu CH_3), 26.4 (dd, $^2J_{PC}$=4.8 Hz, $^3J_{PC}$=3.1 Hz, Se=P-*t*Bu CH_3). ^{31}P-NMR (202 MHz, C_6D_6, rt): 71.3 (d, $^1J_{PP}$ = 309

Hz, Se=P-*t*Bu), 18.3 (d, $^{1}J_{PP}$ = 309 Hz, P-*t*Bu). ^{77}Se-NMR (95 MHz, C_6D_6): δ -311.7 (dd, $^{1}J_{PSe}$=725 Hz, $^{2}J_{PSe}$=30 Hz). Elemental analysis (%): calculated: C 49.23, H 5.97, found: C 49.29, H 6.27. MS (APCI-DIP-HR) m/z: 441.009858 ($[M+H]^+$ 100 %), calculated: 441.009714.

Synthesis of 3b

A solution of **2b** (78 mg, 0.2 mmol) in 2 mL mesitylene was stirred at 250°C over a period of 2 weeks, until all starting material was converted which was investigated by ^{31}P-NMR. All volatile components were removed *in vacuo*, the residue was extracted with 10 mL of pentane and the solid was removed and discarded. The raw product was subjected to flash column separation on silica using a mixture of pentane and diethylether as eluent which afforded 26% yield (21 mg, 0.05 mmol) as orange crystals.

^{1}H-NMR (400 MHz, C_6D_6): δ 4.68 (m, 2H, Cp), 4.53 (m, 2H, Cp), 4.11 (m, 2H, Cp), 3.89 (m, 2H, Cp), 1.26-1.21 (m, 18H, *t*Bu CH_3). ^{13}C-NMR (101 MHz, C_6D_6): δ 82.8 (m, Cp C_{ipso}), 80.6 (m, Cp), 72.3 (m, Cp), 71.1 (m, Cp), 70.0 (m, Cp), 32.6 (m, *t*Bu C_q), 28.7 (m, *t*Bu CH_3). ^{31}P-NMR (202 MHz, C_6D_6): δ 51.8 (s). Elemental analysis (%): calculated: C 55.12, H 6.68, S 8.17, found: C 54.96, H 6.74, S 8.22. MS (APCI-DIP-HR) m/z: 393.065279 ($[M+H]^+$ 100 %), calculated: 393.065263.

Synthesis of 3c

A solution of **2c** (78 mg, 0.2 mmol) in 2 mL mesitylene was stirred at 120°C for few hours, until all starting material was converted which was investigated by ^{31}P-NMR. All volatile components were removed *in vacuo*, the residue was extracted with 10 mL of pentane and the solid was removed and discarded. The raw product was subjected to flash column separation on silica using a mixture of pentane and methylene chloride as eluent which afforded 10% yield (9 mg, 0.02 mmol) as orange crystals.

Alternative: to a solution of **1** (180 mg, 0.5 mmol) in 4 mL THF, 40 mg red selenium powder were added at room temperature. This mixture was stirred overnight. All volatile components were removed *in vacuo*, the residue was extracted with 10 mL of pentane and the solid was removed and discarded. The pentane extract was evaporated to dryness yielding the raw product. Recrystallization from pentane at -20°C afforded 48% yield (42 mg, 0,09 mmol) as orange crystals.

^{1}H-NMR (400 MHz, C_6D_6): δ 4.68 (m, 2H, Cp), 4.55 (m, 2H, Cp), 4.12 (m, 2H, Cp), 3.86 (m, 2H, Cp), 1.23 (m, 18H, *t*Bu CH_3). ^{13}C-NMR (101 MHz, C_6D_6): δ 82.2 (m, Cp C_{ipso}), 80.6 (m, Cp), 72.4 (m, Cp), 72.3 (m, Cp), 69.8 (m, Cp), 32.8 (m, *t*Bu C_q), 29.2 (m, *t*Bu CH_3). ^{31}P-NMR (202 MHz, C_6D_6): δ 54.6 (s). ^{77}Se-NMR (95 MHz, C_6D_6): δ 156.2 (t, $^{1}J_{PSe}$=164 Hz). Elemental analysis (%): calculated: C 49.23, H 5.97, found: C 49.23, H 6.25. MS (APCI-DIP-HR) m/z: 441.009714 ($[M+H]^+$ 100 %), calculated: 441.009858.

Synthesis of 3d

To a solution of **1** (90 mg, 0.25 mmol) in 2 mL THF, 64 mg tellurium powder were added at room temperature. This mixture was heated to 120°C in a pressure Schlenk-tube and stirred overnight at that temperature. After cooling to room temperature all volatile components were removed *in vacuo*, the residue was extracted with 10 mL of pentane and the solid was removed and discarded. The raw product was subjected to flash column separation on silica using a mixture of pentane and methylene chloride as eluent which afforded 11% yield (14 mg, 0.03 mmol) as yellow crystals. Owing to potential light sensitivity of **3d** which may lead to elimination of elemental tellurium, the reaction was repeated in brown glassware protecting it from visible light, which had no impact on the outcome of the reaction, however.

^{1}H-NMR (400 MHz, C_6D_6): δ 4.67 (m, 2H, Cp), 4.60 (m, 2H, Cp), 4.14 (m, 2H, Cp), 3.81 (m, 2H, Cp), 1.20 (m, 18H, *t*Bu CH_3). ^{13}C-NMR (101 MHz, C_6D_6): δ 81.6 (m, Cp C_{ipso}), 80.4 (m, Cp), 75.3 (m, Cp), 72.4 (m, Cp), 69.8 (m, Cp), 33.2 (m, *t*Bu C_q), 30.2 (m, *t*Bu CH_3). ^{31}P-NMR (202 MHz, C_6D_6): δ 42.0 (s). ^{125}Te-NMR (158 MHz, C_6D_6): δ 157.0 (t, $^{1}J_{PTe}$=318

Hz). Elemental analysis (%): calculated: C 44.32, H 5.37, found: C 44.67, H 5.65. MS (APCI-DIP-HR) m/z: 490.999513 ($[M+H]^+$ 100 %), calculated: 490.999415.

Synthesis of 4a

To a solution of **1** (72 mg, 0.2 mmol) in 5 mL pentane, one drop of an aqueous solution of H_2O_2 (30 wt. %) and 1 mL THF were added at room temperature. 40 mL of diethylether were added to the mixture. The organic phase was extracted twice with water in a separatory funnel and was dried over anhydrous $MgSO_4$ yielding the raw product. Recrystallization from diethylether at -20°C afforded 87% yield (68 mg, 0.17 mmol) as orange crystals.

^{1}H-NMR (400 MHz, C_6D_6): δ 5.31 (m, 2H, Cp), 5.03 (m, 2H, Cp), 4.16 (m, 2H, Cp), 3.97 (m, 2H, Cp), 1.36 (m, 18H, *t*Bu CH_3). ^{13}C-NMR (101 MHz, C_6D_6): δ 77.9 (m, Cp), 75.3 (m, Cp), 72.4 (m, Cp), 72.3 (m, Cp), 71.8 (m, Cp C_{ipso}), 39.4 (m, *t*Bu C_q), 24.8 (m, *t*Bu CH_3). ^{31}P-NMR (202 MHz, C_6D_6): δ 68.3 (s). Elemental analysis (%): calculated: C 55.12, H 6.68, found: C 55.21, H 6.64. MS (APCI-DIP-HR) m/z: 393.083022 ($[M+H]^+$ 100 %), calculated: 393.083038.

Synthesis of 4b

To a solution of **1** (78 mg, 0.22 mmol) in 3 mL toluene, 15 mg sulfur were added at room temperature. This mixture was stirred several hours at 110°C. The reaction mixture was subjected to flash column separation on silica using pentane as mobile phase to remove unreacted sulfur. An orange band was eluted with ethyl acetate which afforded 23% yield (18 mg, 0.04 mmol) as orange crystals.

^{1}H-NMR (400 MHz, C_6D_6): δ 5.56 (m, 2H, Cp), 5.45 (m, 2H, Cp), 4.24 (m, 2H, Cp), 3.87 (m, 2H, Cp), 1.54-1.47 (m, 18H, *t*Bu CH_3). ^{13}C-NMR (101 MHz, C_6D_6): δ 81.2 (m, Cp), 76.2 (m, Cp), 75.8 (m, Cp), 71.6 (m, Cp), 70.6 (m, Cp C_{ipso}), 45.1 (m, *t*Bu C_q), 26.4 (s, *t*Bu CH_3). ^{31}P-NMR (202 MHz, C_6D_6): δ 81.8 (s). Elemental analysis (%): calculated: C 50.95, H 6.18, S 15.11, found: C 51.15, H 6.31, S 14.94. MS (APCI-DIP-HR) m/z: 425.037334 ($[M+H]^+$ 100 %), calculated: 425.038283.

Synthesis of 4c

To a solution of **1** (36 mg, 0.1 mmol) in 4 mL THF, 20 mg grey selenium powder were added at room temperature. This mixture was stirred 6h at room temperature and the solid was removed and discarded. All volatile components were evaporated to dryness yielding the raw product. Recrystallization from pentane at -20°C afforded 64% yield (33 mg, 0.06 mmol) as orange crystals.

^{1}H-NMR (400 MHz, C_6D_6): δ 5.78 (m, 2H, Cp), 5.53 (m, 2H, Cp), 4.29 (m, 2H, Cp), 3.83 (m, 2H, Cp), 1.58-1.51 (m, 18H, *t*Bu CH_3). ^{13}C-NMR (101 MHz, C_6D_6): δ 84.0 (m, Cp), 77.2 (m, Cp), 76.8 (m, Cp), 71.9 (m, Cp), 68.8 (m, Cp C_{ipso}), 46.2 (m, *t*Bu C_q), 27.4 (s, *t*Bu CH_3). ^{31}P-NMR (202 MHz, C_6D_6): δ 65.6 (s). ^{77}Se-NMR (95 MHz, C_6D_6): δ -275.6 (m, $^1J_{PSe}$=741 Hz, $^2J_{PSe}$=17 Hz). Elemental analysis (%): calculated: C 41.73, H 5.06, found: C 42.09, H 5.43. MS (APCI-DIP-HR) m/z: 520.926236 ($[M+H]^+$ 100 %), calculated: 520.926676.

Electrochemical measurements

All cyclic voltammetric measurements were carried out in a MBraun acrylic glovebox GB2202-C-VAC under inert atmosphere (argon). All samples were measured in anhydrous methylene chloride. Anhydrous tetrabutylammonium hexafluorophosphate ($[NBu_4][PF_6]$) was used as conducting salt at a concentration of 0.1 M, while the sample concentration was set to 0.1 mM. The three-electrode cell consisted of a platinum working electrode, a silver counter electrode and a silver pseudo reference electrode. The potential was driven on a WaveDriver 20 Bipotentiostat from *Pine Research Instrumentation*. Electrochemical data was recorded via AfterMath (Ver. 1.5.9807; *Pine Instruments*). The redox processes of all measurements were referenced using the half wave potentials and decamethyl ferrocene as standard and evaluated with AfterMath and OriginPro (Ver. 8.6.0; *OriginLab Corporation*).

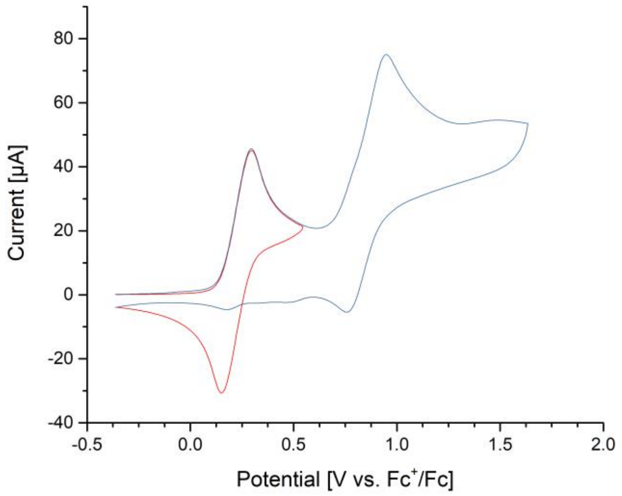

Figure S1: Cyclic voltammetry of **1** (referenced vs. Fc^{+}/Fc). Voltage sweep 250 mV/s.

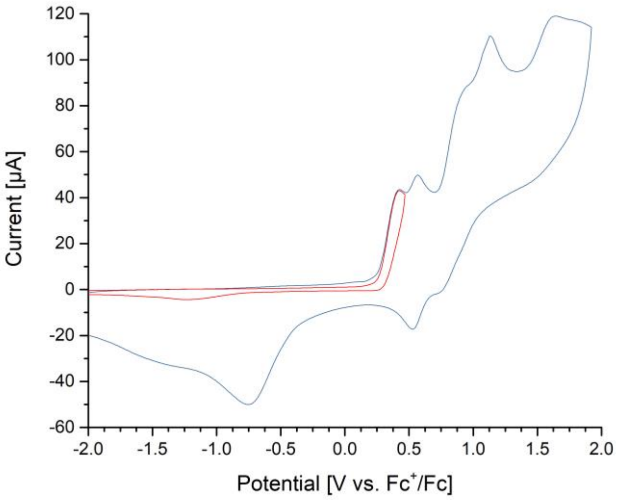

Figure S2: Cyclic voltammetry of **2c** (referenced vs. Fc^{+}/Fc). Voltage sweep 250 mV/s.

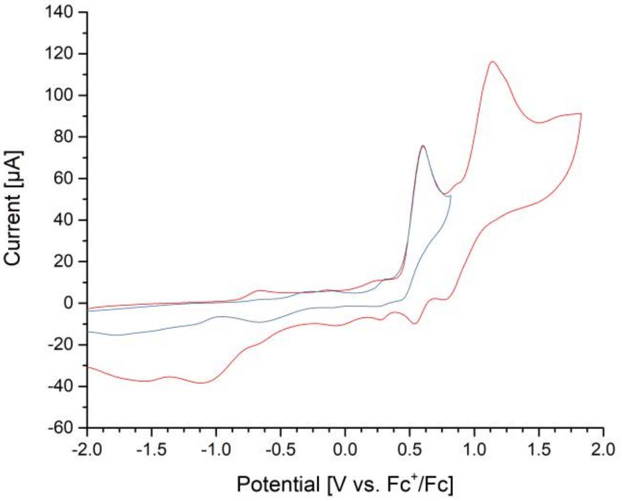

Figure S3: Cyclic voltammetry of **4c** (referenced vs. Fc^{+}/Fc). Voltage sweep 250 mV/s.

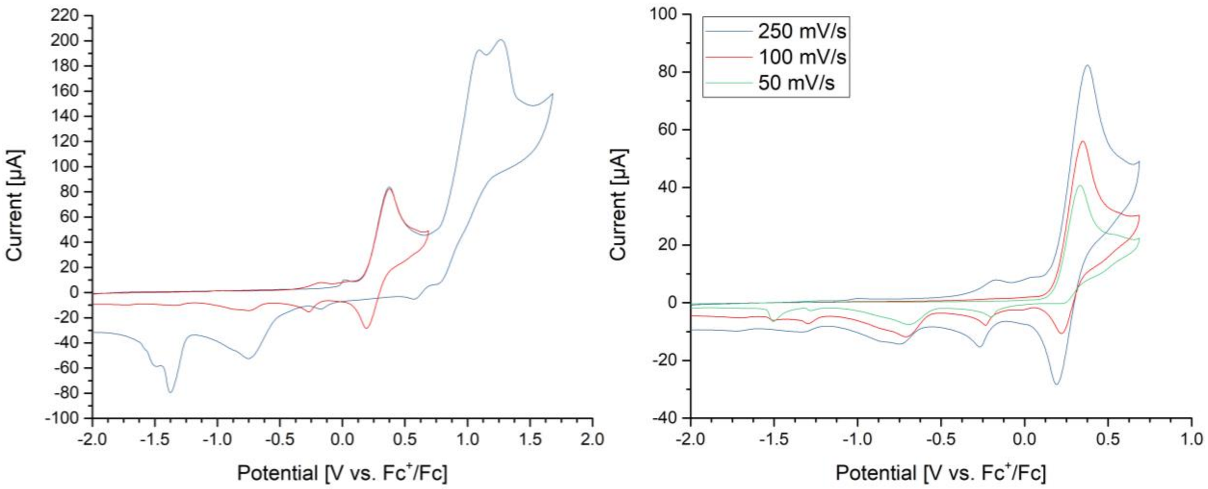

Figure S4: Cyclic voltammetry of **3d** (referenced vs. Fc^+/Fc) at a voltage sweep of 250 mV/s (left) and at different voltage sweep (250 mV/s (blue), 100 mV/s (red) and 50 mV/s (green)) (right).

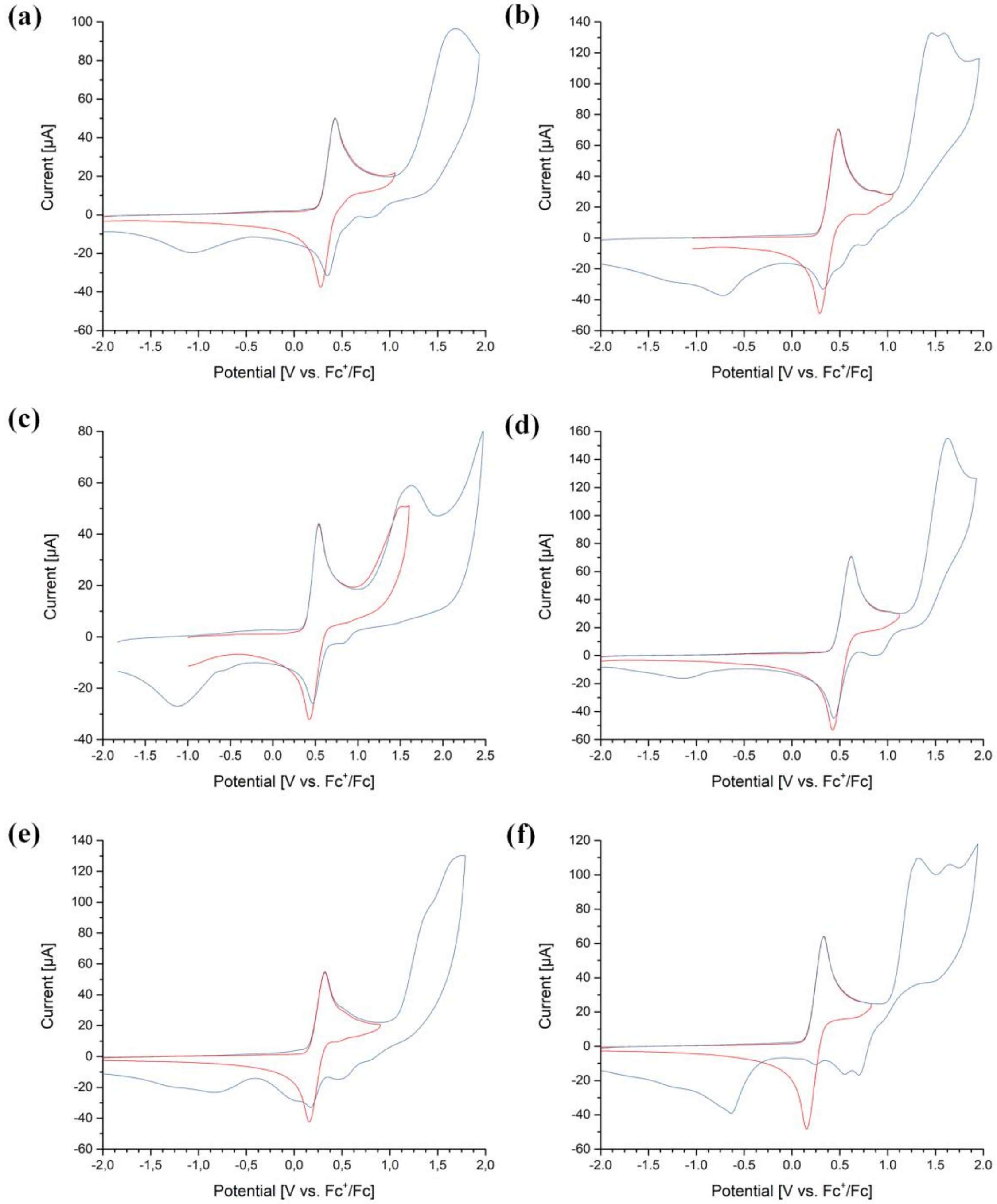

Figure S5: Cyclic Voltammogram of a 1 mM solution of **2a** (a), **2b** (b), **4a** (c), **4b** (d), **3b** (e) and **3c** (f) in dichloromethane at a scan rate of 250 mVs^{-1}. Blue line shows the whole voltammogram while the red line results from a measurement during which the potential sweep is switched to reversed potential after the first oxidation.

X-ray crystallography

Table S1: Summary of structure determinations and refinement for **2a**, **2b**, **3a** and **3b**.

	**2a**	**2b**	**4a**	**4b**
CCDC code	2002692	2002697	2002694	2002696
Empirical formula	$C_{18}H_{26}FeOP_2$	$C_{18}H_{26}FeP_2S$	$C_{18}H_{26}FeO_2P_2$	$C_{18}H_{26}FeP_2S_2$
Formula weight [g/mol]	376.18	392.24	392.18	424.30
Crystal description	yellow plate	orange plate	orange plate	orange plate
Temperature [K]	100	100	100	100
Radiation and λ [Å]	Cu K_α, 1.54186	Mo K_α, 0.71073	Mo K_α, 0.71073	Mo K_α, 0.71073
Crystal system, space	monoclinic, $P2_1/c$	monoclinic, $P2_1/c$	trikilinic, $P\bar{1}$	monoclinic, $P2_1$/c
Unit cell dimensions:				
a [Å]	26.2806(8)	12.3909(10)	9.5389(5)	13.4204(9)
b [Å]	8.1594(2)	11.3866(7)	10.0204(5)	9.6126(4)
c [Å]	17.6428(6)	13.5095(9)	11.1761(6)	15.2091(11)
α [°]	90	90	89.807(4)	90
β [°]	109.480(2)	92.315(6)	70.780(4)	99.573(6)
γ [°]	90	90	65.186(4)	90
Volume [$Å^3$]	3566.66(19)	1904.5(2)	904.19(9)	1934.7(2)
Z	8	4	2	4
Crystal size [mm]	0.30x0.04x0.03	0.14x0.16x0.20	0.30x0.20x0.11	0.35 x 0.13x0.04
Calculated density [g/cm^3]	1.401	1.368	1.440	1.457
Linear absorption coefficient μ [mm^{-1}]	8.456	1.064	1.017	1.157
F(000)	1584	824	412	888
Θ-Range for data collection [°]	3.57-69.84	1.6-25.5	1.95 - 26.26	1.54-26.32
Index ranges	$-31 < h < 31$	$-15 < h < 15$	$-11 < h < 11$	$-16 < h < 16$
	$-4 < k < 9$	$-13 < k < 13$	$-11 < k < 12$	$-10 < k < 11$
	$-21 < l < 20$	$-16 < l < 16$	$-13 < l < 13$	$-18 < l < 18$
Refl. collected/unique	24114/6486	10481/3545	6388/3363	10720/3652
Completeness to Θ = 26.0°	0.990	0.999	0.997	0.999
Data/restraints/parameters	6486/0/410	3545/0/205	3363/0/214	3652/0/214
Goodness-of-fit on F^2	1.142	1.058	1.031	1.061
Final *R* indices [$I>2\delta(I)$]/ [wR_2]	0.0983/0.2490	0.0970/0.2701	0.0417/0.1174	0.0395/0.1025
R indices (all data)/ [wR_2]	0.1134/0.2569	0.1118/0.2901	0.0448/0.1201	0.0433/0.1052
Largest difference hole/peak [e $Å^{-3}$]	-0.77, 2.53	-0.58, 1.05	-0.68, 0.69	-0.61, 1.00

Table S2: Summary of structure determinations and refinement for **4c**, **3b**, **3c** and **3d**.

	**4c**	**3b**	**3c**	**3d**
CCDC code	2002690	2002693	2002689	2002695
Empirical formula	$C_{18}H_{26}FeP_2Se_2$	$C_{18}H_{26}FeP_2S$	$C_{18}H_{26}FeP_2Se$	$C_{18}H_{26}FeP_2Te$
Formula weight [g/mol]	518.10	392.24	439.14	487.78
Crystal description	red plate	orange block	yellow plate	yellow plate
Temperature [K]	100	100	100	100
Radiation and λ [Å]	Mo K_α, 0.71073	Mo K_α, 0.71073	Mo K_α, 0.71073	Cu K_α, 1.54186
Crystal system, space	monoclinic, $P2_1/c$	monoclinic, $P2_1/c$	monoclinic, $P2_1/c$	monoclinic, $P2_1/c$
Unit cell dimensions:				
a [Å]	15.4916(5)	6.4721(3)	11.1007(6)	11.3531(5)
b [Å]	9.5456(2)	20.4890(12)	16.1341(6)	16.1647(6)
c [Å]	26.7718(8)	14.4475(7)	11.7928(7)	11.9564(5)
α [°]	90	90	90	90.00
β [°]	95.342(2)	101.960(4)	116.870(4)	117.531(3)
γ [°]	90	90	90	90.00
Volume [Å^3]	3941.73(19)	1874.25(17)	1884.06(18)	1945.76(15)
Z	8	4	4	4
Crystal size [mm]	0.15x0.10x0.02	0.24x0.17x0.05	0.03x0.15x0.16	0.09x0.053x0.02
Calculated density [g/cm^3]	1.746	1.390	1.548	1.665
Linear absorption coefficient μ [mm^{-1}]	4.618	1.081	2.897	19.303
F(000)	2064	824	896	968
Θ-Range for data collection [°]	1.46-26.25	1.99-32.76	2. -25.5	4.44-70.6
Index ranges	$-18 < h < 15$	$-6 < h < 7$	$-13 < h < 13$	$-10 < h < 13$
	$-10 < k < 11$	$-25 < k < 25$	$-19 < k < 18$	$-19 < k < 15$
	$-32 < l < 32$	$-17 < l < 17$	$-14 < l < 14$	$-13 < l < 14$
Refl. collected/unique	15611/7407	10315/3638	10061/3511	8762/3637
Completeness to Θ = 26.0°	0.989	0.992	1.000	0.974
Data/restraints/parameters	7407/6/501	3638/0/205	3511/0/205	3637/0/205
Goodness-of-fit on F^2	1.113	1.085	1.153	1.041
Final R indices [I>2δ(I)]/ [wR_2]	0.0427/0.0933	0.0617/0.1676	0.0363/0.0996	0.0270/0.0623
R indices (all data)/ [wR_2]	0.0549/0.0995	0.0677/0.1846	0.0406/0.1028	0.0342/0.0657
Largest difference hole/peak [e Å^{-3}]	-0.52/0.99	-0.58/1.51	-1.29/1.55	-0.77/0.43

Table S3: Summary of structure determination and refinement for **5b** and **5c**.

	**5b**	**5c**
CCDC code	2002698	2002691
Empirical formula	$C_{18}H_{26}FeP_2S_2$	$C_{18}H_{26}FeP_2Se_2$
Formula weight [g/mol]	424.30	518.10
Crystal description	yellow block	yellow plate
Temperature [K]	100	100
Radiation and λ [Å]	Mo K_{α}, 0.71073	Mo K_{α}, 0.71073
Crystal system, space	monoclinic, $P2_1/c$	monoclinic, $P2_1/c$
Unit cell dimensions:		
a [Å]	11.3679(8)	10.9455(8)
b [Å]	12.5792(7)	12.1955(10)
c [Å]	14.5750(9)	15.2237(10)
α [°]	90	90
β [°]	104.347(5)	101.764(5)
γ [°]	90	90
Volume [$Å^3$]	2019.2(2)	1989.5(3)
Z	4	4
Crystal size [mm]	0.17x0.12x0.09	0.14x0.107x0.06
Calculated density [g/cm^3]	1.396	1.730
Linear absorption coefficient μ [mm^{-1}]	1.109	4.575
F(000)	888	1032
Θ-Range for data collection [°]	2.17-26.81	2.10-26.78
Index ranges	$-13 < h < 14$	$-13 < h < 13$
	$-15 < k < 15$	$-15 < k < 15$
	$-18 < l < 18$	$-17 < l < 19$
Refl. collected/unique	12682/4281	12482/4230
Completeness to Θ = 26.0°	0.991	0.996
Data/restraints/parameters	4281 /0/214	4230/0/214
Goodness-of-fit on F^2	1.041	1.046
Final R indices [I>2δ(I)]/ [wR_2]	0.0256/ 0.0649	0.0638/0.0669
R indices (all data)/ [wR_2]	0.0298/ 0.0693	0.0265/0.0330
Largest difference hole/peak [e $Å^{-3}$]	-0.27/0.41	-0.372/0.559

Computational details

All calculations were carried out with the Gaussian 09 program package[1] at ωB97XD/6-311+G** M06-2X/6-311+G** or BP86/6-311+G** level of theory as it was implemented in G09. Full geometry optimization calculations were performed, and harmonic vibrational frequencies were calculated to establish the nature of the stationary points obtained, as characterized by none negative eigenvalue of the Hessian for minima structures and one negative eigenvalue for transition states. For the visualization of the molecules and orbitals the MOLDEN[2] and VMD program[3] were used.

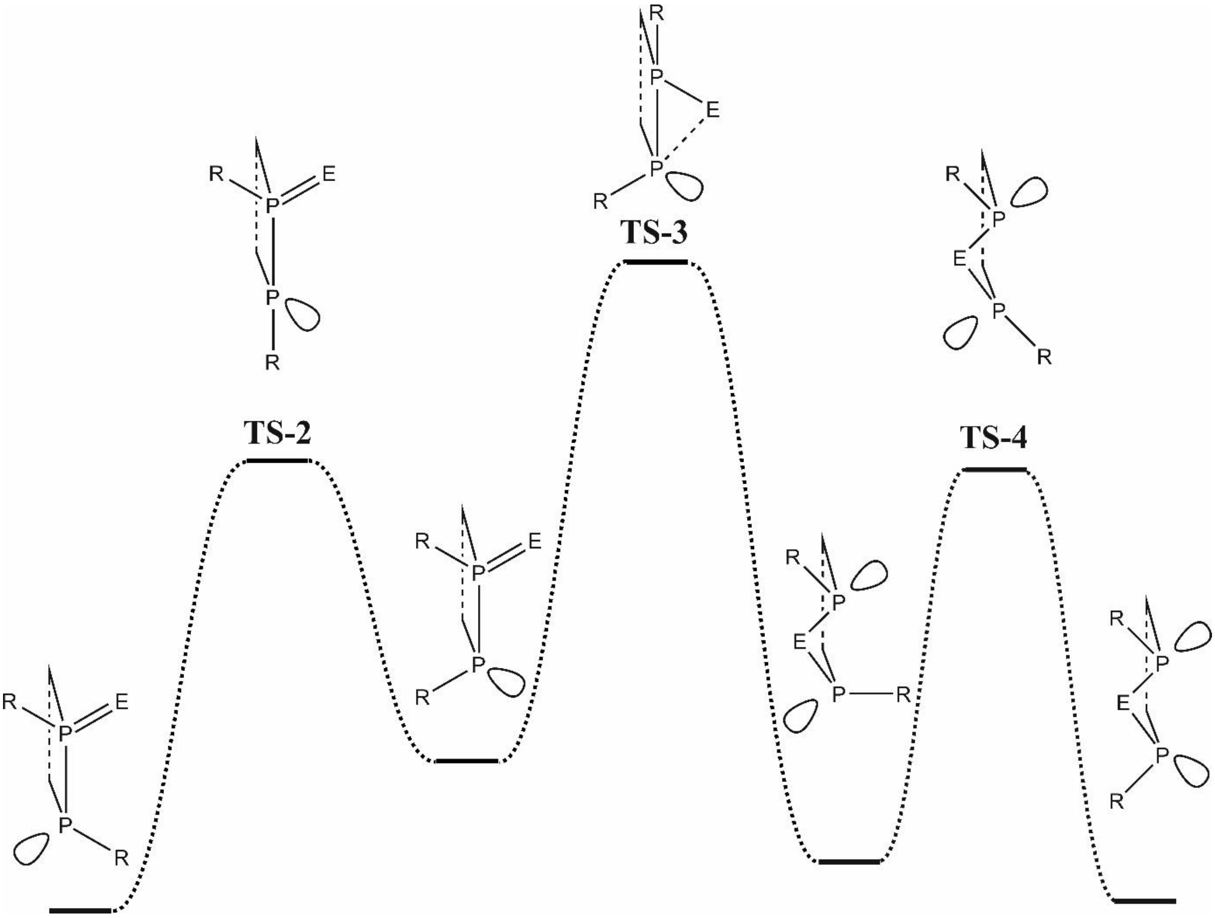

Scheme S1 Calculated reaction mechanism of the insertion process.

Table S4 Relative energies of the investigated species and the transition states between them at ωB97XD/6-311+G** level of theory and in kJ/mol unit.

E	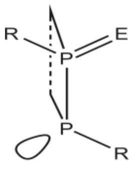	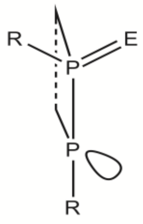 **TS-2**	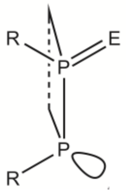	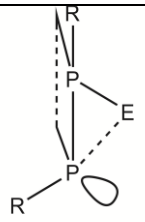 **TS-3**	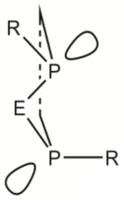	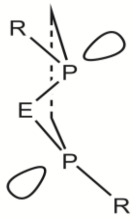 **TS-4**	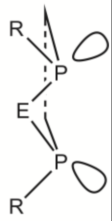
O	0.0	113.8	41.0	212.5	13.4	203.3	11.3
S	0.0	120.9	41.4	190.8	10.9	146.4	0.8

Se	0.0	124.3	42.7	179.5	2.1	138.1	-7.5
Te	0.0	123.8	42.3	191.2	-3.3	120.1	-12.1

Table S5 Relative energies of the investigated species at BP86/6-311+G** level of theory and in kJ/mol unit.

E	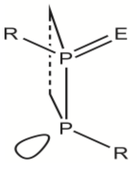	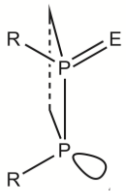	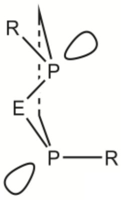	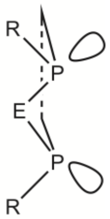
O	0.0	39.7	12.6	7.5
S	0.0	40.6	-0.4	-12.1
Se	0.0	41.4	-11.3	-22.6
Te	0.0	40.6	-19.2	-30.5

Table S6 Relative energies of the investigated species at M06-2X/6-311+G** level of theory and in kJ/mol unit.

E	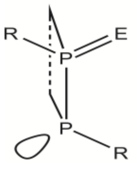	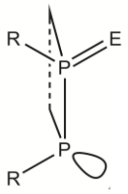	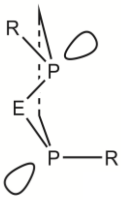	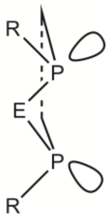
O	0.0	45.2	-10.5	-10.9
S	0.0	46.4	-1.3	-13.4
Se	0.0	47.3	-7.9	-20.5
Te	0.0	46.4	-12.1	-24.3

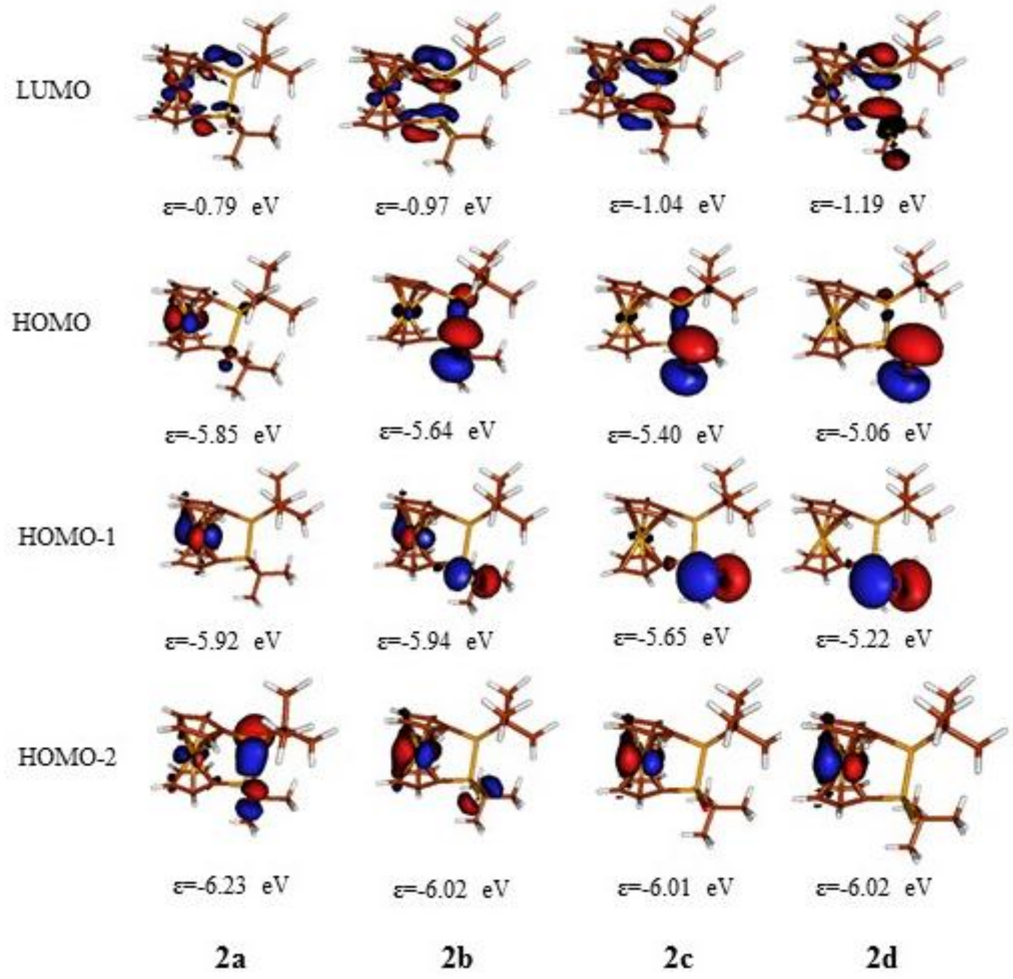

Figure S6. Kohn-Sham molecular orbitals of **2a**, **2b**, **2c**, **2d** at ωB97XD/6-311+G** level of theory.

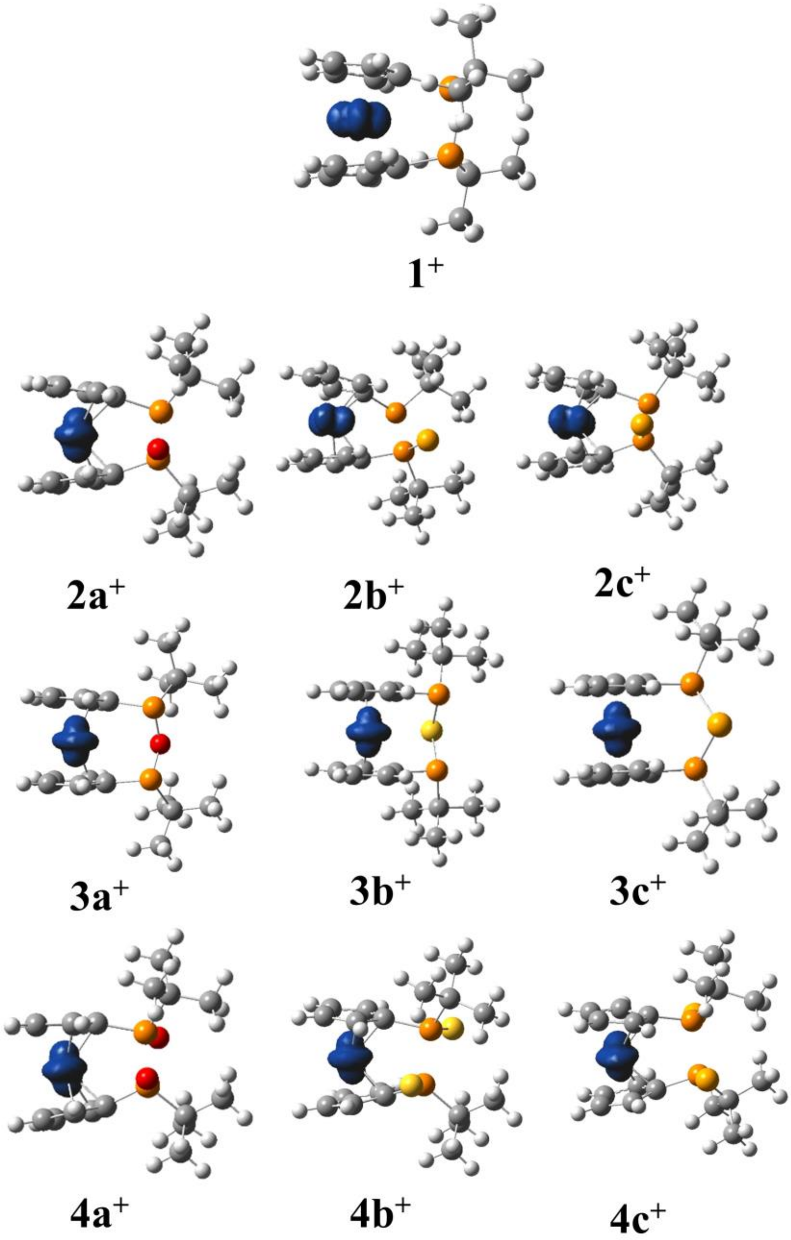

Figure S7. Spin density distribution of **1**$^+$, **2a,b,c**$^+$ **3a,b,c**$^+$ and **4a,b,c**$^+$ at B3LYP/6-311G**// ωB97XD/6-311+G**. It should be noted using other functionals the chalcogens have also some contribution to the spin density in case of the selenium derivatives (**2c**$^+$ and **4c**$^+$). It was in agreement with the experimentally observed irreversible redox response and indicates that the B3LYP does not describe properly the spin density (and the energy of the orbitals) in case of the selenium compounds.

Investigating bimolecular mechanisms:

One possible mechanism was depicted in **Scheme S2**. It contains one further reaction step (formation of **4b**,**c**), if we compare to the mechanism in Scheme 6. The disproportion of **2b**,**c** has $\Delta G^{\#}$=69.0-107.9 kJ/mol reaction barrier, which is in agreement with the experimentally obtained 83.0 kJ/mol. The next step is the transformation of **4b,c**→**5b,c**, which step has high barrier $\Delta E^{\#}$=163.2-175.3 kJ/mol. The formed **5** reacts with **1** giving the product **3b**,**c** and the starting **2b**,**c**. This mechanism was supported by the following data:

- We could not localise a transition state for the direct formation of **5b,c** from **2b,c**. This mechanism solves this problem.
- This mechanism contains bimolecular transitions states, which energy levels are comparable to the experimentally obtained values.
- Somewhat lower barrier has been computed for the reaction **4**→**5**, than for the direct transformation of **2**→**3**.

On the other hand, several data are against this process, which are the followings:

- There is no experimental evidence for the formation of **4**.
- According to the experiments the reaction **4c**→**5c** took place at higher temperature than **2c**→**3c**.
- According to the calculations the rate limiting step is **4**→**5**, which is a monomolecular reaction. Both the high barrier and the molecularity of this step are against the experimentally obtained values.

This later problem can be solved if photoactivation was considered. Investigating the UV-vis spectra of **4c** it can be established that this compound absorbs visible light and TD-DFT calculations reveals that many of the excitation contains n(selenium)-σ*(P-P bond) transitions, which result in elongation of the P-P bond. Despite the promising DFT results this hypothesis was not verified experimentally.

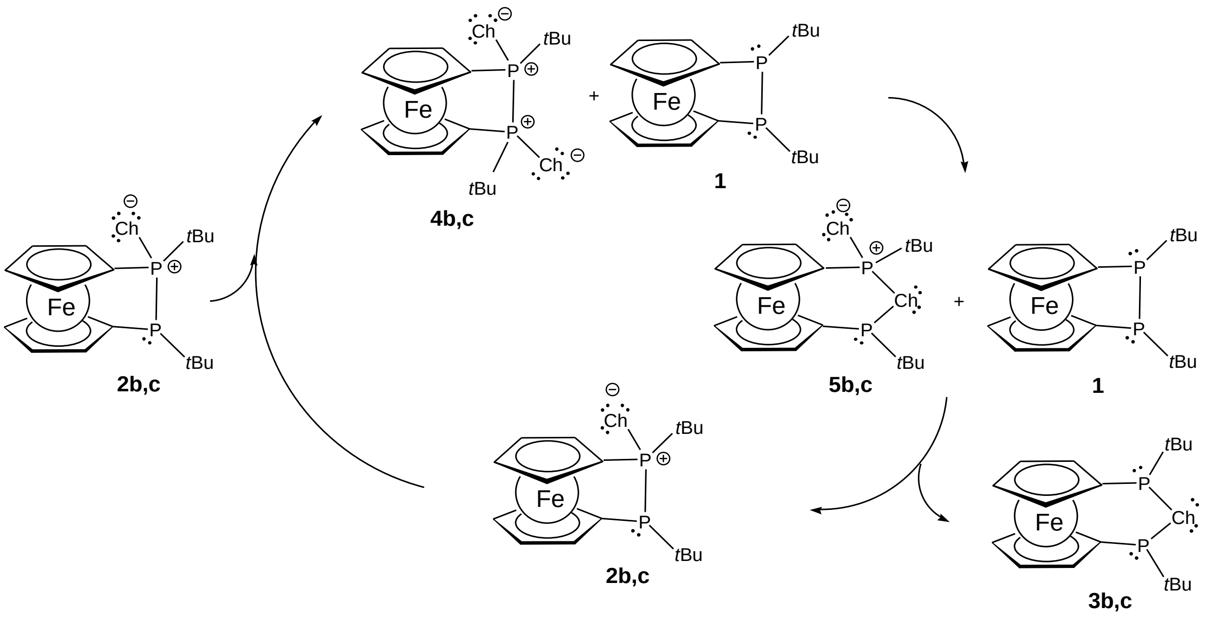

Scheme S2. One possible mechanism containing **4b,c.**

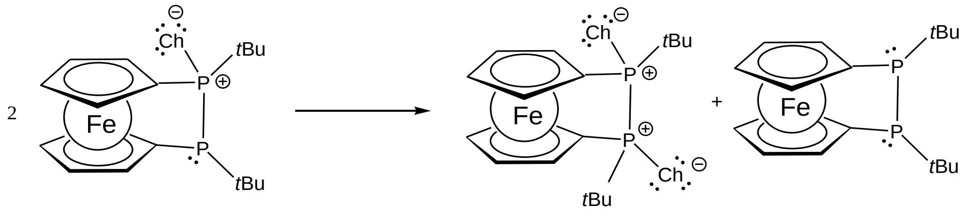

2b,c **4b,c** **1**

Ch	ΔG#	ΔG
S	107.9	7.1
Se	69.0	19.7

Scheme S3. Reaction Gibbs free energy of the disproportion of **2** at ωB97XD/6-311G** in kJ/mol unit

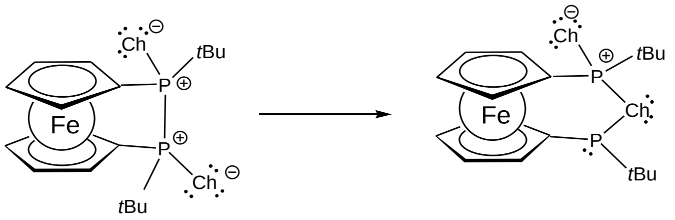

4a,b,c **5a,b,c**

Ch	ΔE#	ΔE
O	175.3	-42.3
S	176.6	-2.9
Se	163.2	-9.6

Scheme S4. Reaction barrier of the **4**→**5** transformation at ωB97XD/6-311+G** level of theory in kJ/mol unit

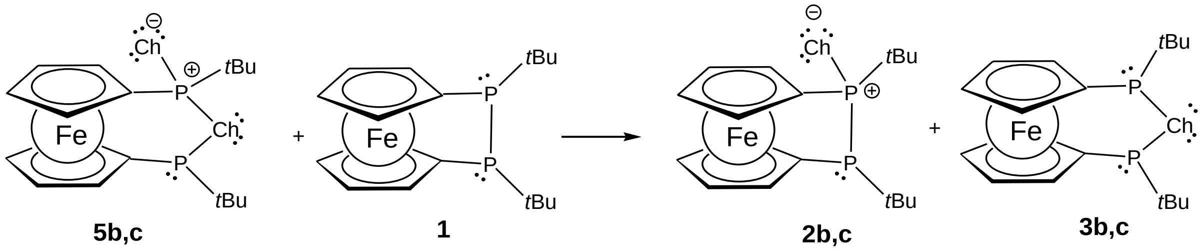

5b,c **1** **2b,c** **3b,c**

Ch	ΔG#	ΔG
S	83.7	-9.6
Se	57.7	-39.7

Scheme S5. Reaction Gibbs free energy of the synproportion of **5** and **1** at ωB97XD/6-311G** and in kJ/mol unit

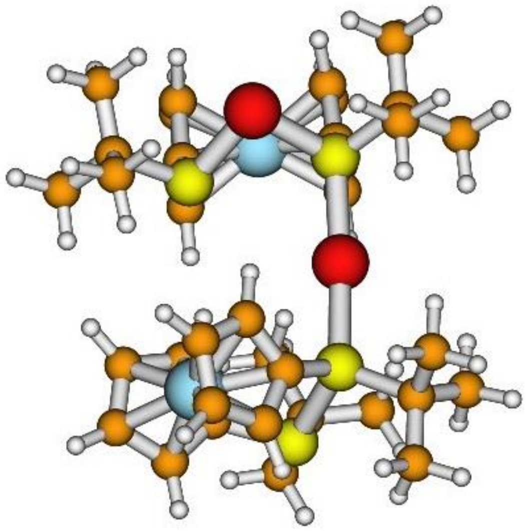

Figure S8 Transition state of the chalcogen transfer between **1** and **4c**

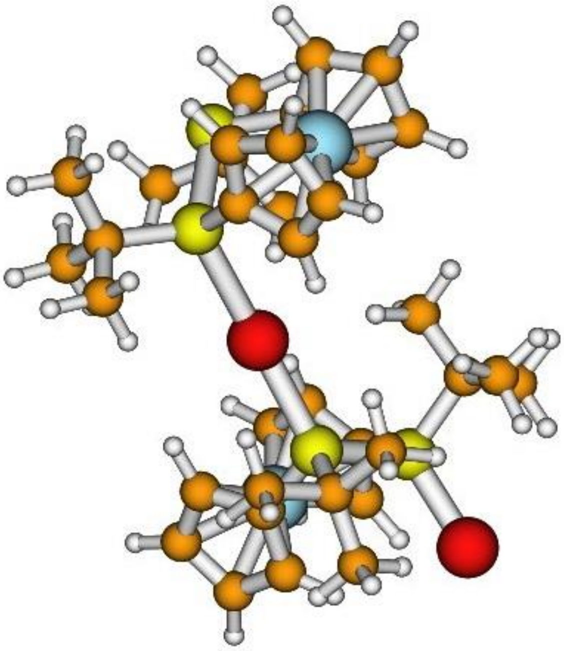

Figure S9. Transition state of the chalcogen transfer between two **2c** (disproportion of **2c**)

Kinetic investigations

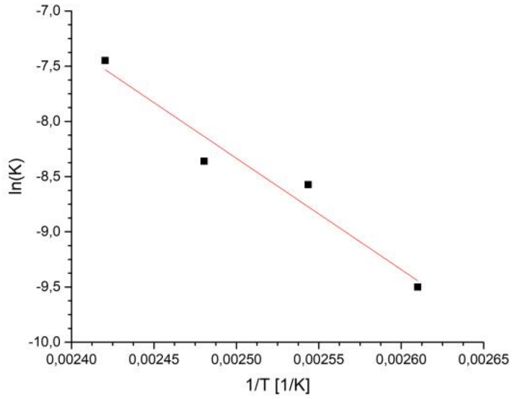

Figure S10. ln(K) over 1/T plot from the evaluation as a second order reaction (without exclusion of light).

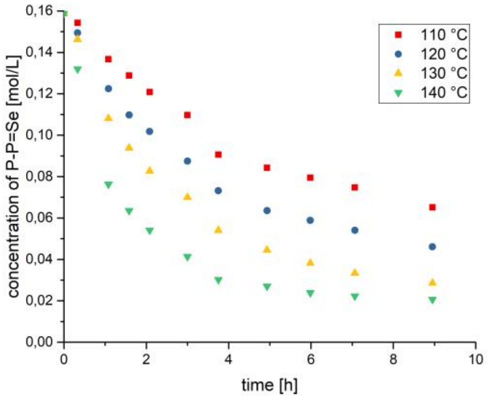

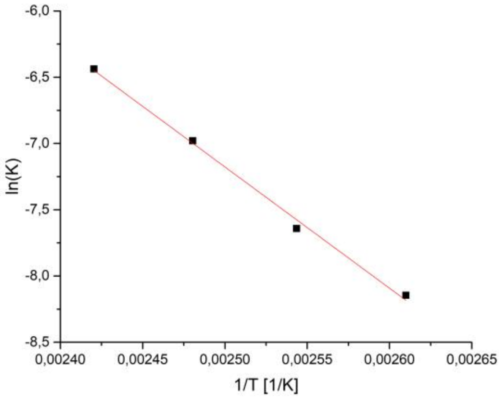

Figure S11. Temperature dependence of the rearrangement **2c**→**3c** (under exclusion of light, brown glass NMR-tube) over time with a starting concentration c_0(**2c**) = 0.159 mol/L (left). ln(K) over 1/T plot from the evaluation as a second order reaction (right).

On the following pages the NMR-spectra of compounds **2**, **2a**, **2b**, **2c**, **3b**, **3c, 3d**, **4a, 4b**, **4c, 5a, 5b** and **5c** are depicted:

NMR-spectra of **2**:

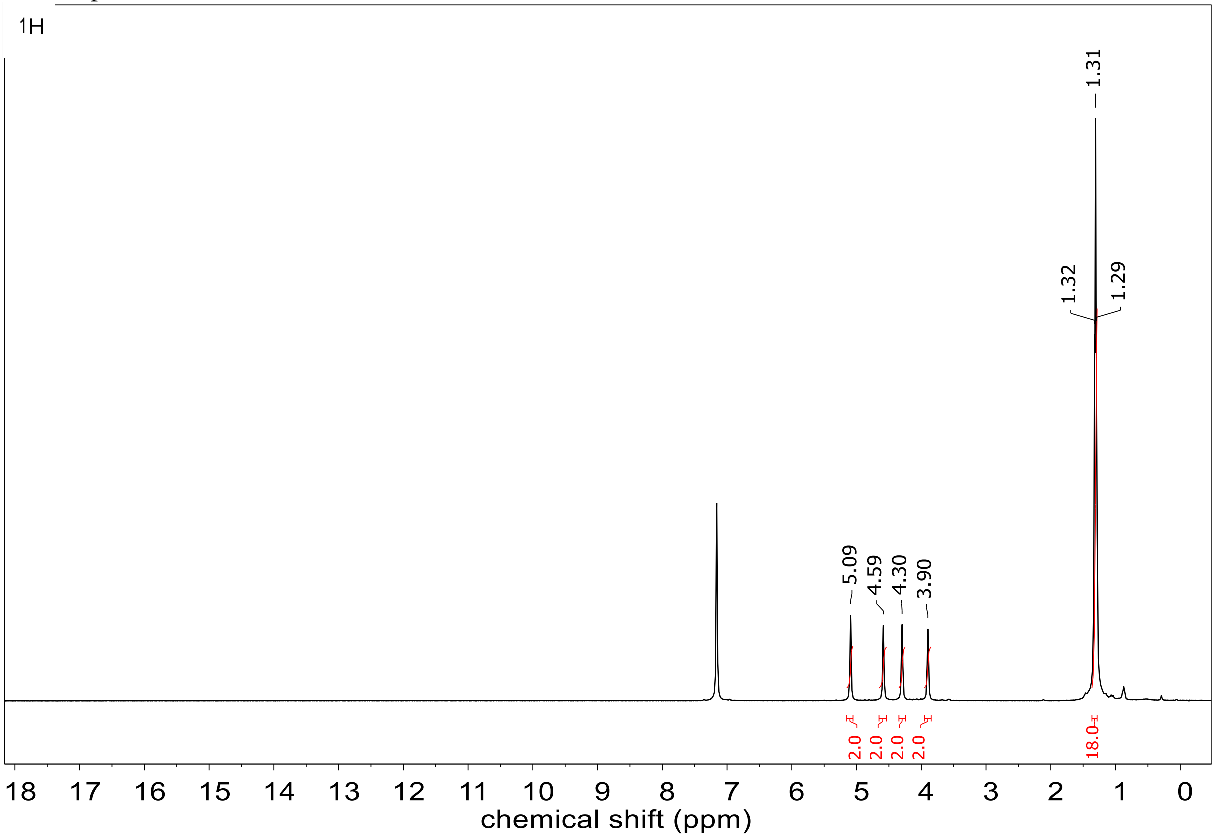

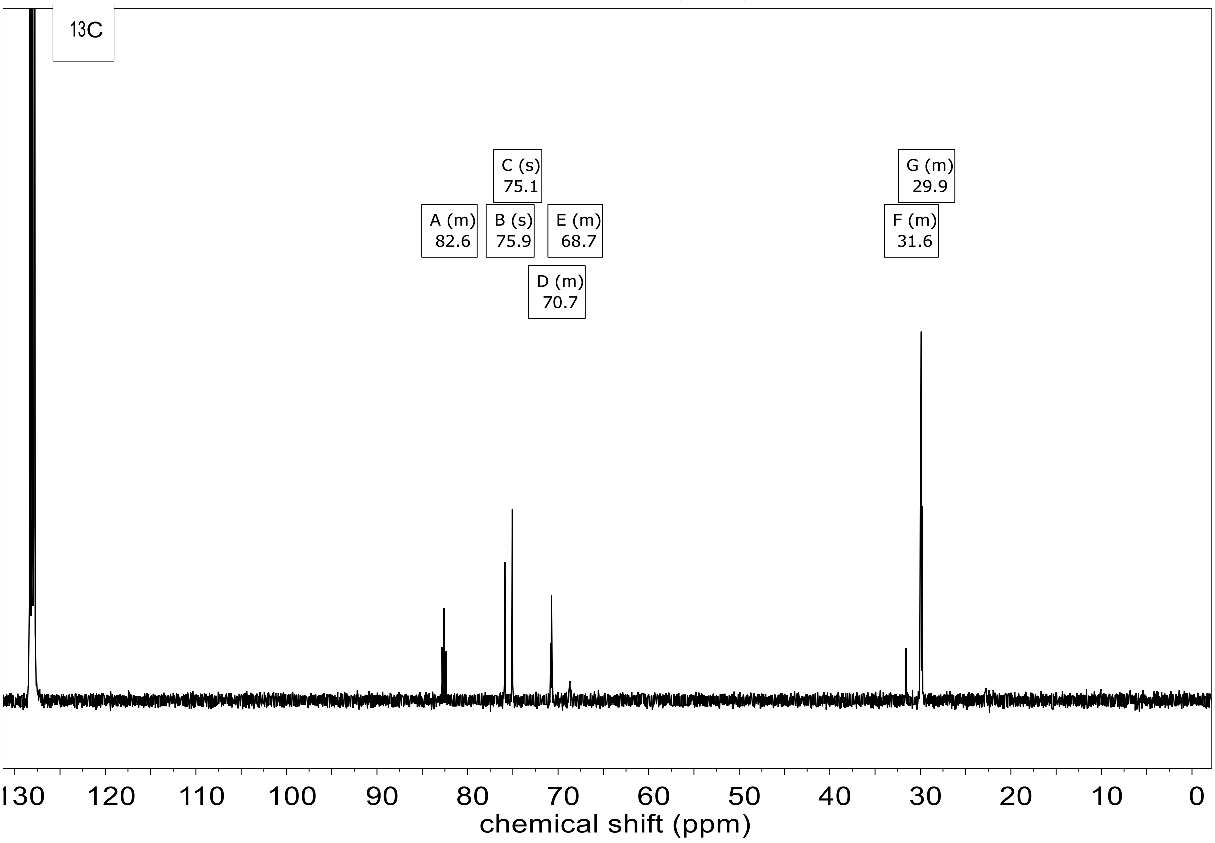

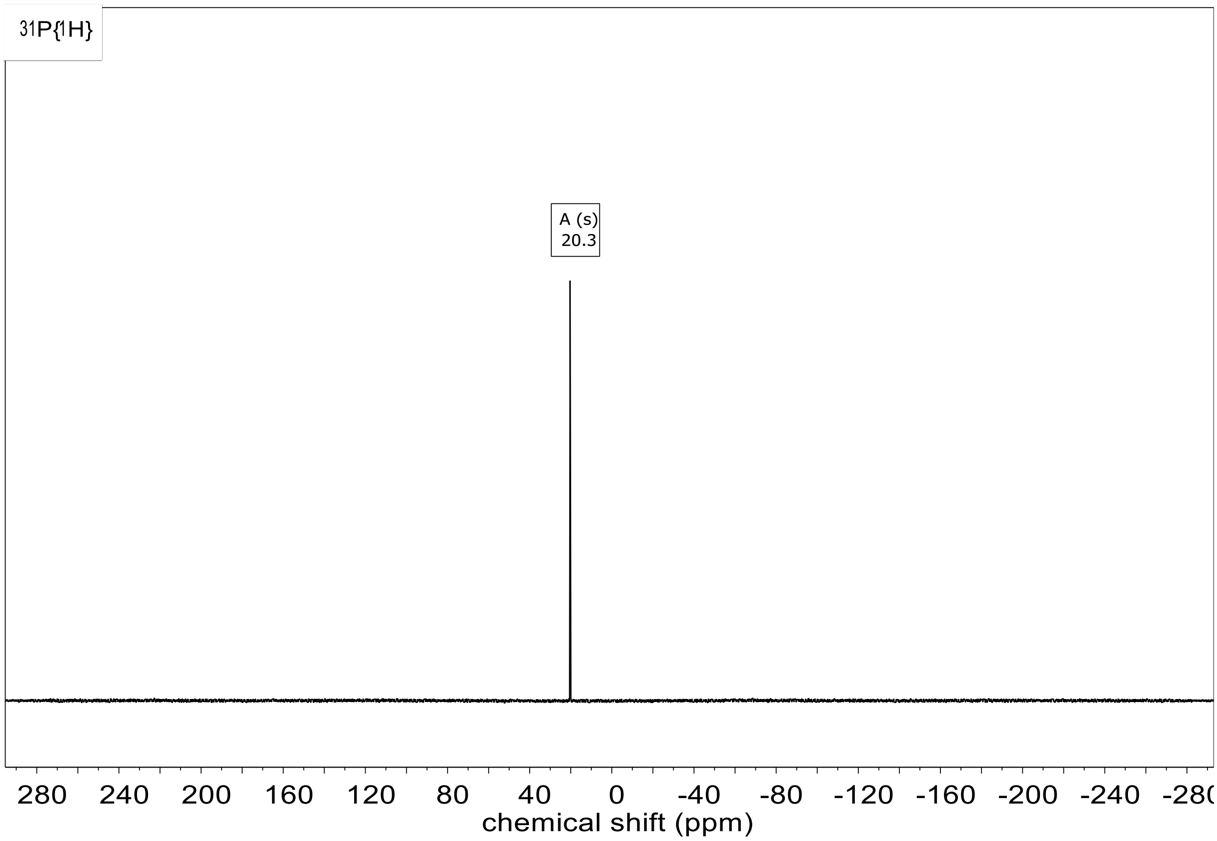

NMR-spectra of **2a**:

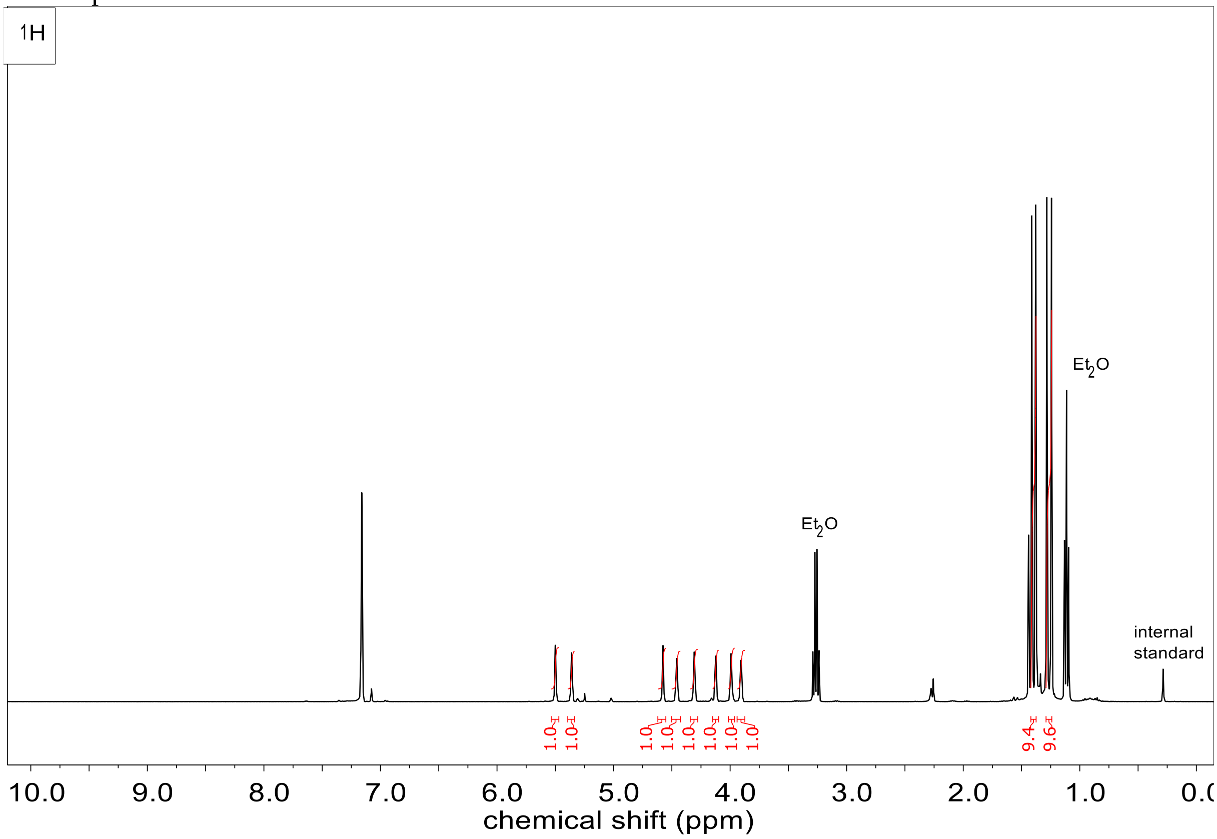

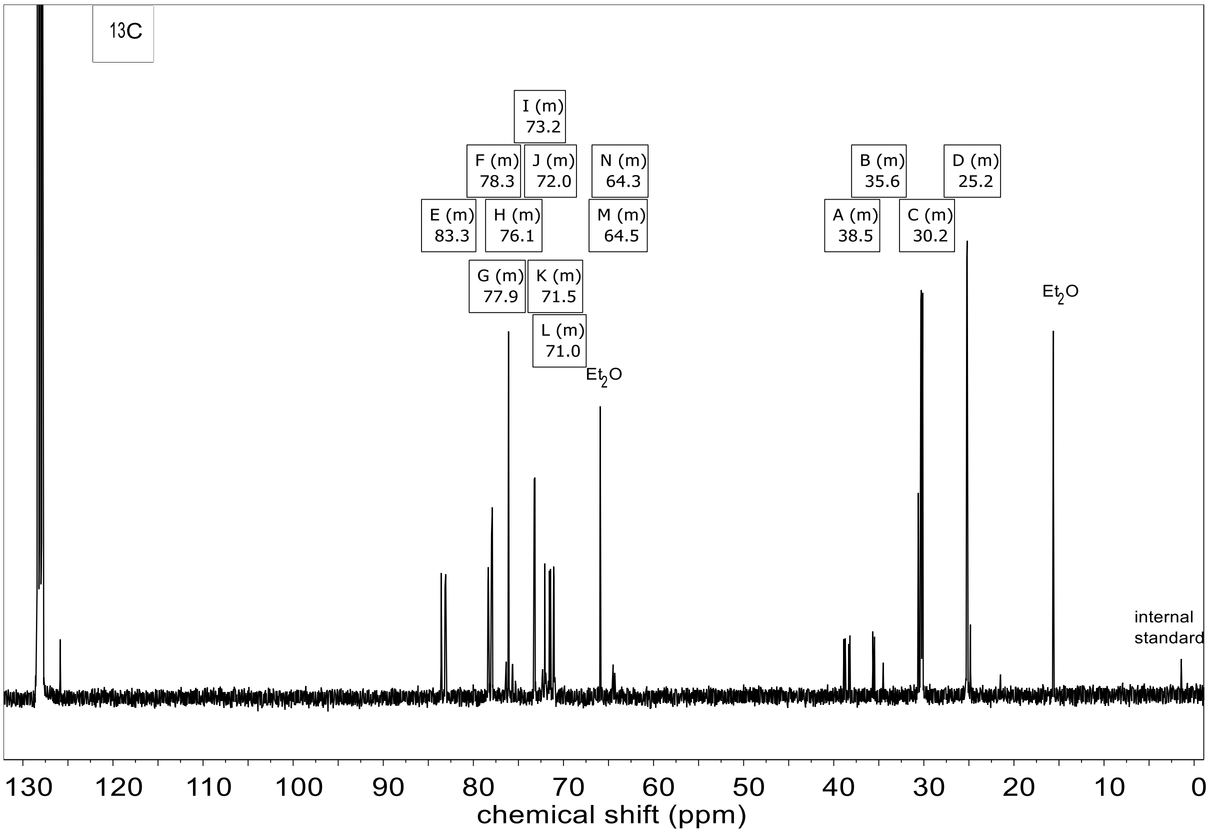
13C
I (m)
73.2
F (m)
78.3
J (m)
72.0
N (m)
64.3
B (m)
35.6
D (m)
25.2
E (m)
83.3
H (m)
76.1
M (m)
64.5
A (m)
38.5
C (m)
30.2
G (m)
77.9
K (m)
71.5
L (m)
71.0
Et2O
Et2O
internal standard
130 120 110 100 90 80 70 60 50 40 30 20 10 0
chemical shift (ppm)

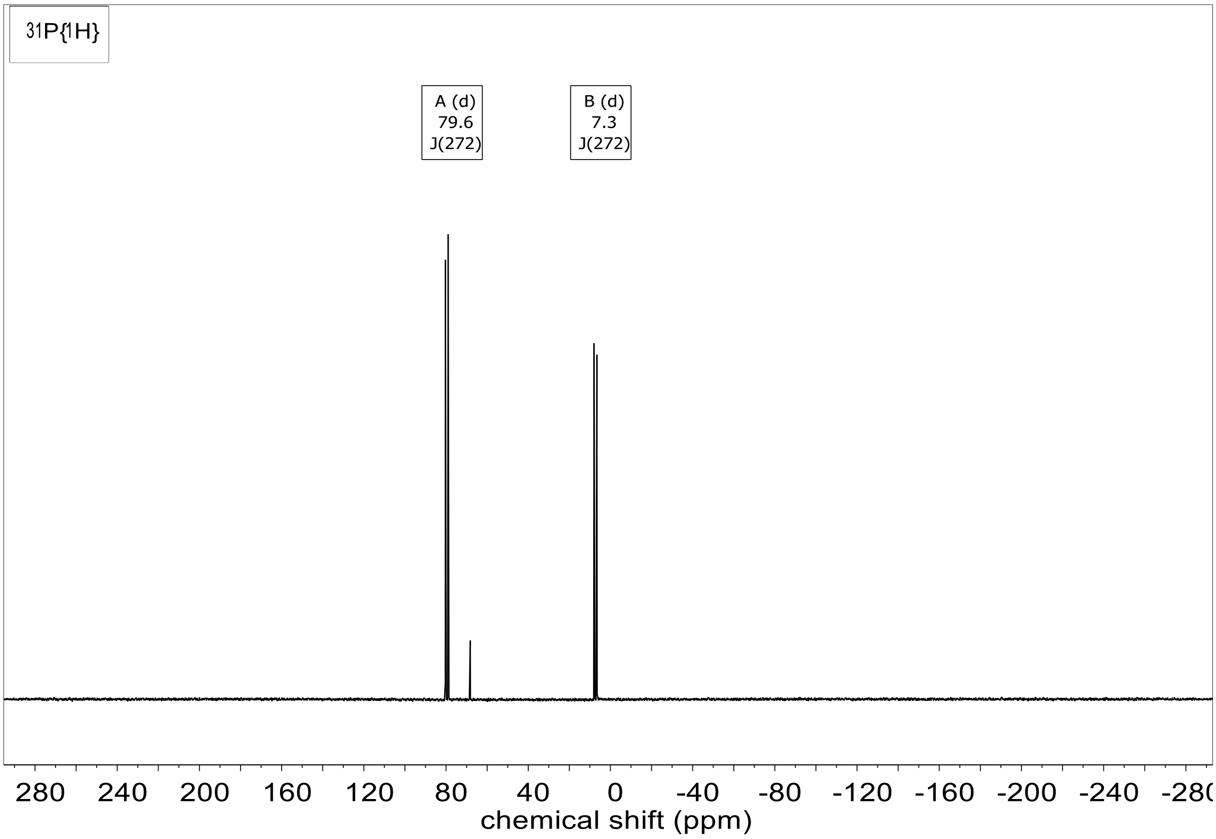
31P{1H}
A (d)
79.6
J(272)
B (d)
7.3
J(272)
280 240 200 160 120 80 40 0 -40 -80 -120 -160 -200 -240 -280
chemical shift (ppm)

NMR-spectra of **2b**:

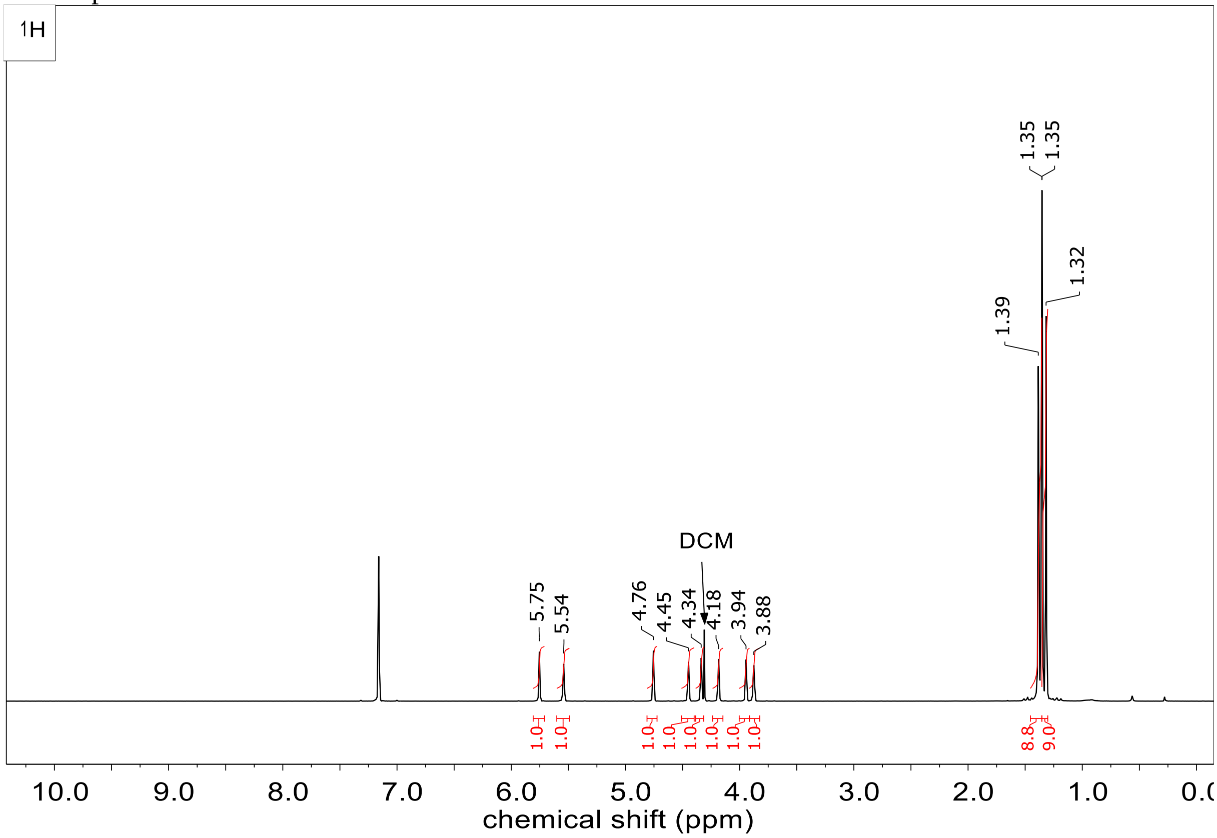

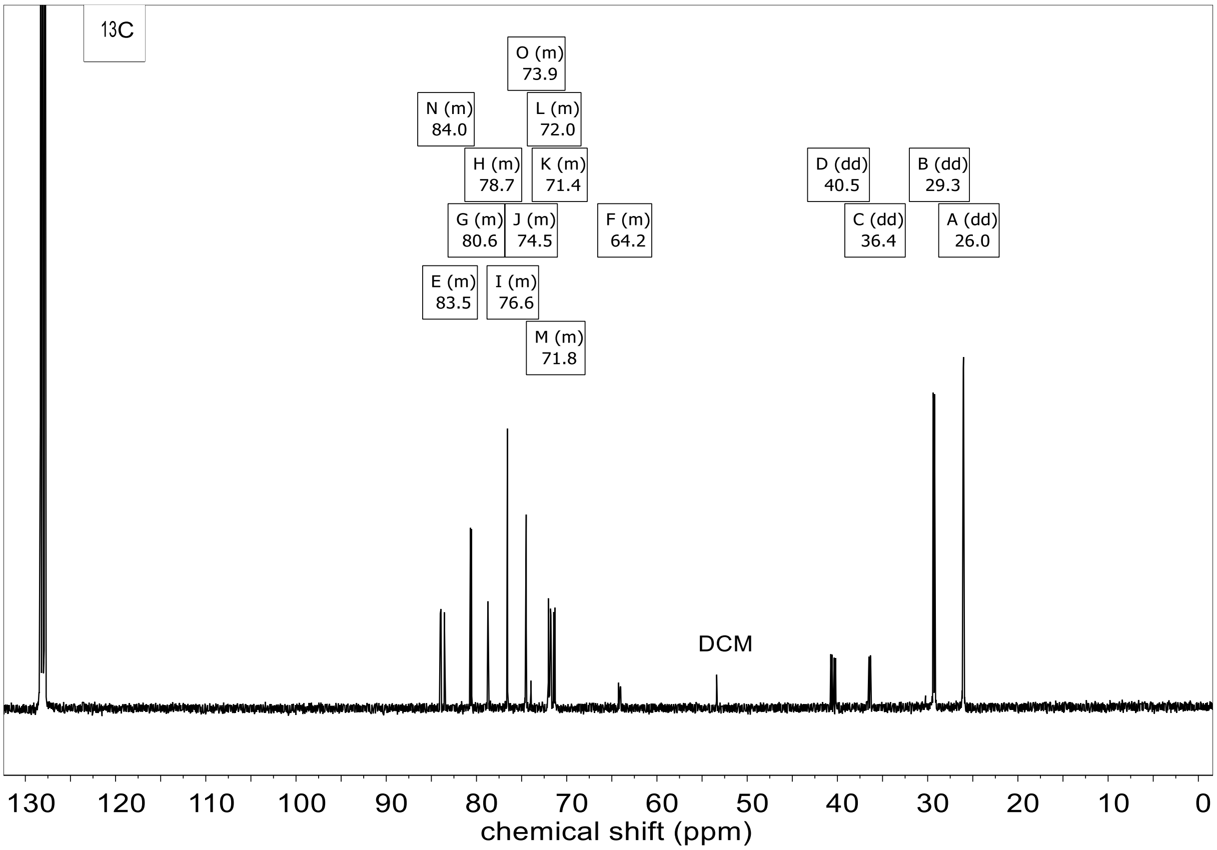

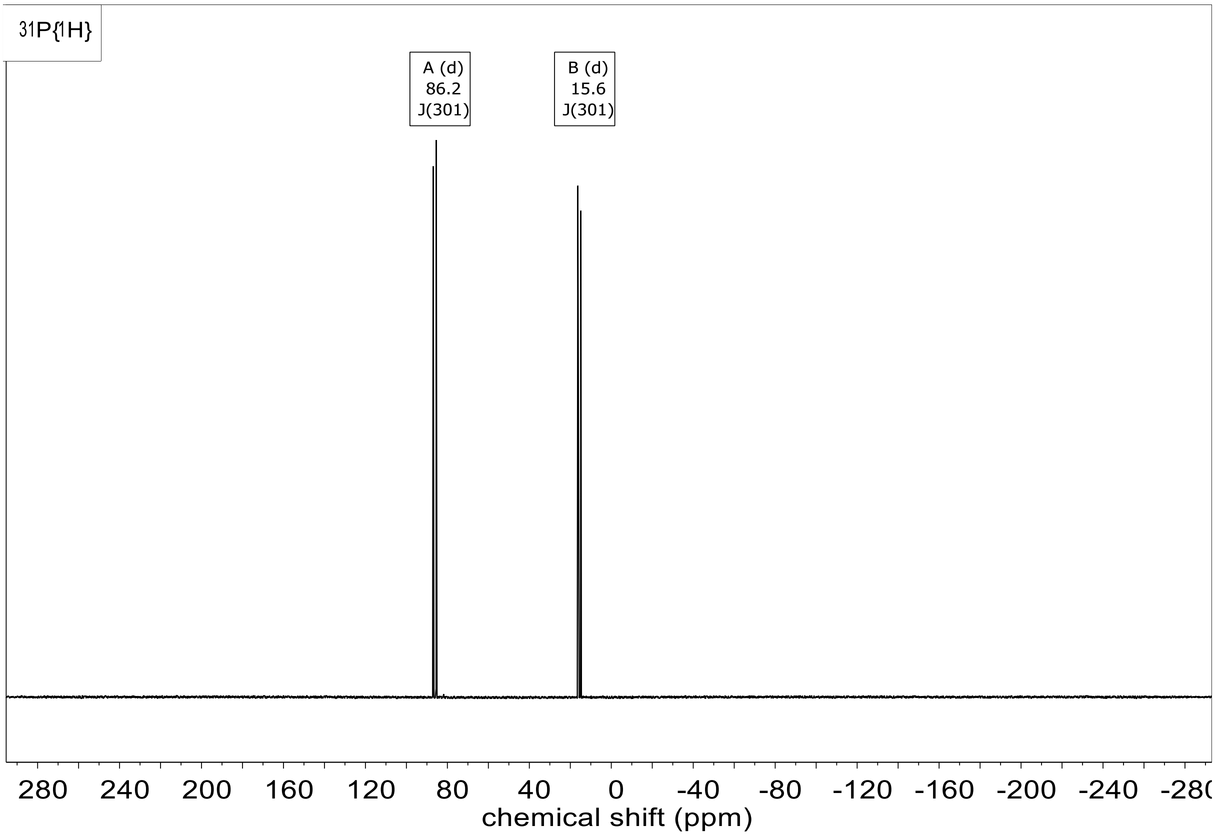

NMR-spectra of **2c**:

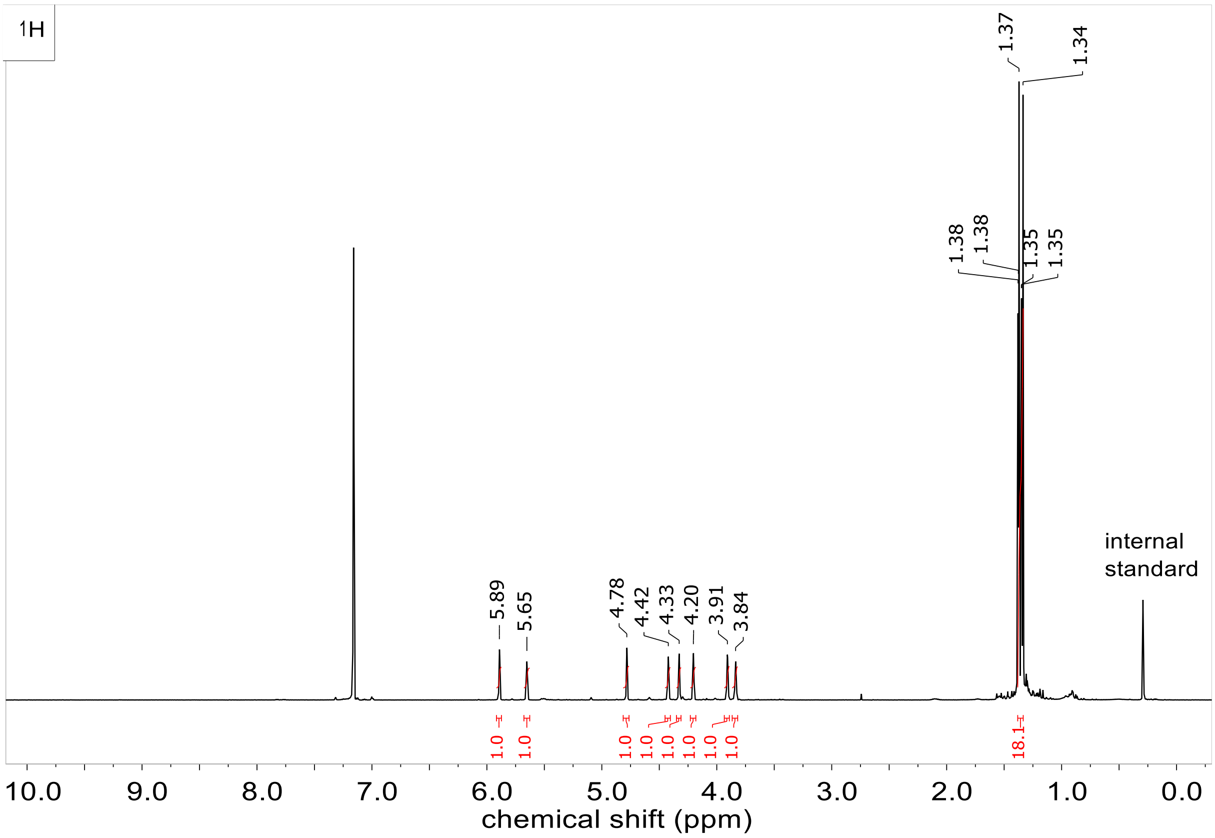

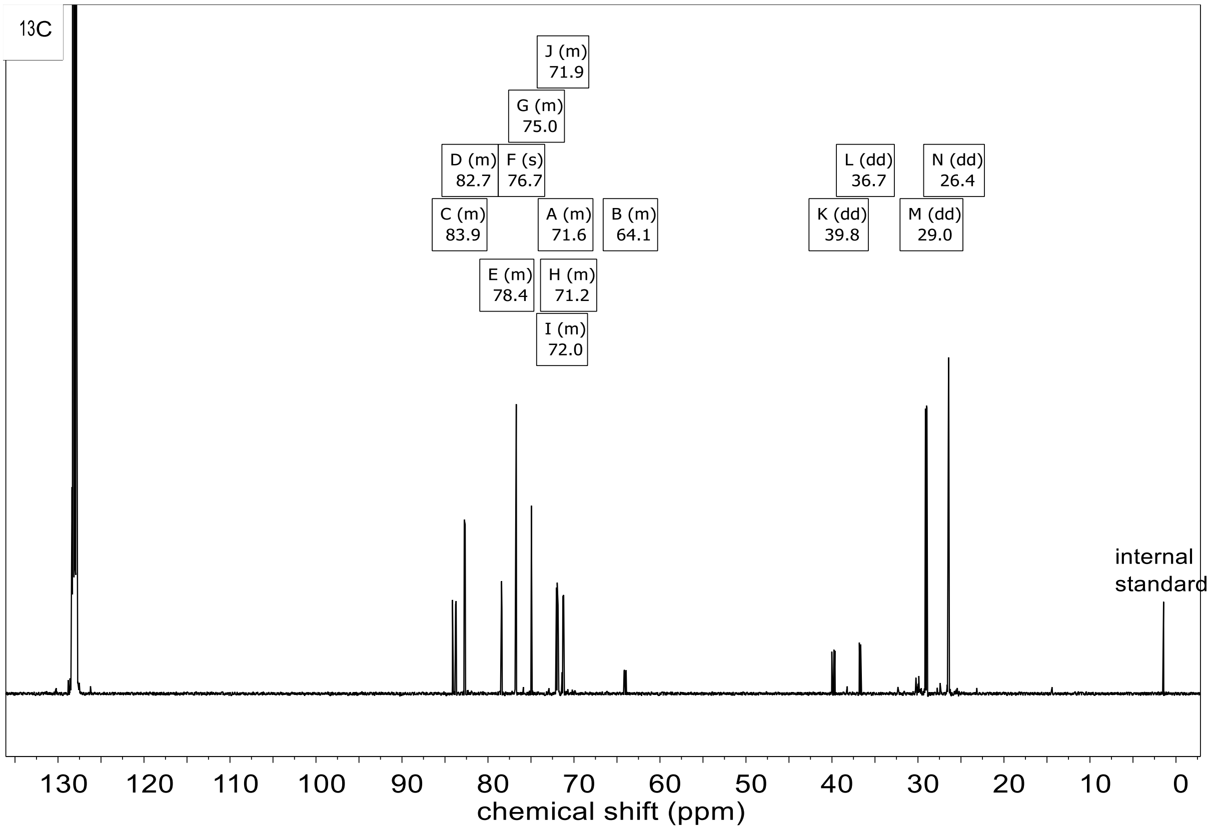
13C
J (m) 71.9
G (m) 75.0
D (m) 82.7
F (s) 76.7
L (dd) 36.7
N (dd) 26.4
C (m) 83.9
A (m) 71.6
B (m) 64.1
K (dd) 39.8
M (dd) 29.0
E (m) 78.4
H (m) 71.2
I (m) 72.0
internal standard
130 120 110 100 90 80 70 60 50 40 30 20 10 0
chemical shift (ppm)

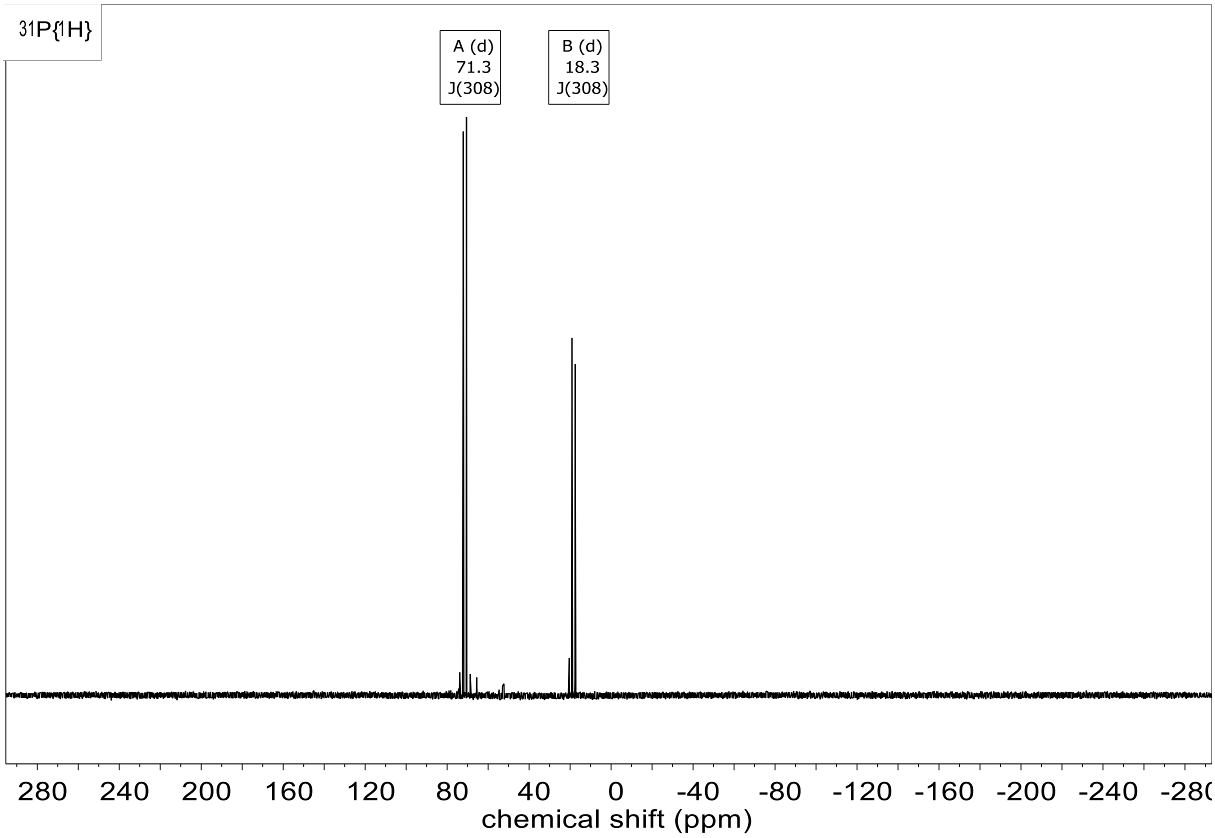
31P{1H}
A (d) 71.3 J(308)
B (d) 18.3 J(308)
280 240 200 160 120 80 40 0 -40 -80 -120 -160 -200 -240
chemical shift (ppm)

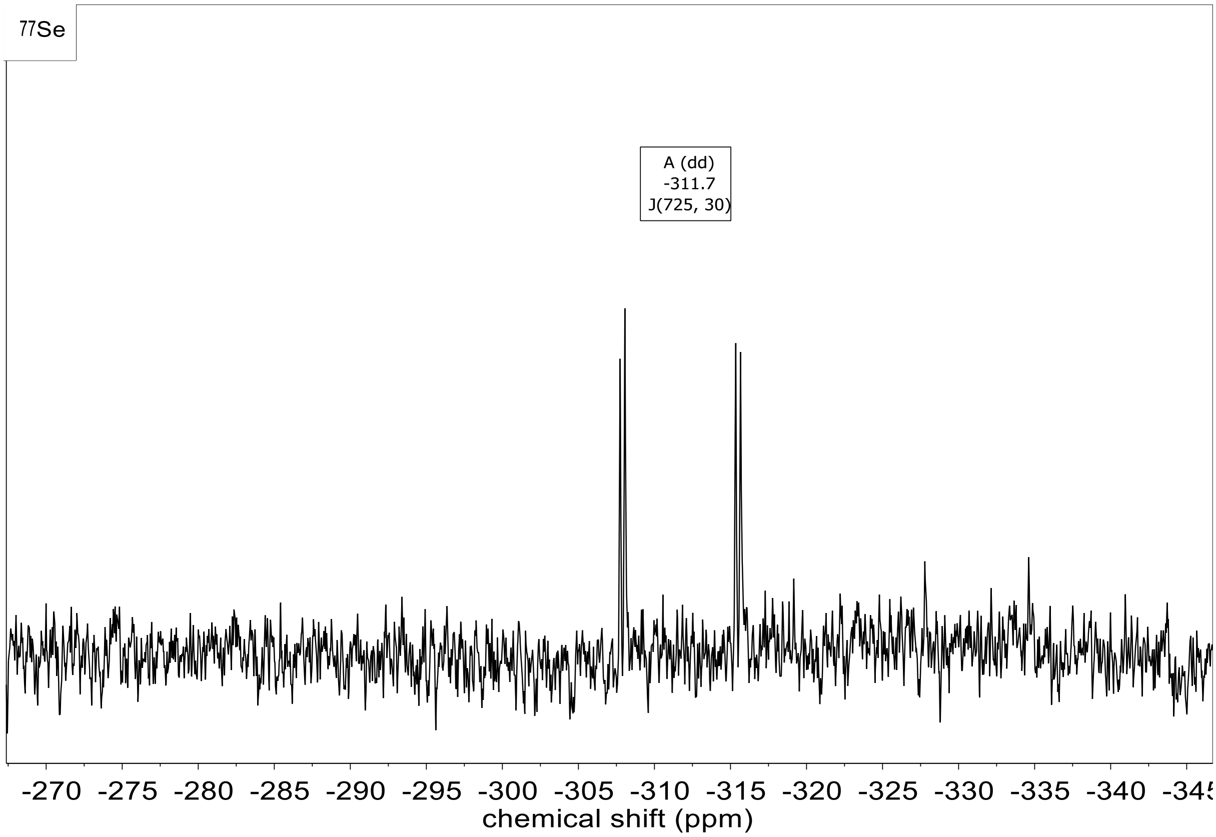

NMR-spectra of **3b**:

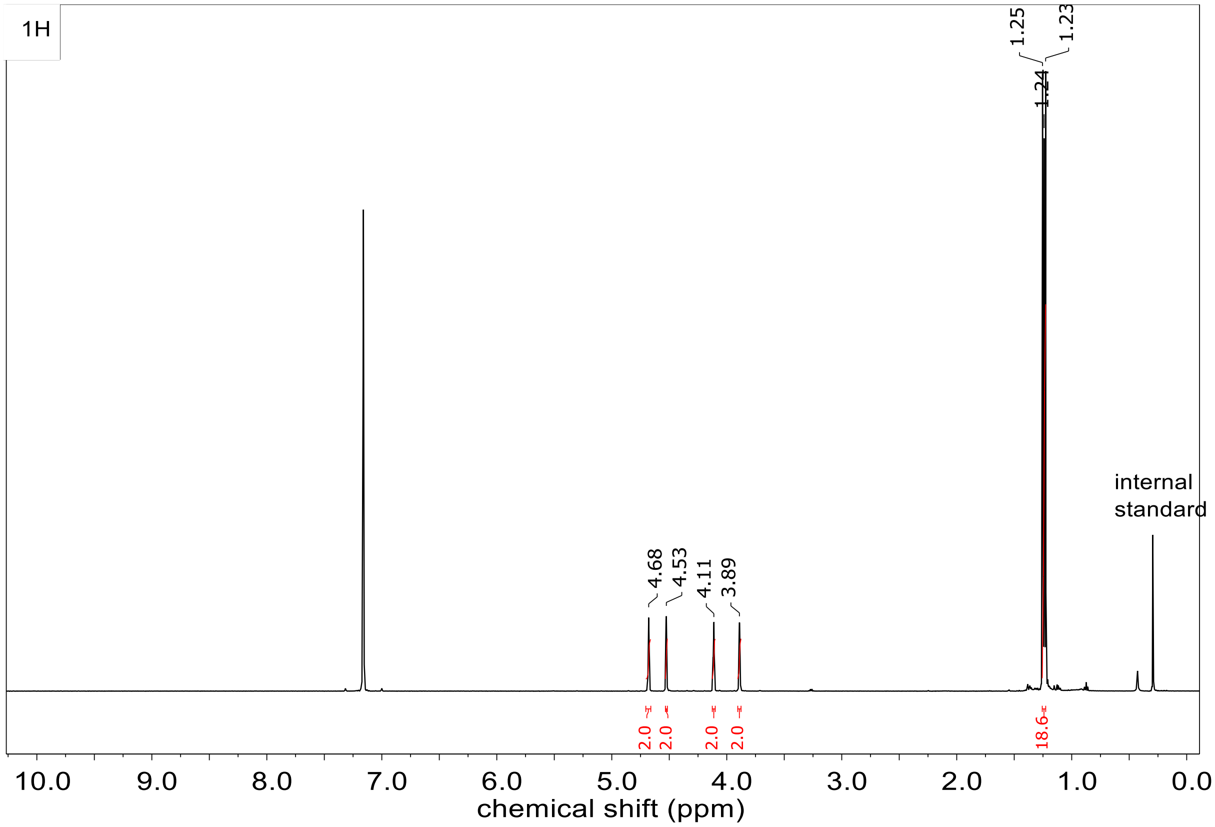

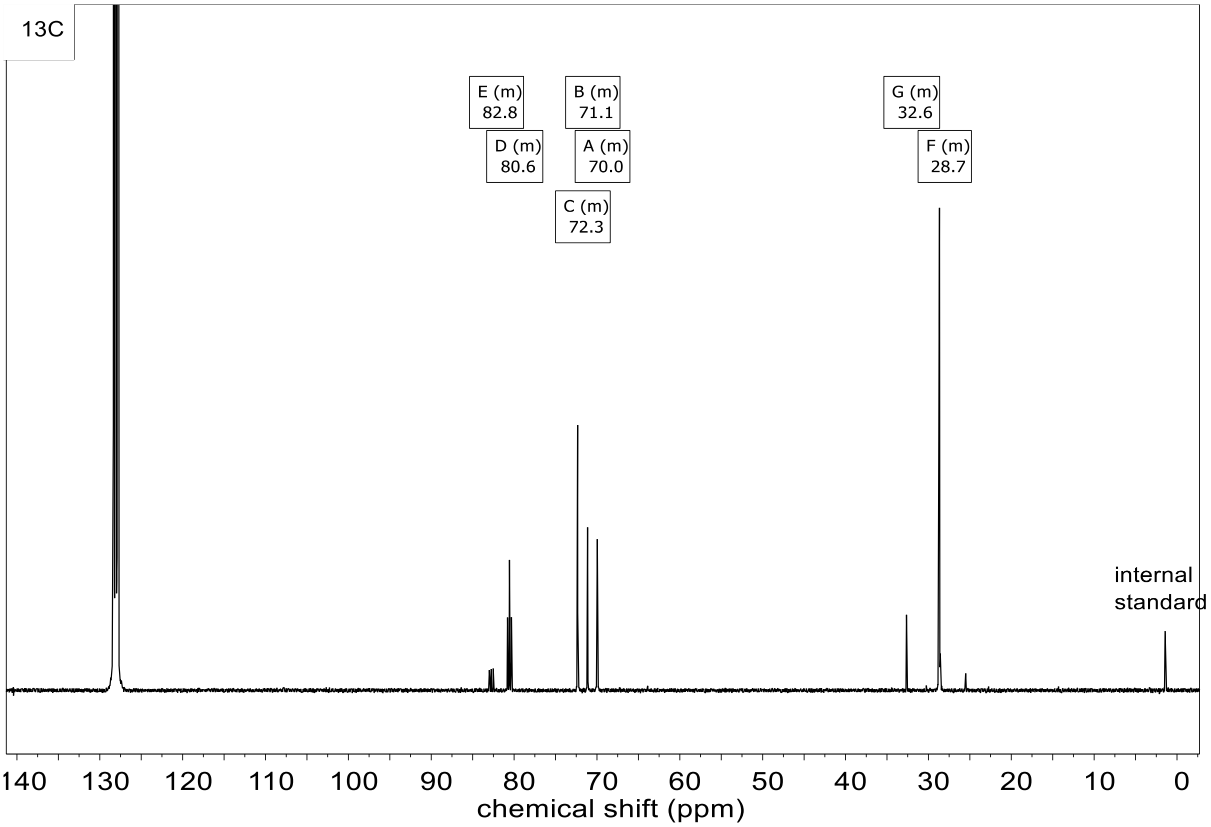
13C
E (m) 82.8
D (m) 80.6
B (m) 71.1
A (m) 70.0
C (m) 72.3
G (m) 32.6
F (m) 28.7
internal standard
140 130 120 110 100 90 80 70 60 50 40 30 20 10 0
chemical shift (ppm)

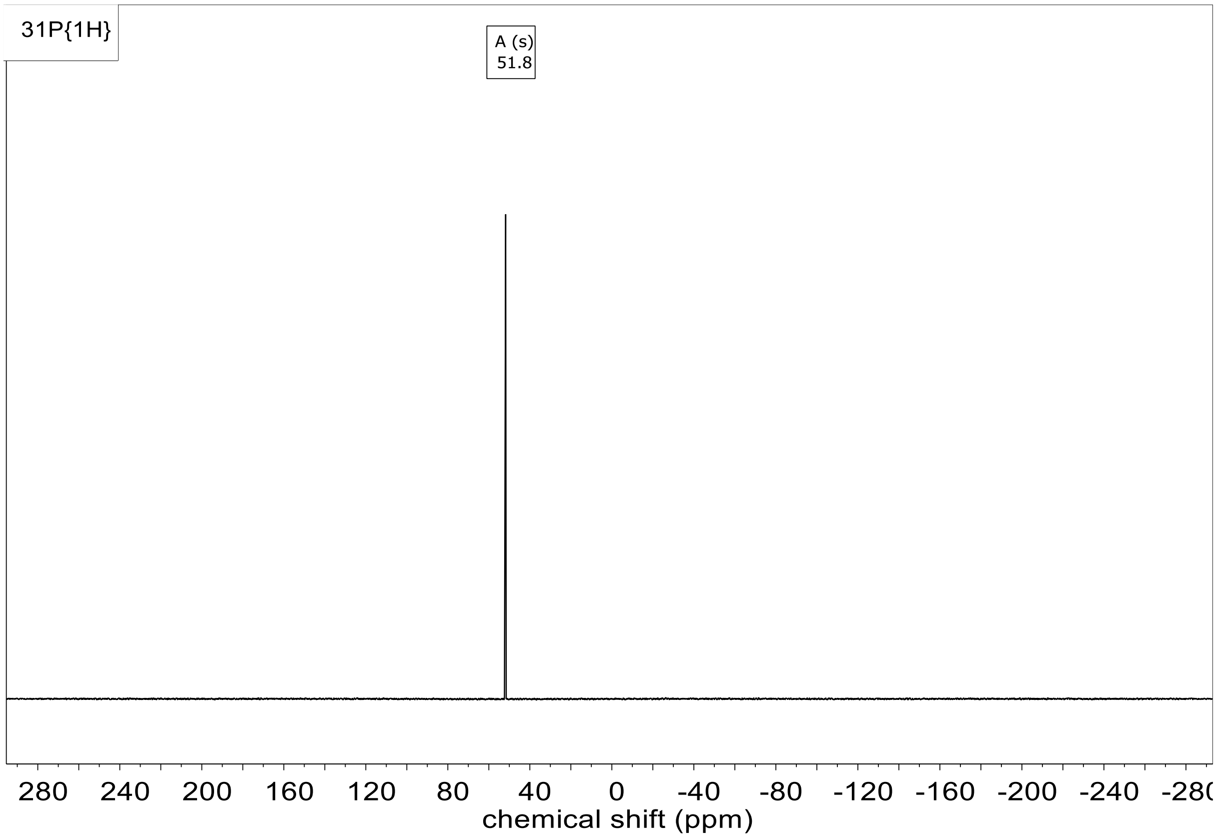
31P{1H}
A (s) 51.8
280 240 200 160 120 80 40 0 -40 -80 -120 -160 -200 -240
chemical shift (ppm)

NMR-spectra of **3c**:

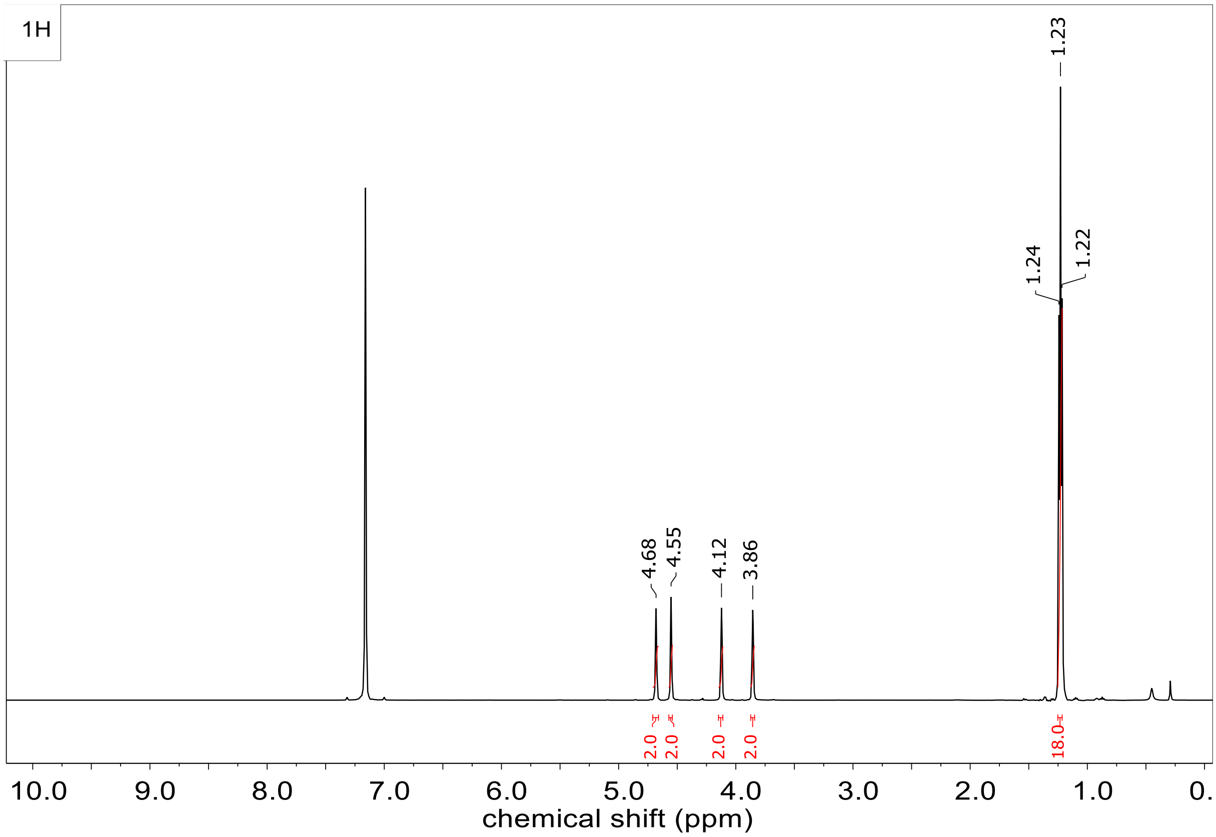

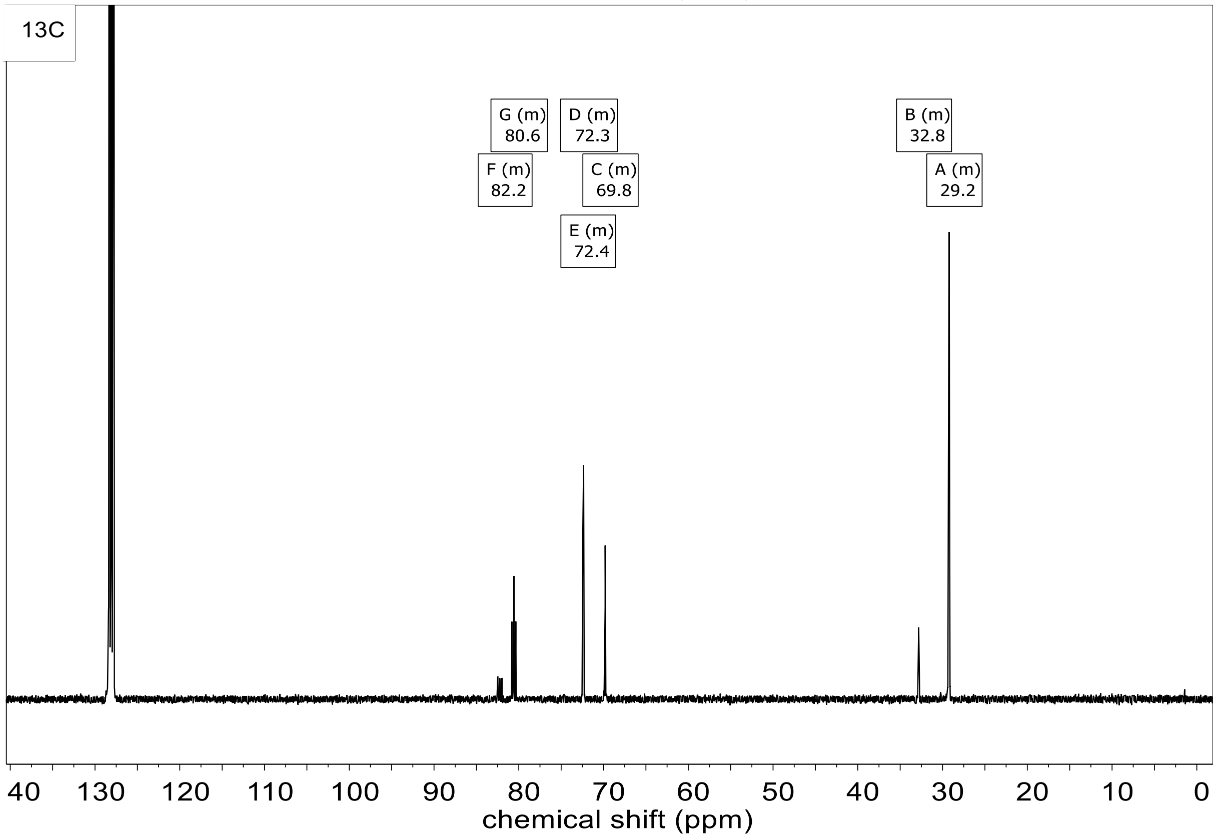

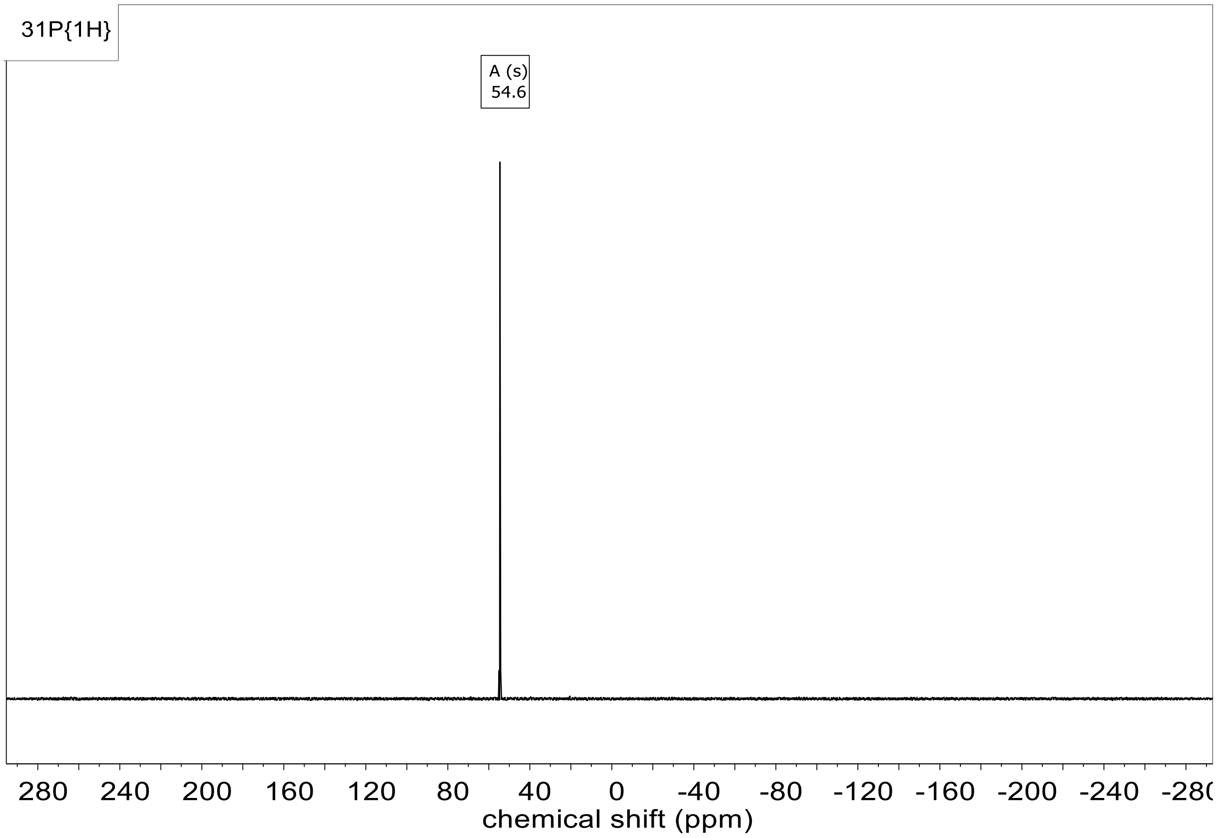
31P{1H}
A (s)
54.6
280 240 200 160 120 80 40 0 -40 -80 -120 -160 -200 -240 -280
chemical shift (ppm)

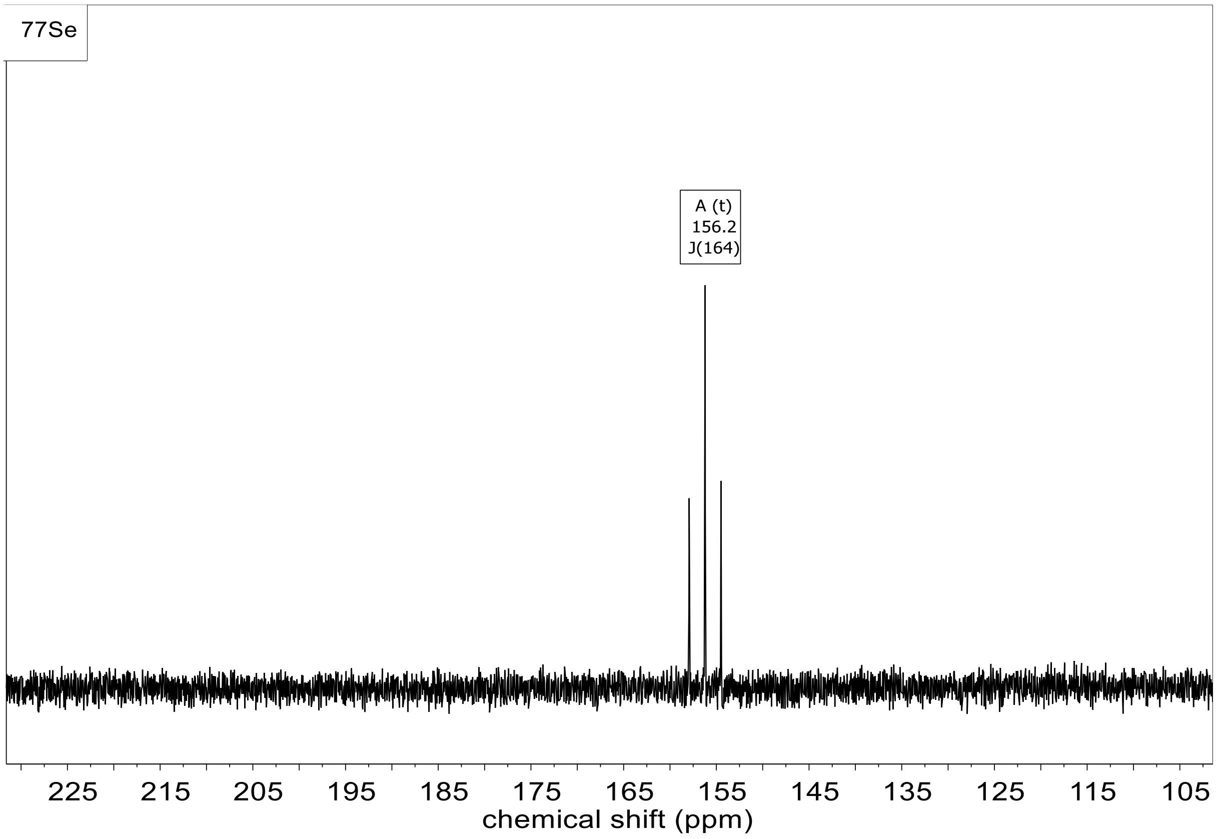
77Se
A (t)
156.2
J(164)
225 215 205 195 185 175 165 155 145 135 125 115 105
chemical shift (ppm)

NMR-spectra of **3d**:

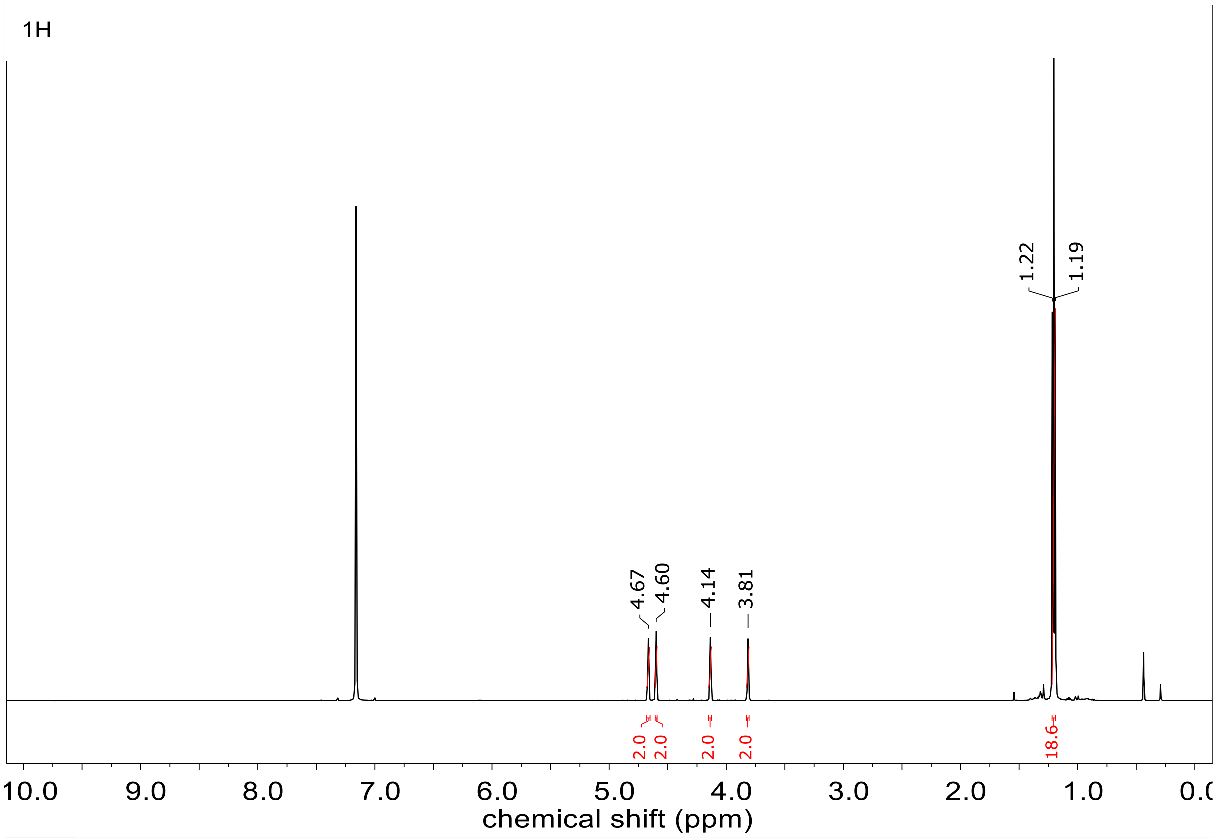

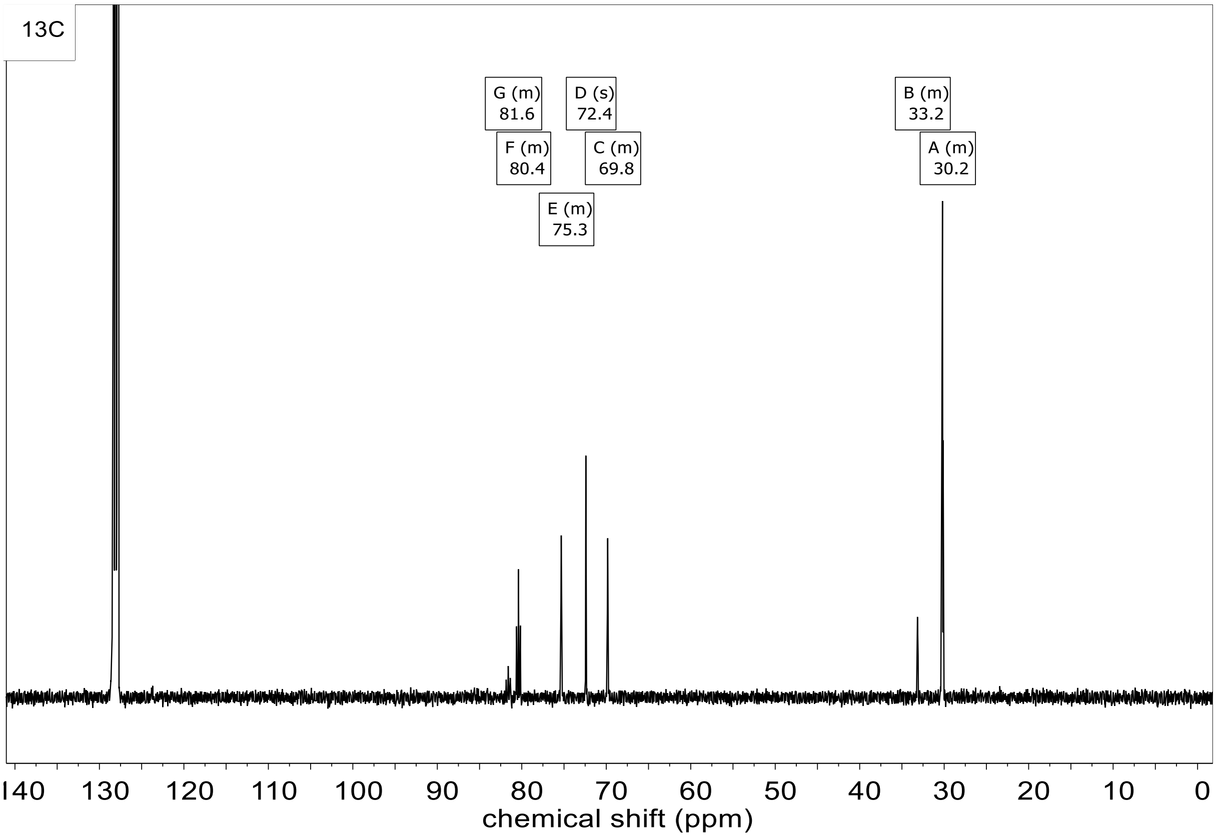

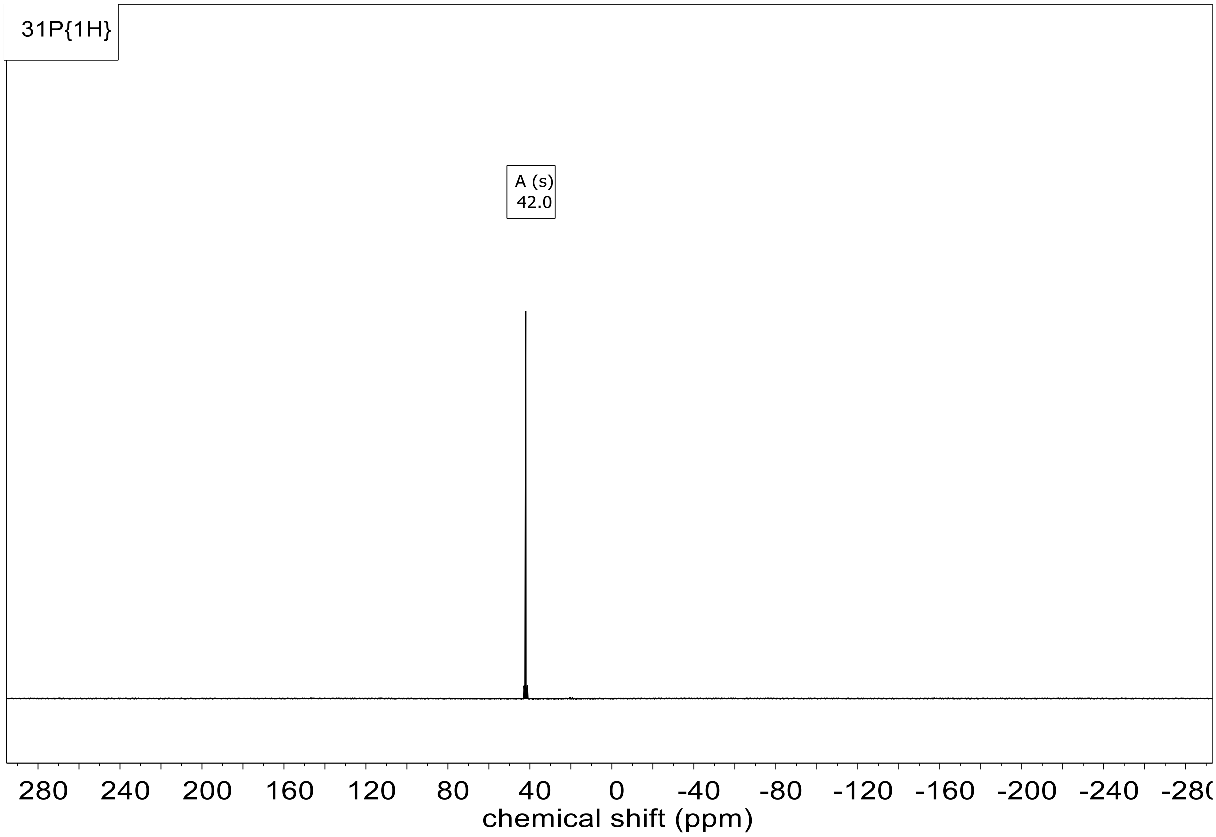
31P{1H}
A (s)
42.0
280 240 200 160 120 80 40 0 -40 -80 -120 -160 -200 -240
chemical shift (ppm)

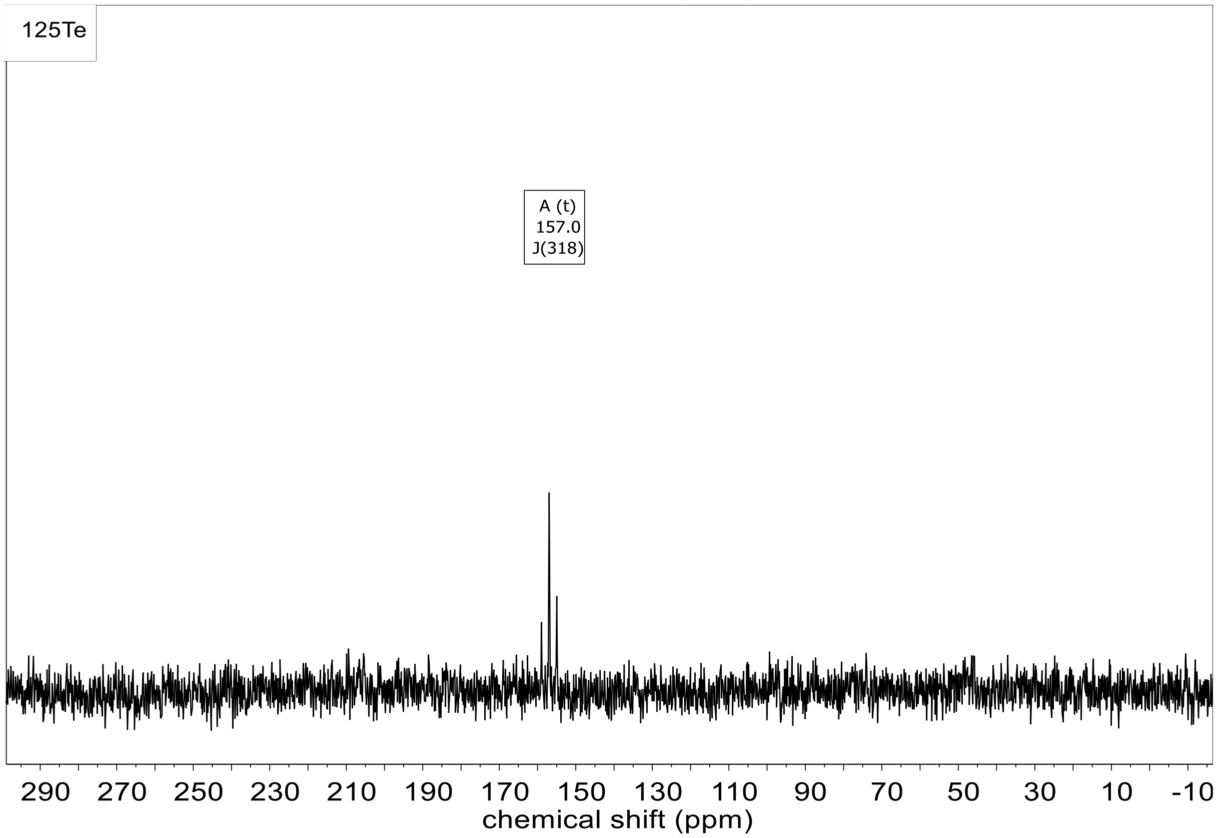
125Te
A (t)
157.0
J(318)
290 270 250 230 210 190 170 150 130 110 90 70 50 30 10 -10
chemical shift (ppm)

NMR-spectra of **4a**:

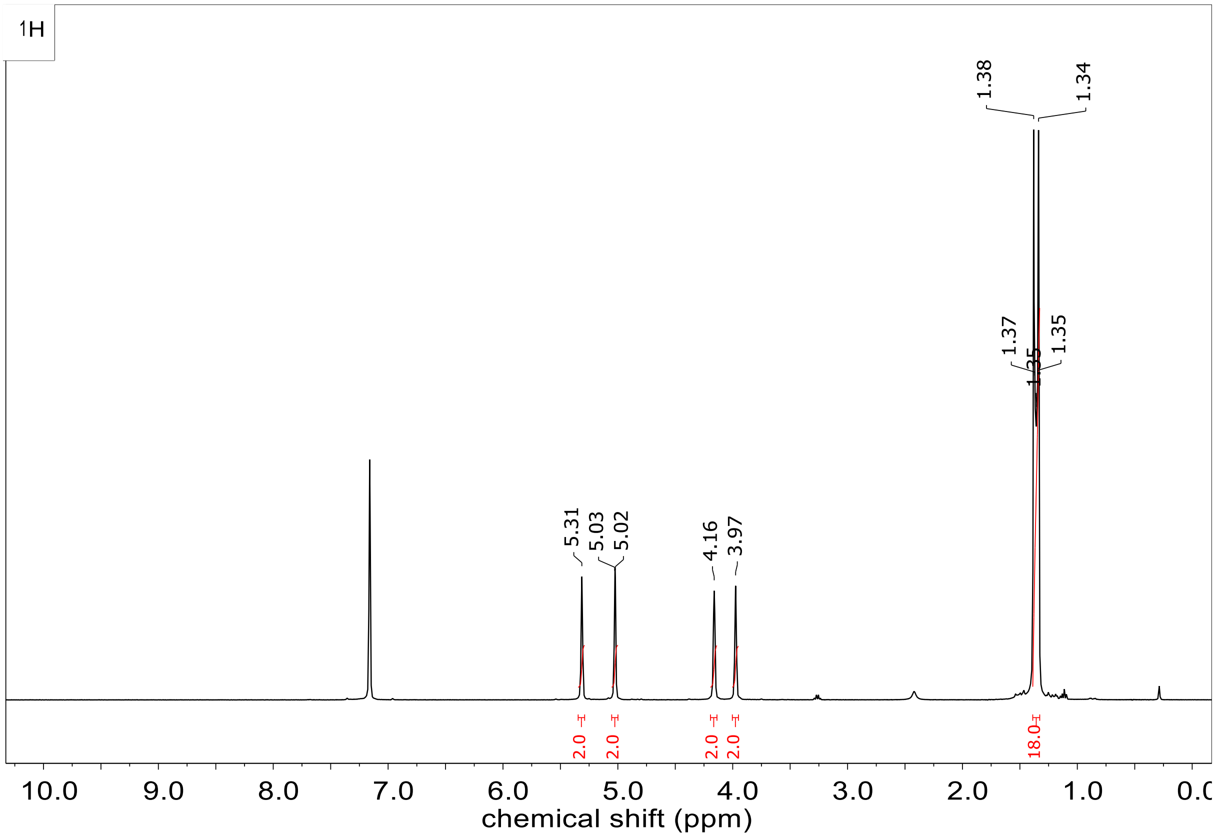

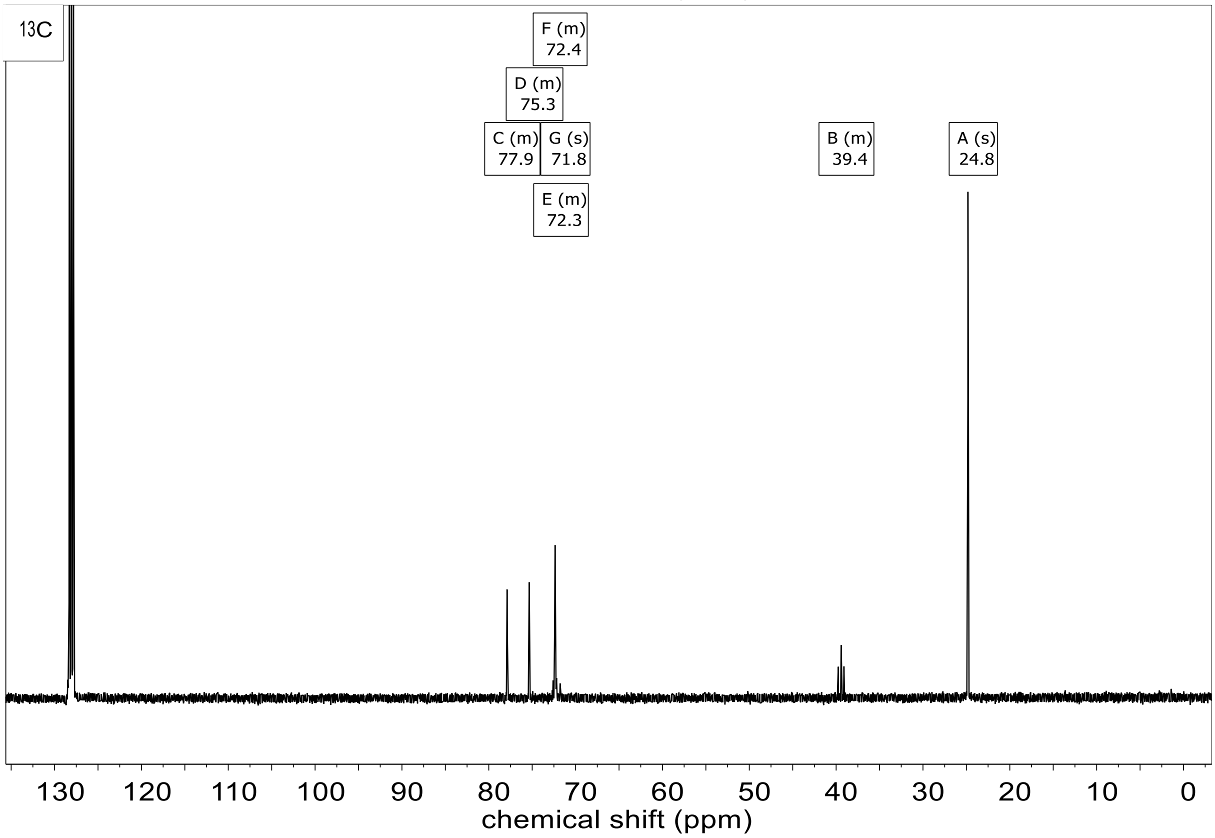

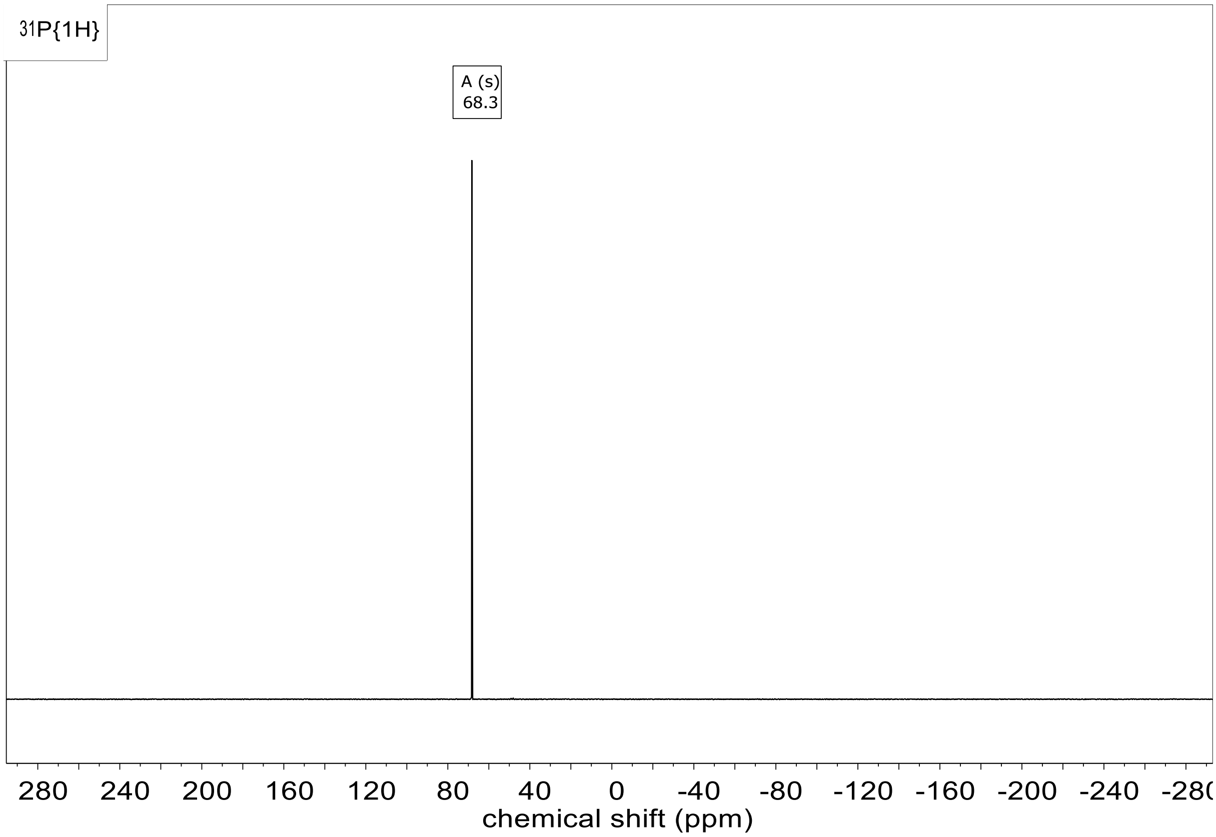

NMR-spectra of **4b**:

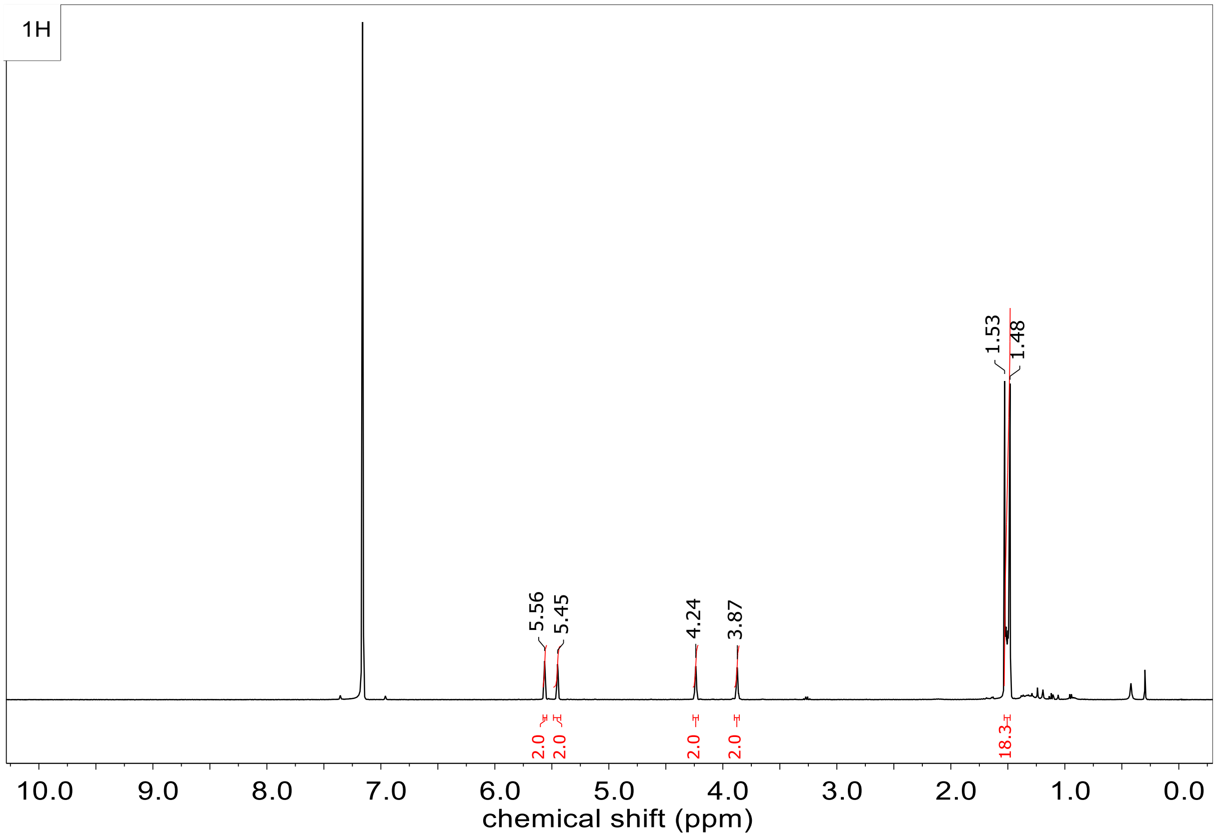

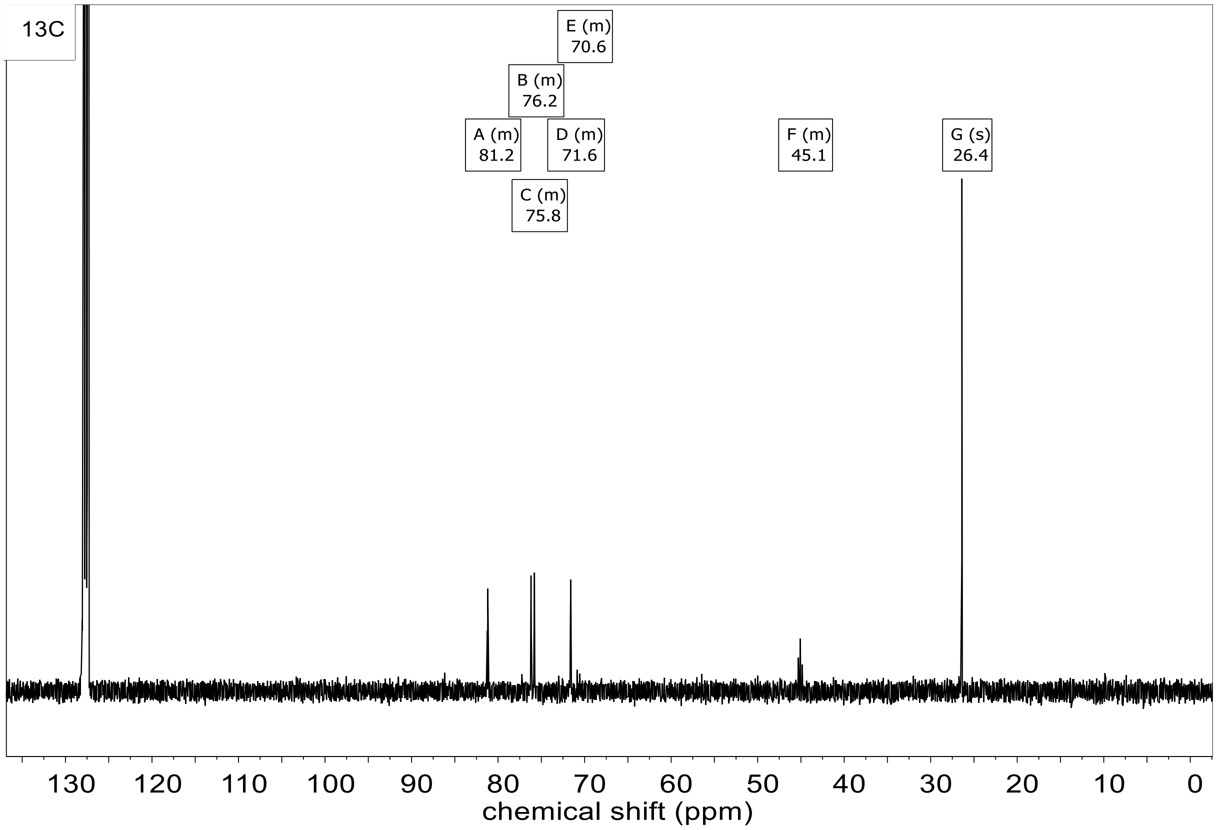
13C
E (m)
70.6
B (m)
76.2
A (m)
81.2
D (m)
71.6
F (m)
45.1
G (s)
26.4
C (m)
75.8
130 120 110 100 90 80 70 60 50 40 30 20 10 0
chemical shift (ppm)

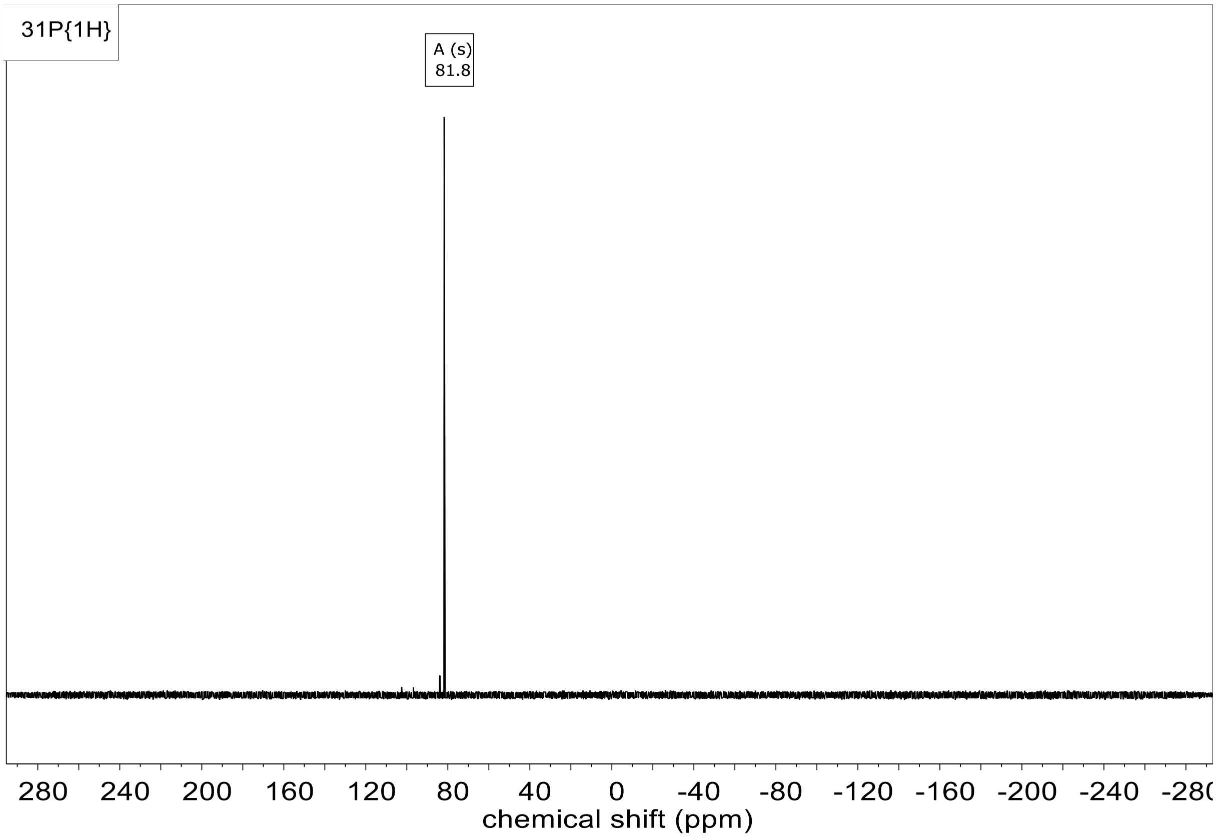
31P{1H}
A (s)
81.8
280 240 200 160 120 80 40 0 -40 -80 -120 -160 -200 -240 -280
chemical shift (ppm)

NMR-spectra of **4c**:

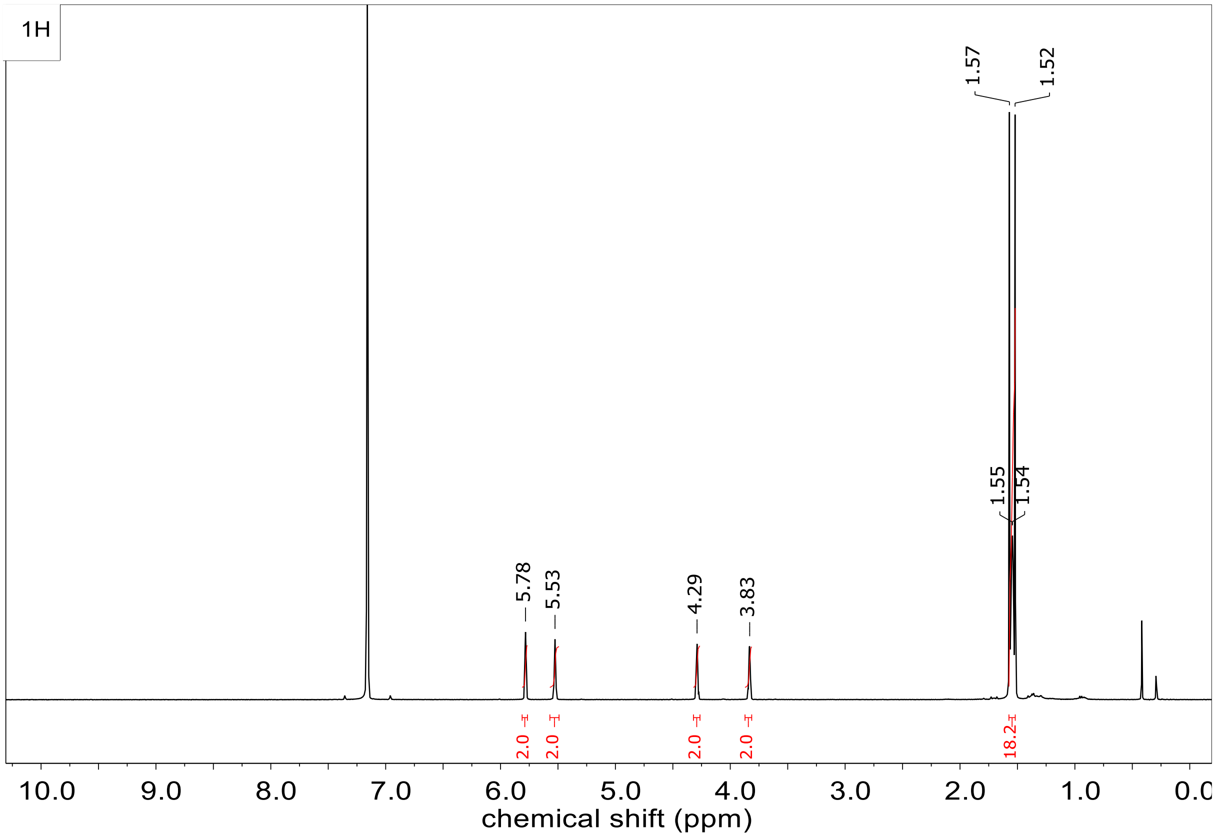

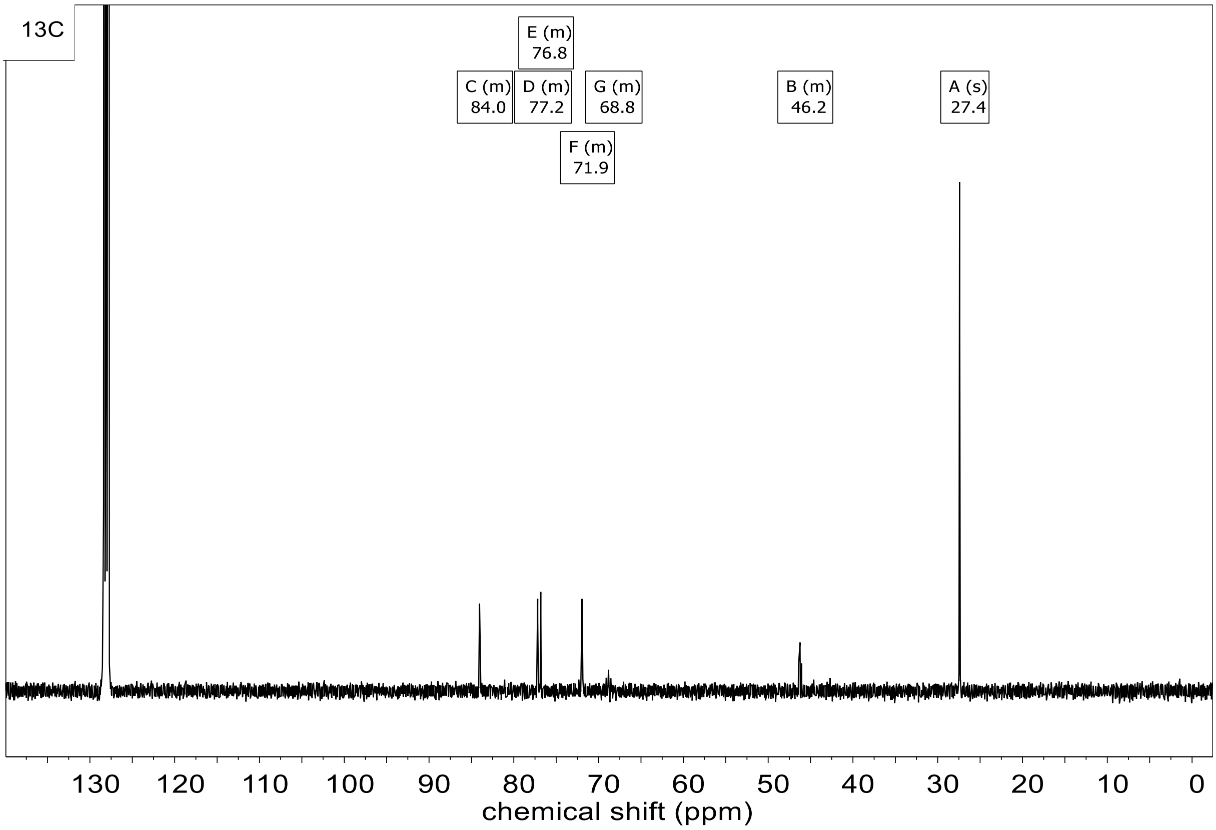

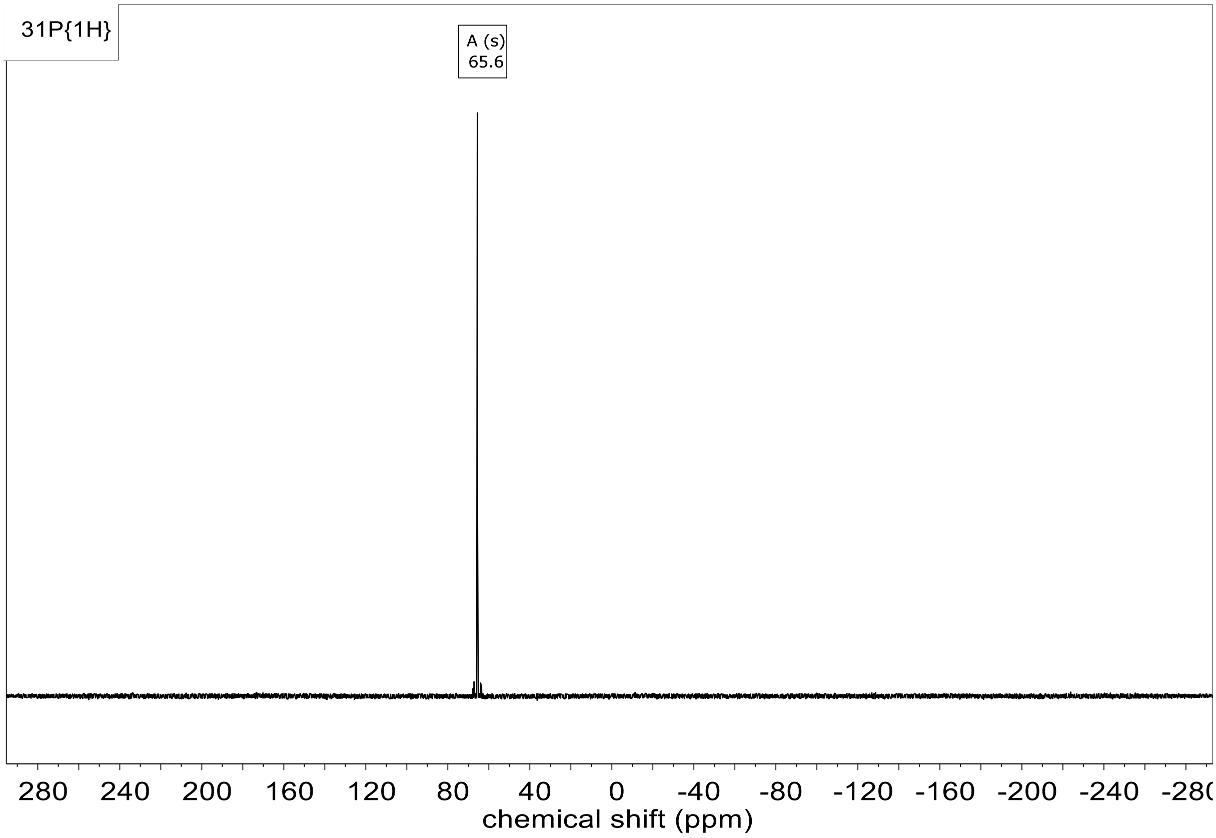
31P{1H}
A (s)
65.6
280 240 200 160 120 80 40 0 -40 -80 -120 -160 -200 -240
chemical shift (ppm)

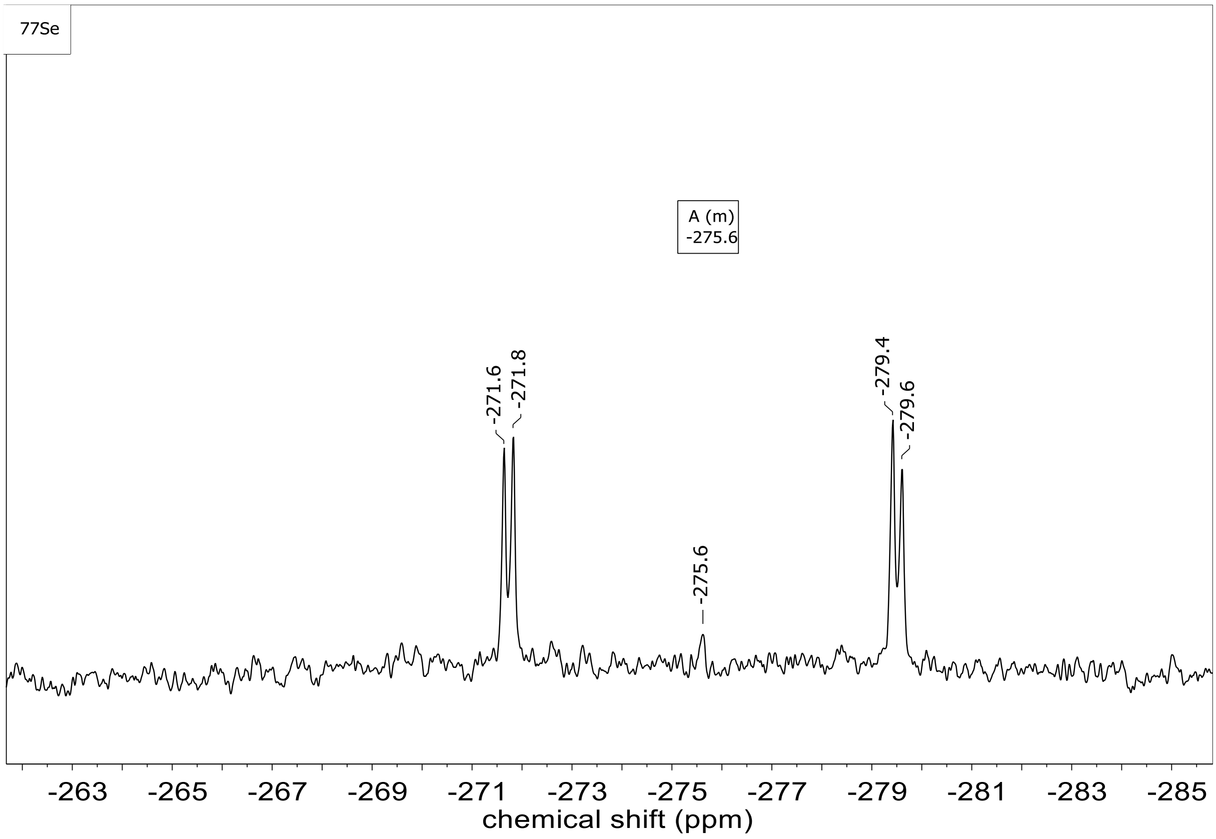
77Se
A (m)
-275.6
-271.6
-271.8
-275.6
-279.4
-279.6
-263 -265 -267 -269 -271 -273 -275 -277 -279 -281 -283 -285
chemical shift (ppm)

NMR-spectrum of **5a**:

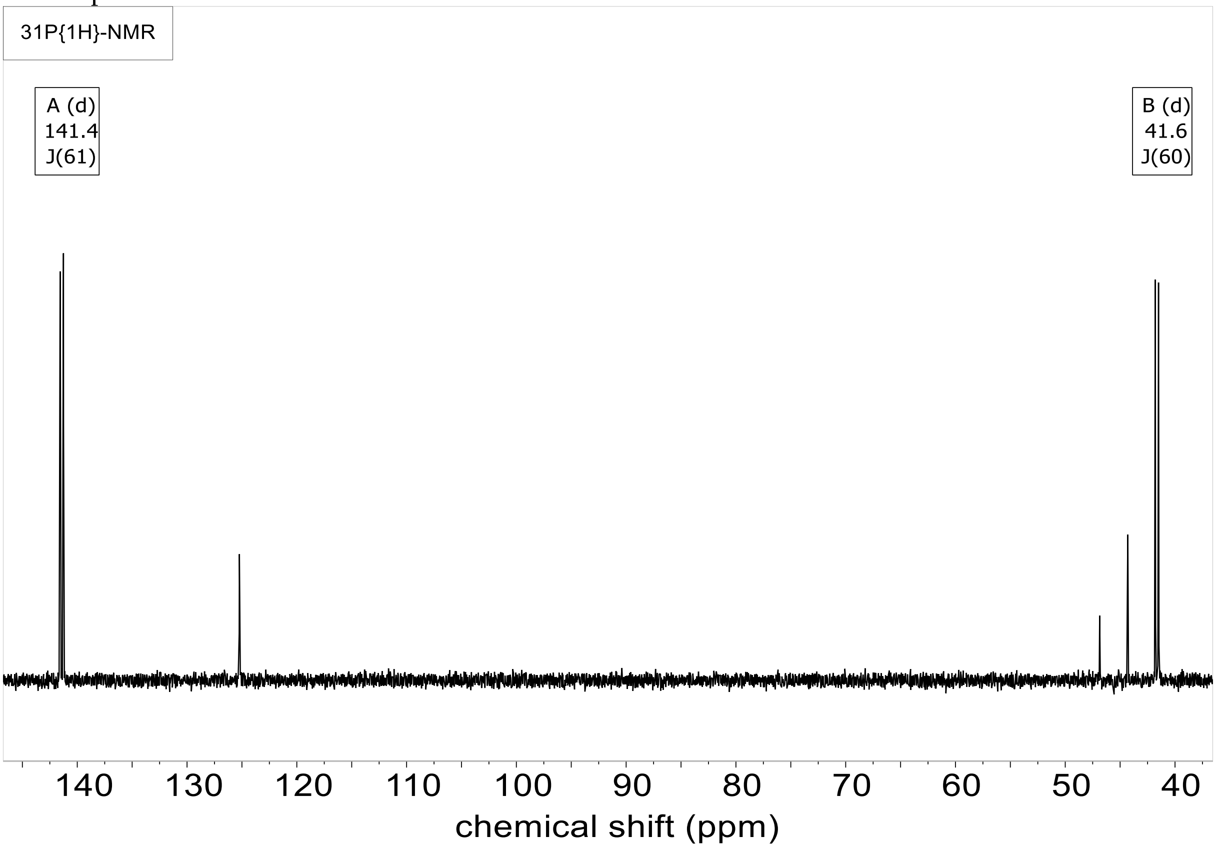

NMR-spectrum of **5b**:

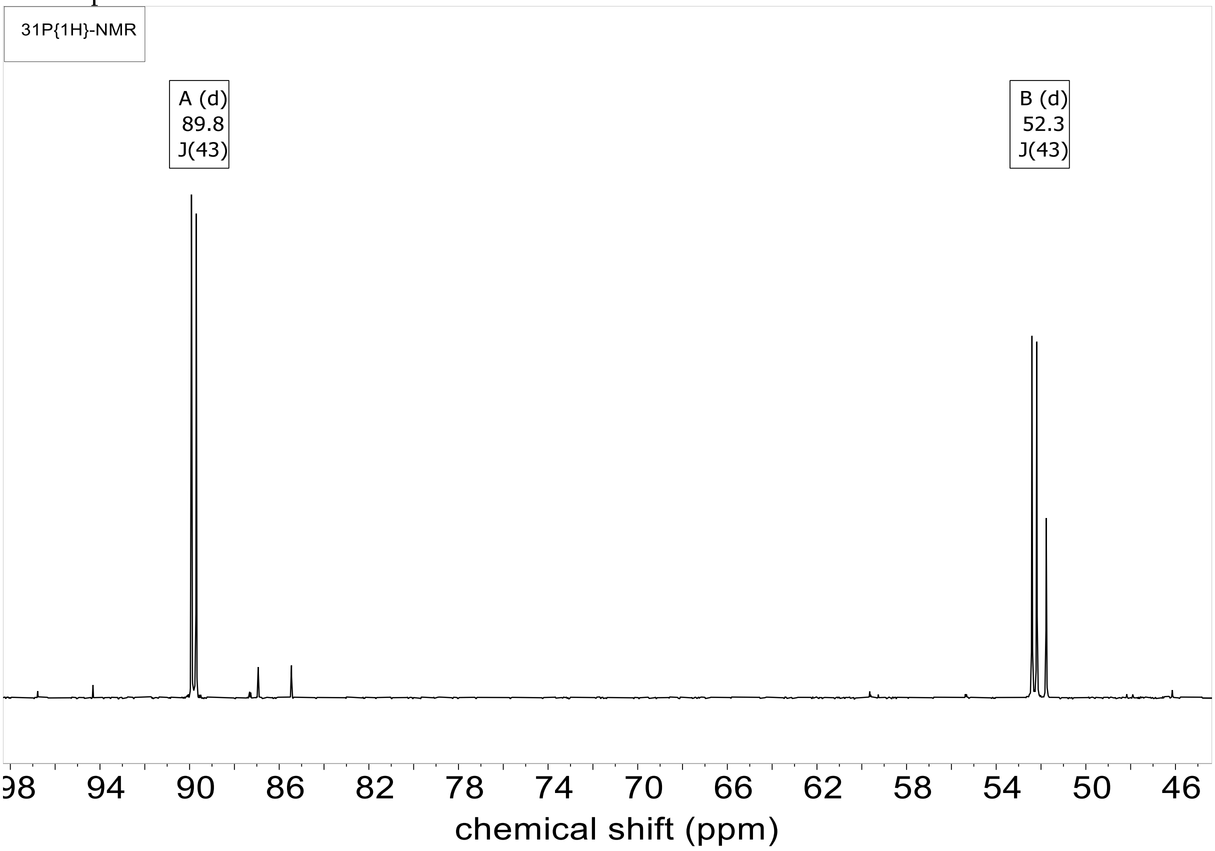

NMR-spectra of **5c**:

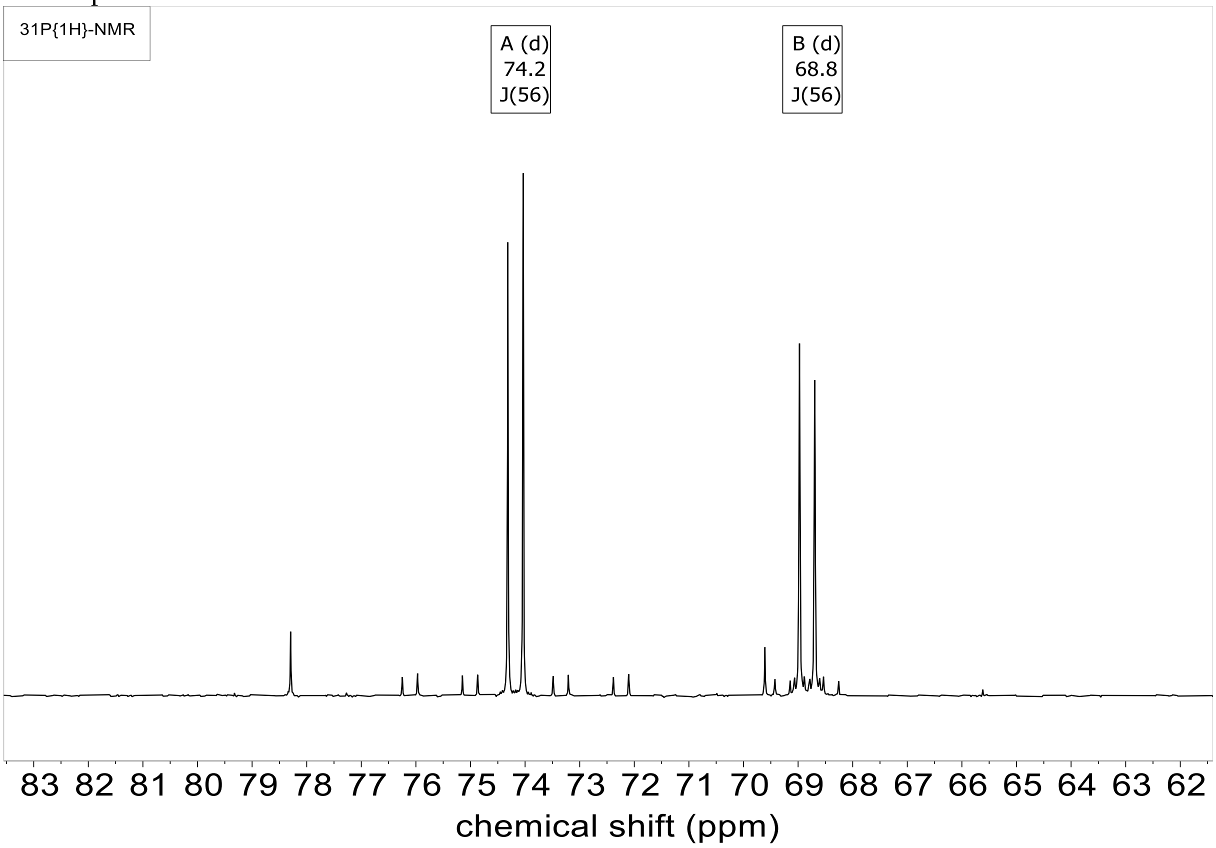

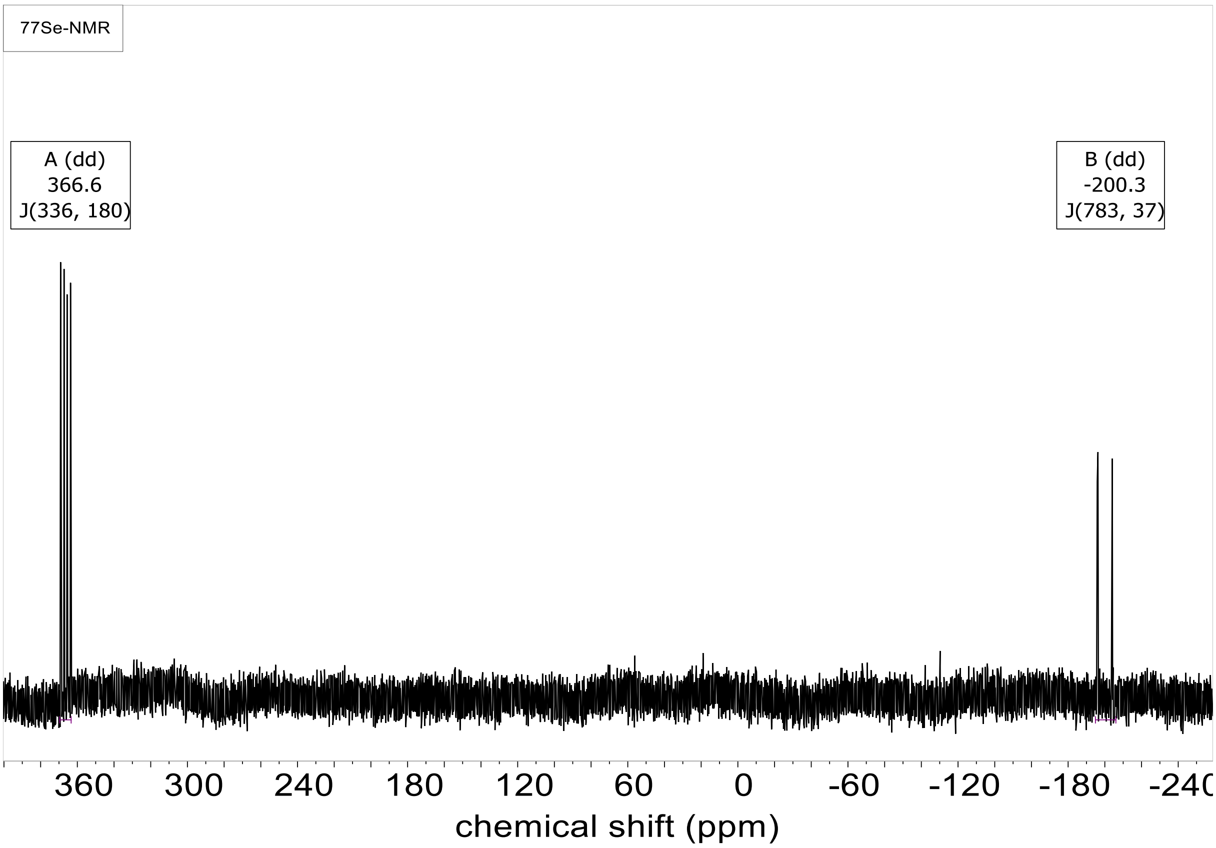

XYZ coordinates and total energies (in atomic unit) of the investigated systems
1
G(ωB97XD/6-311G**)= -2647.63
E(ωB97XD/6-311G**)= -2647.980484

C	-0.037187	-0.060318	0.058603
C	-0.010213	-0.046124	1.478365
C	1.360139	-0.014741	1.909870
C	2.161514	0.010317	0.721646
C	1.304595	-0.032655	-0.408760
Fe	1.005813	-1.657509	0.828065
C	-0.052357	-3.251172	1.513006
C	0.391408	-3.522408	0.192598
C	1.809865	-3.436431	0.180076
C	2.243074	-3.100616	1.490117
C	1.087800	-2.968453	2.334477
P	0.915508	-2.220138	4.006578
C	2.042750	-3.312098	5.065708
C	3.480802	-3.475971	4.566264
P	2.093517	-0.336233	3.566475
C	1.323711	1.027878	4.630081
C	1.781406	0.818762	6.078010
C	1.352010	-4.684801	5.087877
C	2.067931	-2.735232	6.485674
C	1.927321	2.334284	4.090741
C	-0.204059	1.123297	4.596642
H	-0.873049	-0.119358	2.121369
H	-0.923449	-0.110129	-0.555889
H	1.618230	-0.051052	-1.441467
H	3.241277	-0.000813	0.704729
H	-1.079304	-3.199877	1.843227
H	-0.239163	-3.745051	-0.654751
H	2.447570	-3.575650	-0.679861
H	3.263425	-2.907548	1.781496
H	1.922550	-5.372315	5.722022
H	0.336615	-4.618027	5.488982
H	1.296161	-5.119322	4.085761
H	2.540052	-3.453266	7.164903
H	2.650498	-1.812232	6.531145
H	1.060544	-2.530779	6.861152
H	4.063469	-4.028276	5.312562
H	3.517095	-4.047842	3.637849
H	3.967795	-2.510822	4.404055
H	1.563693	3.179390	4.685638
H	3.019653	2.326149	4.146562
H	1.639172	2.506816	3.049891
H	1.524115	1.700419	6.674922
H	1.283285	-0.039970	6.533899
H	2.863598	0.671313	6.146796

2a-*cis*

E(ωB97XD/6-311+G**)=-2723.234233

C	0.034941	0.056528	0.006363
C	0.036309	0.050527	1.425489
C	1.387233	0.043780	1.866125
C	2.224787	0.054186	0.721855
C	1.396632	0.043844	-0.447976
Fe	1.024222	-1.596567	0.657340
C	0.451604	-3.127257	-0.548004
C	1.879431	-2.990435	-0.510937
C	2.274105	-3.193223	0.854938
C	1.114373	-3.422789	1.636836
C	-0.012075	-3.381702	0.770169
P	3.072234	-2.236215	-1.671079
C	3.217543	-3.416296	-3.131077
C	3.887197	-4.663413	-2.524083
P	2.128179	-0.230123	-2.104047
O	3.243978	0.717557	-2.422399
C	0.764081	-0.013105	-3.368007
C	-0.390242	-1.012711	-3.282015
C	0.243658	1.424881	-3.180925
C	1.426126	-0.099001	-4.751920
C	4.173156	-2.773991	-4.145440
C	1.916795	-3.846544	-3.808311
H	3.304290	0.027535	0.714139
H	1.720528	0.017250	2.892519
H	-0.840090	0.038803	2.055800
H	-0.850780	0.046087	-0.606682
H	3.287318	-3.115390	1.220993
H	1.088708	-3.581769	2.704329
H	-1.044183	-3.505055	1.062959
H	-0.172904	-3.005272	-1.417944
H	4.092703	-5.386794	-3.320593
H	4.834654	-4.415818	-2.038204
H	3.239708	-5.148607	-1.787856
H	4.359743	-3.473094	-4.967969
H	3.766387	-1.854002	-4.571340
H	5.132557	-2.526253	-3.684355
H	2.128188	-4.637182	-4.537587
H	1.200338	-4.246971	-3.086328
H	1.447130	-3.025762	-4.349409
H	-1.103560	-0.799025	-4.085092
H	-0.051093	-2.040502	-3.411659
H	-0.932064	-0.952884	-2.338494
H	0.693487	0.183654	-5.514855
H	2.278946	0.578857	-4.819114
H	1.769670	-1.108479	-4.984638
H	-0.462926	1.651552	-3.986424
H	-0.275341	1.565895	-2.231283
H	1.063293	2.144627	-3.231686

2a

E(ωB97XD/6-311+G**)=-2723.249804

C	0.022333	0.023477	-0.006553
C	-0.007791	-0.036880	1.422770
C	1.330487	-0.037448	1.894532
C	2.198814	0.034024	0.770308
C	1.400852	0.075647	-0.404907
Fe	0.977618	-1.626085	0.606628
C	1.923236	-3.467448	0.695104
C	0.767704	-3.459171	1.524674
C	-0.356972	-3.137728	0.719207
C	0.095935	-2.939696	-0.630364
C	1.512919	-3.160296	-0.628291
P	-0.753672	-2.191525	-2.076151
C	-2.255693	-3.308063	-2.349306
C	-3.078921	-3.632004	-1.098193
P	-1.465281	-0.347835	-0.993229
O	-2.678003	-0.523384	-0.124681
C	-1.650728	1.082787	-2.168833
C	-2.873223	0.850642	-3.062326
C	-0.404547	1.295076	-3.034921
C	-1.890055	2.314218	-1.279024
C	-3.162807	-2.653607	-3.396315
C	-1.664644	-4.606050	-2.924223
H	-1.365457	-2.984255	1.070528
H	0.750614	-3.637912	2.589449
H	2.935466	-3.661583	1.016221
H	2.156641	-3.049681	-1.488560
H	-0.907834	-0.128469	2.011670
H	1.636328	-0.101159	2.927930
H	3.278123	0.033623	0.798423
H	1.771022	0.082388	-1.418428
H	-2.040643	3.191777	-1.916282
H	-2.776259	2.183381	-0.654381
H	-1.034526	2.513662	-0.627583
H	-3.090246	1.767790	-3.619527
H	-2.692266	0.058841	-3.792342
H	-3.756587	0.594794	-2.472216
H	-0.606611	2.093793	-3.756332
H	0.454450	1.606709	-2.437664
H	-0.136211	0.395520	-3.595396
H	-2.476673	-5.312715	-3.127833
H	-1.125681	-4.428507	-3.859371
H	-0.978963	-5.081757	-2.216750
H	-3.907501	-3.381656	-3.735226
H	-3.703883	-1.804653	-2.974386
H	-2.602356	-2.317727	-4.274633
H	-3.964693	-4.205104	-1.396698
H	-2.511632	-4.252465	-0.402041
H	-3.404941	-2.725855	-0.583578

TS between **2a-*cis*** and **2a**

E(ωB97XD/6-311+G**)= -2723.206465

C	-1.559665	-2.163253	-0.461231
C	-0.596622	-1.574873	0.428321
C	-1.292713	-1.252758	1.642684
C	-2.655551	-1.611374	1.485569
C	-2.818365	-2.179698	0.189999
Fe	-2.060511	-0.261036	0.039058
C	-3.132587	0.901006	-1.286873
C	-3.189465	1.468516	0.015376
C	-1.861274	1.724891	0.444572
C	-0.965229	1.308169	-0.595301
C	-1.770435	0.810671	-1.670357
P	0.852749	1.160803	-0.667554
C	1.603698	2.266187	0.635358
C	3.125842	2.144455	0.468068
P	0.993293	-0.905834	-0.074271
C	2.499568	-2.006794	-0.144052
C	2.032858	-3.392990	-0.607553
C	3.475380	-1.410518	-1.165382
C	3.167715	-2.119847	1.231896
O	1.422860	1.494560	-2.008014
C	1.231701	1.886075	2.070250
C	1.165931	3.704147	0.313988
H	-1.354721	-2.476696	-1.473575
H	-3.745923	-2.537283	-0.231549
H	-3.438399	-1.464698	2.214768
H	-0.848838	-0.766766	2.498518
H	-1.381018	0.404287	-2.591554
H	-3.977588	0.577151	-1.875551
H	-4.083992	1.655109	0.590374
H	-1.589705	2.137643	1.401530
H	1.669289	4.397293	0.996068
H	1.432618	3.975098	-0.709898
H	0.087917	3.839764	0.433952
H	3.619960	2.860724	1.132331
H	3.476979	1.145176	0.744691
H	3.431392	2.350587	-0.559394
H	1.799794	2.515171	2.763702
H	0.173556	2.036028	2.286894
H	1.473161	0.840080	2.274585
H	4.029800	-2.795432	1.176153
H	3.519547	-1.146422	1.582084
H	2.472466	-2.516539	1.976385
H	4.364926	-2.047516	-1.221803
H	3.024559	-1.342533	-2.156891
H	3.802716	-0.407248	-0.882599
H	2.889719	-4.075725	-0.627132
H	1.285807	-3.813661	0.071684
H	1.601051	-3.349737	-1.609782

3a

E(ωB97XD/6-311+G**)= -2723.245480

C	-0.111006	0.004887	0.098442
C	-0.272301	-0.165554	1.512070
C	1.001403	-0.075818	2.128009
C	1.965333	0.153811	1.107468
C	1.288346	0.200705	-0.140270
Fe	0.975586	-1.601207	0.740757
C	0.400771	-3.183930	-0.422959
C	0.143014	-3.464966	0.959755
C	1.364394	-3.395423	1.675440
C	2.395040	-3.077462	0.748445
C	1.808896	-2.942005	-0.537964
P	-1.004056	-3.034513	-1.589440
C	-0.406156	-3.803276	-3.187286
C	0.855773	-3.169131	-3.778703
P	-1.523033	-0.258540	-1.030765
C	-1.465749	1.188271	-2.215517
C	-0.139652	1.353202	-2.961804
O	-0.885129	-1.423041	-2.063028
C	-1.567263	-3.629338	-4.180151
C	-0.178607	-5.293735	-2.905335
C	-1.774162	2.445068	-1.391774
C	-2.593268	0.938771	-3.229452
H	-0.833834	-3.661470	1.377174
H	1.487045	-3.534547	2.739025
H	3.438026	-2.929920	0.987049
H	2.334884	-2.650312	-1.431830
H	-1.211277	-0.364869	2.007612
H	1.207592	-0.191387	3.181563
H	3.032301	0.238209	1.252316
H	1.758635	0.303638	-1.104787
H	0.993287	-3.518942	-4.808116
H	0.778693	-2.079608	-3.797324
H	1.749019	-3.452866	-3.220841
H	-1.331860	-4.141876	-5.119143
H	-2.499483	-4.048173	-3.790128
H	-1.735390	-2.572198	-4.403147
H	0.078051	-5.811139	-3.835866
H	0.640517	-5.446377	-2.196652
H	-1.076611	-5.768785	-2.497573
H	-0.257477	2.095938	-3.758924
H	0.651129	1.709291	-2.299572
H	0.176747	0.411714	-3.416929
H	-2.672008	1.790325	-3.914046
H	-2.396291	0.043303	-3.824932
H	-3.560485	0.813151	-2.734039
H	-1.844180	3.312507	-2.056818
H	-2.726781	2.356202	-0.859925
H	-0.988645	2.646402	-0.657444

3a-*trans*

E(ωB97XD/6-311+G**)= -2723.244638

C	-0.033782	-0.026507	-0.057454
C	-0.140617	0.078358	1.357175
C	1.172532	0.118352	1.893715
C	2.107697	0.038079	0.812714
C	1.343530	-0.047036	-0.397082
Fe	0.936071	-1.612218	0.842183
C	-0.102298	-3.398183	0.770874
C	0.524145	-3.234412	2.037604
C	1.920388	-3.092636	1.828473
C	2.177302	-3.159768	0.416527
C	0.908239	-3.359276	-0.221798
P	3.663823	-2.870926	-0.614564
C	4.979701	-3.955530	0.169596
C	4.541523	-5.404324	-0.084797
P	3.884145	-0.290359	1.076130
C	4.712919	1.213196	0.329593
C	6.222656	0.935882	0.350650
O	4.184368	-1.339946	-0.197393
C	5.225896	-3.734958	1.663635
C	6.273347	-3.658036	-0.604480
C	4.381996	2.390926	1.255616
C	4.259899	1.515812	-1.099386
H	0.758625	-3.422866	-1.289856
H	-1.162033	-3.506859	0.595168
H	0.021730	-3.188486	2.992542
H	2.649530	-2.891041	2.597218
H	1.752224	-0.169679	-1.388708
H	-0.858192	-0.110991	-0.749888
H	-1.058066	0.091007	1.926307
H	1.432091	0.148641	2.942065
H	7.071104	-4.328238	-0.266255
H	6.142672	-3.803993	-1.680978
H	6.602927	-2.628766	-0.438732
H	5.328429	-6.091231	0.244858
H	3.629021	-5.649816	0.466581
H	4.360007	-5.592062	-1.147959
H	6.116870	-4.293777	1.972890
H	5.395896	-2.680998	1.898238
H	4.391824	-4.096687	2.266802
H	4.904576	3.288700	0.908912
H	3.309835	2.609048	1.261348
H	4.699713	2.199228	2.285680
H	4.853024	2.342415	-1.506723
H	4.396606	0.645834	-1.745659
H	3.209625	1.814182	-1.130285
H	6.768369	1.815353	-0.007902
H	6.576253	0.710290	1.361686
H	6.475313	0.093074	-0.298018

TS between **3a-*trans*** and **3a**

E(ωB97XD/6-311+G**)= -2723.168058

C	0.203650	0.003584	0.068329
C	0.246955	0.083171	1.500931
C	1.638240	0.107941	1.875452
C	2.417143	0.048895	0.696236
C	1.531561	-0.003132	-0.419803
Fe	1.173569	1.696527	0.690278
C	-0.025089	3.265453	0.187799
C	0.336415	3.344371	1.572417
C	1.763915	3.423609	1.628145
C	2.269985	3.400895	0.302307
C	1.164146	3.304777	-0.586899
P	-0.687324	3.164274	3.078336
O	-1.731677	1.937457	2.561977
P	-1.122098	0.388677	2.598139
C	-2.018695	-0.755876	3.746192
C	-1.313570	-2.110226	3.613152
C	-3.495415	-0.880515	3.344643
C	-1.921336	-0.237740	5.187629
C	-1.894634	4.591481	2.953318
C	-2.884445	4.401033	4.113527
C	-2.663601	4.669811	1.632792
C	-1.076643	5.871222	3.169970
H	1.996831	0.194401	2.889628
H	3.495522	0.075833	0.645174
H	1.826842	-0.020027	-1.458596
H	-0.701357	-0.001603	-0.519809
H	2.349344	3.453391	2.535569
H	3.311782	3.418912	0.019222
H	1.222031	3.232744	-1.662934
H	-1.026177	3.139297	-0.193390
H	-4.021016	-1.527208	4.057753
H	-3.983998	0.096895	3.351750
H	-3.594224	-1.304253	2.343598
H	-2.489561	-0.893681	5.858190
H	-0.883019	-0.201283	5.521380
H	-2.339539	0.768989	5.269847
H	-1.814012	-2.831726	4.267055
H	-1.361532	-2.490301	2.589465
H	-0.264749	-2.049346	3.913748
H	-3.447618	5.432015	1.709740
H	-2.010032	4.953213	0.805690
H	-3.139230	3.715262	1.395110
H	-3.567069	5.256316	4.164145
H	-3.483948	3.497657	3.972679
H	-2.367680	4.324028	5.075158
H	-1.748314	6.735581	3.209800
H	-0.516901	5.841468	4.110478
H	-0.366905	6.035658	2.353714

TS between **2a-*cis*** and **3a-*trans***

E(ωB97XD/6-311+G**)= -2723.1687837

C	-2.335840	1.216319	0.190124
C	-1.341983	1.009731	-0.826994
C	-1.905251	0.077276	-1.760768
C	-3.200305	-0.287438	-1.316985
C	-3.468039	0.417612	-0.112396
Fe	-1.772209	-0.742856	0.101302
C	-0.664208	-1.167182	1.758058
C	-1.819514	-1.993703	1.749329
C	-1.807001	-2.758169	0.550407
C	-0.642253	-2.411340	-0.184147
C	0.074115	-1.415744	0.555365
P	1.362897	-0.341029	-0.156924
C	2.946392	-1.310263	-0.071323
C	2.782111	-2.585231	-0.905397
P	0.368558	1.651612	-1.037200
O	0.977127	-0.184635	-1.704621
C	0.608296	2.796041	0.495978
C	2.063096	3.266249	0.371274
C	0.408189	2.240347	1.899545
C	-0.321802	3.987403	0.208019
C	4.048182	-0.427855	-0.669544
C	3.247980	-1.637770	1.393062
H	-0.376188	-2.767648	-1.167692
H	-2.566085	-3.459330	0.237188
H	-2.585688	-2.016969	2.509637
H	-0.405932	-0.447238	2.518835
H	-1.378389	-0.325926	-2.611228
H	-3.855150	-1.000941	-1.794404
H	-4.366161	0.347221	0.483004
H	-2.253353	1.852411	1.054369
H	-0.052526	4.826777	0.858802
H	-0.219901	4.335336	-0.826156
H	-1.373535	3.752871	0.379322
H	2.282999	4.015863	1.139311
H	2.757827	2.431591	0.512671
H	2.263748	3.712853	-0.606931
H	0.626079	3.016241	2.642895
H	-0.601392	1.876305	2.083257
H	1.101509	1.412182	2.076328
H	4.196497	-2.180399	1.464044
H	3.340638	-0.728730	1.996581
H	2.467711	-2.264727	1.833855
H	5.002655	-0.964425	-0.652187
H	3.821452	-0.165822	-1.706006
H	4.173720	0.499527	-0.102586
H	3.749480	-3.090918	-0.997362
H	2.079990	-3.281210	-0.439668
H	2.425582	-2.349997	-1.912549

2b-*cis*

E(ωB97XD/6-311+G**)= -3046.211470

C	0.040417	0.056909	-0.004558
C	0.029453	0.047744	1.414638
C	1.375596	0.029732	1.867283
C	2.223473	0.038203	0.731072
C	1.406452	0.036880	-0.444867
Fe	1.011834	-1.603786	0.654778
C	0.429342	-3.139692	-0.544512
C	1.857286	-3.004689	-0.510741
C	2.254988	-3.204720	0.856182
C	1.096239	-3.428278	1.640448
C	-0.031335	-3.387677	0.775546
P	3.070321	-2.254397	-1.654078
C	3.212269	-3.412867	-3.131132
C	3.870273	-4.668831	-2.528349
P	2.152318	-0.228331	-2.100501
S	3.651220	1.000336	-2.467331
C	0.771124	-0.008283	-3.372979
C	-0.360352	-1.031293	-3.242351
C	0.222049	1.417683	-3.192409
C	1.389281	-0.111689	-4.773472
C	4.179200	-2.764989	-4.131361
C	1.914720	-3.832511	-3.820191
H	3.303119	0.011362	0.730160
H	1.700531	-0.001186	2.896182
H	-0.853263	0.042685	2.036204
H	-0.840916	0.058615	-0.623035
H	3.268781	-3.124361	1.219988
H	1.070996	-3.580193	2.708977
H	-1.063030	-3.506811	1.071613
H	-0.198421	-3.025989	-1.412700
H	4.087103	-5.381650	-3.331169
H	4.810857	-4.432109	-2.024021
H	3.209439	-5.163545	-1.810539
H	4.357841	-3.452433	-4.965437
H	3.789028	-1.829609	-4.538657
H	5.140393	-2.539600	-3.662802
H	2.127086	-4.626518	-4.545148
H	1.188474	-4.225275	-3.103919
H	1.458074	-3.009804	-4.369225
H	-1.089276	-0.848504	-4.038601
H	-0.001630	-2.053578	-3.355640
H	-0.889305	-0.961394	-2.293498
H	0.615757	0.113009	-5.514720
H	2.207877	0.598506	-4.898198
H	1.768786	-1.112485	-4.986323
H	-0.536856	1.597938	-3.960995
H	-0.243559	1.568398	-2.217262
H	1.014923	2.158414	-3.310227

2b

E(ωB97XD/6-311+G**)=-3046.227320

C	-0.136886	-0.102144	0.074146
C	-0.103807	-0.079059	1.493244
C	1.270065	-0.015342	1.909596
C	2.066072	0.015918	0.716618
C	1.201387	-0.047382	-0.406145
Fe	0.945757	-1.682218	0.842687
C	-0.083341	-3.296666	1.555702
C	0.330420	-3.566886	0.226854
C	1.747493	-3.462706	0.171070
C	2.218883	-3.121715	1.467216
C	1.079545	-3.008282	2.336680
P	0.988562	-2.253564	3.998830
C	2.103262	-3.313134	5.081321
C	3.538949	-3.373154	4.544687
P	2.035991	-0.296210	3.549671
C	1.325895	1.076437	4.637913
C	1.564421	0.716717	6.107109
C	1.494909	-4.723360	5.082685
C	2.113459	-2.751298	6.505797
C	2.174817	2.304875	4.265535
C	-0.152803	1.404553	4.417817
H	-0.954263	-0.172795	2.151068
H	-1.025772	-0.175570	-0.534484
H	1.507069	-0.065036	-1.441350
H	3.145908	0.025966	0.693694
H	-1.095726	-3.250239	1.927319
H	-0.321475	-3.794848	-0.602850
H	2.362678	-3.595760	-0.706191
H	3.245236	-2.919559	1.729059
H	2.122144	-5.375666	5.698858
H	0.484656	-4.720467	5.495970
H	1.456091	-5.146909	4.075166
H	2.633545	-3.457254	7.160963
H	2.645214	-1.799159	6.558724
H	1.100225	-2.611639	6.888649
H	4.161745	-3.896252	5.277466
H	3.598145	-3.935061	3.611542
H	3.966381	-2.379782	4.389057
H	1.831597	3.168044	4.846286
H	3.235408	2.148319	4.481495
H	2.072933	2.557469	3.205476
H	1.324210	1.582047	6.733924
H	0.924448	-0.110506	6.422210
H	2.609755	0.452874	6.296176
H	-0.449165	2.186006	5.127025
H	-0.328595	1.789946	3.411614
H	-0.789843	0.534525	4.583007
S	-0.867499	-2.182917	4.681870

G(ωB97XD/6-311+G**)=
E(ωB97XD/6-311+G**)=-3046.211149

C	0.005842	-0.003718	-0.009966
C	0.024039	0.012028	1.419964
C	1.375964	0.000607	1.845893
C	2.206790	-0.010150	0.692946
C	1.371918	-0.008114	-0.455307
Fe	0.946346	-1.633475	0.656385
C	1.860709	-3.475629	0.835709
C	0.702992	-3.412176	1.659124
C	-0.413747	-3.114720	0.836471
C	0.047150	-2.979594	-0.517083
C	1.460907	-3.221168	-0.500842
P	-0.787102	-2.273074	-1.987258
C	-2.251059	-3.426704	-2.291355
C	-2.979984	-3.925914	-1.041860
P	-1.496255	-0.368433	-0.984460
S	-3.111640	-0.457637	0.153248
C	-1.597230	1.007240	-2.259670
C	-2.828317	0.795947	-3.145221
C	-0.339540	1.073402	-3.134706
C	-1.743200	2.315490	-1.468116
C	-3.241574	-2.730227	-3.228660
C	-1.613624	-4.625993	-3.014895
H	-1.422958	-2.938355	1.175753
H	0.679207	-3.540813	2.730703
H	2.869059	-3.668400	1.169066
H	2.109687	-3.156492	-1.361828
H	-0.856650	-0.021619	2.042923
H	1.714010	-0.016456	2.870759
H	3.285812	-0.039178	0.684499
H	1.710509	-0.068005	-1.477392
H	-1.786059	3.150794	-2.174180
H	-2.655683	2.318146	-0.869345
H	-0.891862	2.477903	-0.801309
H	-2.963264	1.677747	-3.779199
H	-2.707973	-0.067805	-3.802174
H	-3.732923	0.661807	-2.548276
H	-0.503625	1.817545	-3.920451
H	0.532857	1.393817	-2.563631
H	-0.117195	0.118891	-3.617427
H	-2.389586	-5.367575	-3.233276
H	-1.147003	-4.331827	-3.959105
H	-0.855713	-5.112198	-2.392908
H	-3.992436	-3.453693	-3.562974
H	-3.766321	-1.918284	-2.720154

TS between **2b-*cis*** and **2b**

E(ωB97XD/6-311+G**)= -3046.181271

C	-0.008147	-0.000361	0.033669
C	0.001300	-0.015605	1.457718
C	1.350378	-0.019473	1.891037
C	2.198391	-0.010761	0.730848
C	1.335965	0.019020	-0.417126
Fe	1.042405	-1.647290	0.721262
C	2.454886	-3.069783	0.514983
C	1.833925	-3.188926	1.798645
C	0.448914	-3.416875	1.604901
C	0.198392	-3.460693	0.206504
C	1.428442	-3.254211	-0.470512
P	4.210739	-2.571925	0.370711
C	4.751609	-2.827810	-1.416260
C	3.977023	-1.941952	-2.397490
P	3.937443	-0.463048	0.721698
C	5.249073	0.811626	1.104381
C	6.488980	0.089594	1.641974
C	5.606470	1.618179	-0.150819
C	4.674061	1.747127	2.175556
S	5.379257	-3.536295	1.627053
C	4.577794	-4.313659	-1.761092
C	6.235058	-2.444518	-1.494787
H	1.699086	-0.079262	2.910715
H	-0.869587	-0.048235	2.095219
H	-0.887089	-0.017822	-0.593352
H	1.671712	-0.001096	-1.442851
H	2.349586	-3.081437	2.740983
H	-0.288601	-3.518025	2.386634
H	-0.761212	-3.609477	-0.265254
H	1.546245	-3.228102	-1.540432
H	4.951314	-4.489848	-2.775046
H	5.141485	-4.946353	-1.072628
H	3.530406	-4.623424	-1.730220
H	6.589391	-2.603078	-2.518204
H	6.382344	-1.386552	-1.254974
H	6.843349	-3.043593	-0.815698
H	4.361128	-2.118684	-3.407411
H	2.907194	-2.147739	-2.411964
H	4.110503	-0.884601	-2.158849
H	6.346568	2.388046	0.098668
H	6.031264	0.976127	-0.926051
H	4.726371	2.115718	-0.566654
H	7.254796	0.836738	1.877972
H	6.261823	-0.482899	2.542530
H	6.911559	-0.601781	0.908951
H	5.400241	2.539197	2.389160
H	3.748663	2.224214	1.840436
H	4.467625	1.206470	3.101648

3b-*trans*

E(ωB97XD/6-311+G**)= -3046.227083

C	-0.041320	0.039492	0.097064
C	0.014060	0.035037	1.516498
C	1.388376	0.012004	1.912337
C	2.171983	0.008844	0.713159
C	1.291953	0.025589	-0.399314
Fe	0.979350	1.675941	0.792795
C	-0.075596	3.318115	0.165004
C	-0.019707	3.264923	1.583427
C	1.353999	3.300261	1.980476
C	2.136849	3.369464	0.782797
C	1.256879	3.380428	-0.329795
P	2.145438	3.185146	3.623506
S	0.999517	1.598692	4.530966
P	2.177933	0.075465	3.559074
C	1.544504	-1.486967	4.405702
C	0.023551	-1.645250	4.463874
C	2.123433	-1.484618	5.827822
C	2.152056	-2.650206	3.606002
C	1.479945	4.697607	4.533893
C	-0.043952	4.821473	4.598181
C	2.062526	5.905405	3.783166
C	2.059664	4.648553	5.954846
H	3.251460	0.024022	0.677304
H	1.582649	0.055897	-1.438717
H	-0.938964	0.085387	-0.501639
H	-0.830956	0.101692	2.184070
H	3.216391	3.378345	0.746812
H	1.547696	3.399282	-1.369436
H	-0.972344	3.278310	-0.435476
H	-0.862856	3.153021	2.247297
H	-0.225619	-2.553999	5.023797
H	-0.456232	-0.801786	4.966956
H	-0.406025	-1.745470	3.465582
H	1.887555	-2.433098	6.322279
H	3.211090	-1.369212	5.818382
H	1.702621	-0.675941	6.431108
H	1.895341	-3.598127	4.090916
H	1.767213	-2.680791	2.582763
H	3.243280	-2.582003	3.560972
H	-0.311739	5.700828	5.195213
H	-0.476142	4.953957	3.604791
H	-0.505674	3.948032	5.065760
H	1.804295	5.570625	6.488055
H	1.656232	3.806985	6.524057
H	3.149492	4.556426	5.940918
H	1.786319	6.826867	4.306822
H	3.154911	5.861988	3.735635
H	1.676504	5.970233	2.761970

3b

E(ωB97XD/6-311+G**)= -3046.223195

C	0.347879	-0.077084	-0.104703
C	0.483120	-0.126527	1.307286
C	1.878438	-0.054660	1.634165
C	2.584537	0.043497	0.391842
C	1.646757	0.038610	-0.670661
Fe	1.274765	1.611742	0.608581
C	0.486735	3.302775	-0.270450
C	-0.020906	3.125000	1.042837
C	1.081352	3.116012	1.955773
C	2.271058	3.300764	1.182207
C	1.901994	3.411796	-0.182773
P	0.868972	2.679756	3.713215
S	2.782664	1.752862	4.114169
P	2.809941	-0.187474	3.206888
C	1.772407	-1.305320	4.321220
C	0.431306	-0.754234	4.807380
C	2.658931	-1.582547	5.546280
C	1.568387	-2.613205	3.540958
C	1.160622	4.331694	4.585452
C	-0.046334	5.204121	4.207288
C	2.452719	5.050211	4.188899
C	1.143676	4.062697	6.096227
H	3.657376	0.128747	0.297728
H	1.877280	0.129075	-1.721566
H	-0.584732	-0.082440	-0.649176
H	-0.334637	-0.174335	2.007593
H	3.276279	3.292697	1.574797
H	2.581464	3.520268	-1.015155
H	-0.096596	3.316984	-1.179023
H	-1.055807	2.968260	1.310893
H	2.150074	-2.286103	6.214037
H	3.619462	-2.019258	5.259753
H	2.853693	-0.666005	6.110360
H	1.136942	-3.368971	4.205909
H	0.892176	-2.482752	2.693334
H	2.516347	-3.007892	3.161147
H	-0.055147	-1.503997	5.442315
H	0.566336	0.151478	5.401795
H	-0.251443	-0.518948	3.991256
H	0.011666	6.155872	4.746435
H	-0.062182	5.426544	3.136665
H	-0.993502	4.725474	4.474653
H	2.530048	5.992599	4.743127
H	3.338281	4.453378	4.419161
H	2.466802	5.286698	3.122416
H	1.187615	5.012340	6.640384
H	0.229816	3.542806	6.399632
H	1.998936	3.457529	6.407466

G(ωB97XD/6-311G**)= -3045.857650
E(ωB97XD/6-311G**)=-3046.210166

C	0.004213	-0.010171	-0.003647
C	0.001872	-0.006149	1.418169
C	1.350017	-0.003955	1.863623
C	2.202589	0.000470	0.715375
C	1.354068	-0.006452	-0.437779
Fe	0.999716	-1.649819	0.725957
C	0.035025	-3.344333	0.085812
C	0.032066	-3.272161	1.505799
C	1.379804	-3.225566	1.950052
C	2.232872	-3.275804	0.803264
C	1.384929	-3.346387	-0.348176
P	4.049714	-3.179952	0.633242
C	4.657296	-4.683884	1.592092
C	4.141787	-5.897074	0.802311
P	4.020979	-0.070751	0.549874
C	4.600106	1.493359	1.426519
C	6.134656	1.484398	1.373614
S	4.508901	-1.583027	2.006803
C	4.189826	-4.782572	3.045741
C	6.191437	-4.649438	1.537862
C	4.130014	1.661126	2.872974
C	4.062748	2.652757	0.573070
H	1.728696	-3.366174	-1.371889
H	-0.836543	-3.361282	-0.551024
H	-0.843808	-3.222334	2.135361
H	1.705749	-3.111775	2.972236
H	1.697926	-0.035228	-1.461248
H	-0.867199	-0.043494	-0.640051
H	-0.873249	-0.038324	2.049923
H	1.677496	-0.056735	2.890278
H	4.646708	-5.661691	3.514022
H	4.480494	-3.905385	3.629346
H	3.106737	-4.900440	3.109594
H	6.595565	-5.565932	1.980635
H	6.554779	-4.581469	0.508525
H	6.593372	-3.799292	2.095088
H	4.528574	-6.816546	1.254119
H	3.049514	-5.949741	0.812983
H	4.474647	-5.870327	-0.239677
H	4.570267	2.572380	3.293297
H	3.044889	1.762176	2.930839
H	4.436509	0.821999	3.502630
H	6.521526	2.430608	1.766486
H	6.551886	0.672899	1.975436

TS between **3b-*trans*** and **3b**

E(ωB97XD/6-311+G**)=-3046.171331

C	0.008038	-0.022325	-0.010466
C	-0.046735	-0.049547	1.418773
C	1.281057	-0.037308	1.922021
C	2.169600	-0.008545	0.811900
C	1.392148	-0.008637	-0.375415
Fe	0.981572	-1.681386	0.721306
C	0.242421	-3.323642	-0.248563
C	-0.082847	-3.362605	1.150560
C	1.128160	-3.371307	1.886873
C	2.208472	-3.313135	0.961533
C	1.668063	-3.298924	-0.349605
P	-0.950162	-3.115624	-1.565139
C	-2.199753	-4.423684	-2.027741
C	-1.891872	-5.015552	-3.408963
P	-1.503377	-0.039060	-1.043086
C	-1.422771	1.651069	-1.888190
C	-0.131010	1.957469	-2.650453
S	-0.818707	-1.366034	-2.635137
C	-3.600472	-3.801726	-2.027180
C	-2.095734	-5.514047	-0.954982
C	-1.614449	2.685506	-0.769114
C	-2.618583	1.706053	-2.850263
H	-1.083844	-3.355289	1.554055
H	1.215082	-3.377804	2.963138
H	3.256700	-3.265464	1.217077
H	2.221400	-3.232990	-1.274163
H	-0.952084	-0.100499	2.005909
H	1.566487	-0.077769	2.962636
H	3.248232	-0.029724	0.861799
H	1.771495	-0.042636	-1.385115
H	-2.646542	-5.768751	-3.666986
H	-1.910454	-4.241689	-4.180379
H	-0.907452	-5.487604	-3.422985
H	-4.337954	-4.554940	-2.328812
H	-3.863511	-3.424355	-1.037289
H	-3.662788	-2.969762	-2.733704
H	-2.791222	-6.321193	-1.207738
H	-1.089976	-5.938849	-0.903904
H	-2.360653	-5.133258	0.033908
H	-0.218061	2.939247	-3.130481
H	0.731519	1.992491	-1.982538
H	0.069046	1.222321	-3.434342
H	-2.694145	2.706248	-3.290571
H	-2.510368	0.987757	-3.668014
H	-3.558853	1.493144	-2.333252
H	-1.688529	3.687952	-1.205395
H	-2.530511	2.501898	-0.198967
H	-0.770914	2.682624	-0.073335

TS between **2b-*cis*** and **3b-*trans***

E(ωB97XD/6-311+G**)= -3046.154606

C	1.759117	-2.149070	-1.688986
C	1.800865	-2.814611	-0.432799
C	0.692192	-2.376519	0.338655
C	-0.039157	-1.421398	-0.433014
C	0.624105	-1.294454	-1.696859
Fe	1.843044	-0.767836	-0.157013
C	1.504373	1.045800	0.673056
C	2.167425	0.188841	1.615162
C	3.409925	-0.215241	1.067240
C	3.550881	0.394775	-0.208191
C	2.388713	1.169290	-0.455023
P	-0.135983	1.790683	1.006098
C	-0.570795	2.781786	-0.571696
C	-0.505806	2.140576	-1.951657
P	-1.358429	-0.317012	0.170902
S	-1.147952	-0.225876	2.235754
C	-2.947582	-1.241294	-0.235968
C	-4.128003	-0.388718	0.237374
C	-3.005456	-1.426069	-1.755915
C	-2.942451	-2.593705	0.477874
C	0.369659	3.999239	-0.477518
C	-2.011007	3.250485	-0.323272
H	0.470590	-2.641472	1.361456
H	2.560455	-3.510416	-0.109111
H	2.478177	-2.253607	-2.487783
H	0.332961	-0.632267	-2.497722
H	1.741603	-0.148584	2.547402
H	4.112210	-0.893070	1.528690
H	4.381542	0.274508	-0.887396
H	2.212294	1.737787	-1.351592
H	0.039740	4.763695	-1.188926
H	0.347171	4.450538	0.519830
H	1.406484	3.747879	-0.709563
H	-2.312969	3.957173	-1.103567
H	-2.706828	2.406273	-0.348039
H	-2.116595	3.747662	0.645285
H	-0.820482	2.870031	-2.706510
H	0.488598	1.789750	-2.222956
H	-1.192177	1.291417	-2.008922
H	-3.937952	-1.932492	-2.026585
H	-2.987184	-0.464191	-2.278313
H	-2.175240	-2.035184	-2.122419
H	-5.066781	-0.909462	0.021124
H	-4.079084	-0.204369	1.313241
H	-4.156494	0.577237	-0.275661
H	-3.905199	-3.093370	0.323295
H	-2.154860	-3.246565	0.093172
H	-2.797673	-2.474493	1.556092

2c

E(ωB97XD/6-311+G**)=-5049.607855

C	-0.133143	-0.102828	0.079759
C	-0.102039	-0.085199	1.498490
C	1.270956	-0.019131	1.916867
C	2.069507	0.024721	0.725473
C	1.205745	-0.038778	-0.398434
Fe	0.959242	-1.678710	0.842176
C	-0.066080	-3.303679	1.533460
C	0.361379	-3.561005	0.206849
C	1.778062	-3.445056	0.164103
C	2.235994	-3.110836	1.466959
C	1.087431	-3.012472	2.327700
P	0.980306	-2.270905	3.996535
C	2.103135	-3.325242	5.083627
C	3.523581	-3.420363	4.512085
P	2.024470	-0.303761	3.562034
C	1.313227	1.078837	4.639472
C	1.524574	0.723805	6.113738
C	1.479894	-4.727259	5.138054
C	2.159387	-2.726244	6.491582
C	2.183401	2.294637	4.272365
C	-0.156201	1.429382	4.393715
H	-0.951824	-0.186777	2.156135
H	-1.021303	-0.178457	-0.529607
H	1.512377	-0.050845	-1.433510
H	3.149325	0.044140	0.702377
H	-1.081343	-3.268815	1.898798
H	-0.282357	-3.788406	-0.629338
H	2.401617	-3.568154	-0.708716
H	3.259038	-2.905633	1.738650
H	2.132436	-5.377190	5.730218
H	0.493398	-4.706596	5.603831
H	1.381964	-5.165528	4.140761
H	2.694644	-3.419708	7.147791
H	2.700379	-1.777695	6.503498
H	1.160243	-2.568748	6.903793
H	4.156386	-3.932065	5.244317
H	3.549699	-4.009665	3.594596
H	3.963240	-2.438817	4.319003
H	1.845725	3.164086	4.846793
H	3.239675	2.124263	4.498309
H	2.093759	2.544628	3.210592
H	1.268984	1.590928	6.732032
H	0.881448	-0.104344	6.420333
H	2.567085	0.465652	6.323758
H	-0.451918	2.216550	5.096773
H	-0.309397	1.815470	3.384128
H	-0.810768	0.571115	4.551026
Se	-1.027241	-2.228145	4.727825

G(ωB97XD/6-311G**)= -5049.239381
E(ωB97XD/6-311G**)= -5049.592608

C	-0.000566	-0.004321	-0.006846
C	0.014233	0.011294	1.423268
C	1.364742	0.003912	1.852741
C	2.199191	-0.004529	0.702229
C	1.367451	-0.004745	-0.448697
Fe	0.944561	-1.629868	0.660378
C	1.862755	-3.466483	0.855019
C	0.694424	-3.406822	1.664083
C	-0.412771	-3.115874	0.827358
C	0.064573	-2.977343	-0.519912
C	1.479296	-3.215277	-0.487468
P	-0.767246	-2.275009	-1.993295
C	-2.211735	-3.451247	-2.311779
C	-2.923579	-3.988634	-1.068359
P	-1.496473	-0.370499	-0.992720
Se	-3.252267	-0.453527	0.218469
C	-1.591110	1.002421	-2.278930
C	-2.789991	0.758873	-3.199446
C	-0.309570	1.088358	-3.117213
C	-1.785337	2.314631	-1.505707
C	-3.220941	-2.761748	-3.233818
C	-1.550666	-4.626141	-3.054856
H	-1.427463	-2.941759	1.151674
H	0.656807	-3.533059	2.735520
H	2.867467	-3.654015	1.202244
H	2.139901	-3.152700	-1.339671
H	-0.869463	-0.024626	2.042148
H	1.698665	-0.012303	2.878996
H	3.278378	-0.029554	0.697200
H	1.710360	-0.061641	-1.469474
H	-1.809017	3.142164	-2.222017
H	-2.721991	2.311923	-0.945973
H	-0.964599	2.495921	-0.806090
H	-2.919378	1.631759	-3.846697
H	-2.632584	-0.108588	-3.843672
H	-3.711691	0.613964	-2.631702
H	-0.467816	1.821376	-3.914527
H	0.537440	1.434395	-2.523464
H	-0.050873	0.135481	-3.585000
H	-2.311989	-5.378911	-3.285779
H	-1.088044	-4.307827	-3.993083
H	-0.785039	-5.107519	-2.438572
H	-3.968269	-3.492770	-3.559740
H	-3.747353	-1.956699	-2.715750

TS between **2c-*cis*** and **2c**

E(ωB97XD/6-311+G**)= -5049.560514

C	-0.224677	-0.912748	-0.536169
C	-0.597159	-0.722341	0.817743
C	0.349042	0.150999	1.419827
C	1.314689	0.498529	0.440900
C	0.973615	-0.171081	-0.782279
Fe	1.300643	-1.526693	0.675107
C	1.482484	-3.317390	1.701344
C	1.470807	-3.510935	0.297355
C	2.560727	-2.765830	-0.271919
C	3.245235	-2.129946	0.818035
C	2.571351	-2.458064	2.021467
P	2.656849	-2.311900	-2.009244
C	3.407545	-3.467476	-3.277922
C	4.302381	-4.441036	-2.502573
P	1.888183	-0.340391	-2.364357
Se	0.660611	-0.211066	-4.097991
C	3.235978	0.989275	-2.363330
C	2.554746	2.354651	-2.182067
C	3.941650	0.967428	-3.723820
C	4.284706	0.732793	-1.276081
C	2.288099	-4.230419	-4.000936
C	4.234225	-2.680681	-4.297605
H	0.740091	-4.068090	-0.269188
H	0.772673	-3.726779	2.404505
H	2.827436	-2.102714	3.008474
H	4.095152	-1.473327	0.716076
H	-0.719718	-1.537151	-1.264748
H	-1.443001	-1.178191	1.309520
H	0.348880	0.483999	2.446739
H	2.163428	1.137832	0.613828
H	3.314472	3.139630	-2.254780
H	1.813970	2.524752	-2.966493
H	2.057258	2.458052	-1.216743
H	4.685987	1.770071	-3.742478
H	4.466040	0.024692	-3.889099
H	3.239799	1.123884	-4.544225
H	5.032852	1.531064	-1.314265
H	3.869772	0.713051	-0.268975
H	4.792659	-0.219897	-1.446177
H	4.711737	-5.177605	-3.202371
H	5.134501	-3.924052	-2.018771
H	3.743355	-4.983989	-1.735605
H	4.632922	-3.373539	-5.047081
H	3.621694	-1.941789	-4.820935
H	5.074889	-2.172544	-3.819719
H	2.723202	-4.910896	-4.742855
H	1.697181	-4.821178	-3.296925
H	1.616176	-3.538618	-4.513248

3c

E(ωB97XD/6-311+G**)= -5049.610799

C	-0.050526	0.042403	0.129304
C	-0.006880	0.029828	1.549025
C	1.363685	0.004070	1.956419
C	2.157511	0.007718	0.763833
C	1.286756	0.031195	-0.355922
Fe	0.964519	1.674685	0.839968
C	-0.085025	3.313198	0.196976
C	-0.041027	3.267941	1.616021
C	1.328874	3.305756	2.024731
C	2.122068	3.368154	0.833355
C	1.251511	3.372665	-0.286789
P	2.110655	3.227256	3.675169
Se	0.857189	1.593918	4.690264
P	2.144303	0.030652	3.609047
C	1.550134	-1.586652	4.385865
C	0.034459	-1.791477	4.429825
C	2.125026	-1.633113	5.808705
C	2.192260	-2.697192	3.538855
C	1.483411	4.798289	4.518231
C	-0.036149	4.969779	4.570170
C	2.102021	5.956135	3.718376
C	2.057891	4.797511	5.941991
H	3.237265	0.022153	0.737457
H	1.586448	0.068293	-1.392589
H	-0.943117	0.092394	-0.476651
H	-0.857027	0.091190	2.210964
H	3.201875	3.377586	0.806870
H	1.551465	3.384804	-1.323972
H	-0.976629	3.269500	-0.410915
H	-0.889401	3.161312	2.274477
H	-0.189459	-2.741113	4.929332
H	-0.470793	-0.997692	4.987110
H	-0.393768	-1.836522	3.426840
H	1.916514	-2.609942	6.258312
H	3.208827	-1.485743	5.808907
H	1.680211	-0.866343	6.448697
H	1.964432	-3.671776	3.983849
H	1.808932	-2.696191	2.514687
H	3.280854	-2.594047	3.498481
H	-0.279338	5.892870	5.108887
H	-0.465741	5.047937	3.569817
H	-0.524649	4.143140	5.093594
H	1.829329	5.750255	6.431744
H	1.629418	3.995710	6.549373
H	3.144521	4.672826	5.936482
H	1.854385	6.906443	4.203485
H	3.192490	5.877245	3.674076
H	1.718228	5.989860	2.694939

G(ωB97XD/6-311G**)= -5049.246997
E(ωB97XD/6-311G**)=-5049.595872

C	0.005754	-0.010386	-0.002002
C	0.005918	-0.006323	1.419754
C	1.354828	-0.003661	1.862485
C	2.205297	0.001403	0.713310
C	1.354871	-0.006361	-0.438704
Fe	0.999969	-1.649633	0.724458
C	0.018705	-3.330757	0.080805
C	0.018613	-3.263937	1.500996
C	1.367413	-3.234013	1.943058
C	2.218129	-3.289730	0.795405
C	1.367901	-3.346032	-0.355405
P	4.037137	-3.240037	0.623966
C	4.589533	-4.809901	1.516816
C	4.026053	-5.967304	0.676772
P	4.024669	-0.042611	0.544194
C	4.564653	1.574079	1.357536
C	6.098361	1.608825	1.297778
Se	4.581586	-1.600486	2.134868
C	4.120722	-4.956697	2.965781
C	6.123474	-4.835688	1.458494
C	4.094367	1.789331	2.797443
C	3.992417	2.683728	0.460882
H	1.710380	-3.366622	-1.379515
H	-0.853834	-3.335408	-0.554998
H	-0.855868	-3.206471	2.131908
H	1.697925	-3.129088	2.964911
H	1.697371	-0.034184	-1.462636
H	-0.866677	-0.044236	-0.637070
H	-0.868206	-0.039078	2.052924
H	1.685959	-0.054934	2.888231
H	4.529300	-5.882167	3.387153
H	4.461922	-4.128716	3.593302
H	3.032873	-5.015234	3.031431
H	6.490919	-5.788475	1.854307
H	6.487583	-4.732790	0.432404
H	6.563153	-4.031455	2.054438
H	4.381517	-6.919428	1.084766
H	2.932740	-5.980449	0.691472
H	4.355196	-5.905677	-0.364839
H	4.495643	2.737816	3.172061
H	3.006081	1.842614	2.860252
H	4.441839	0.996363	3.465439
H	6.458311	2.582989	1.645524
H	6.544120	0.838752	1.933030

3c-*trans*

E(ωB97XD/6-311+G**)= -5049.607038

C	-0.047090	0.138388	0.578332
C	0.244871	-0.230585	1.920214
C	1.649398	-0.401450	2.030410
C	2.238651	-0.139595	0.752562
C	1.173879	0.197376	-0.141502
Fe	0.917606	-1.679604	0.621182
C	-0.417339	-3.192721	0.235598
C	0.410327	-3.528544	1.341538
C	1.760956	-3.510512	0.904159
C	1.783988	-3.174858	-0.490432
C	0.421850	-2.984243	-0.887552
P	3.115968	-3.146029	-1.751136
C	4.478439	-4.283831	-1.100440
C	3.820602	-5.653400	-0.866508
P	4.015986	-0.409509	0.450992
C	4.666798	1.362592	0.287441
C	6.154549	1.277455	-0.078695
Se	3.977794	-1.036256	-1.763928
C	5.227246	-3.825286	0.151403
C	5.483375	-4.406436	-2.257300
C	4.522046	1.967678	1.693021
C	3.922049	2.241888	-0.719784
H	0.103053	-2.704152	-1.881064
H	-1.492013	-3.090494	0.252001
H	0.075603	-3.722390	2.349743
H	2.618003	-3.695740	1.530891
H	1.285705	0.402581	-1.195262
H	-1.032243	0.305801	0.168595
H	-0.477088	-0.389936	2.707208
H	2.186987	-0.719725	2.911900
H	6.281236	-5.101720	-1.974880
H	5.008336	-4.784979	-3.166579
H	5.946429	-3.442559	-2.486845
H	4.599499	-6.399443	-0.675783
H	3.144679	-5.645121	-0.009299
H	3.252879	-5.982878	-1.742530
H	5.974254	-4.581510	0.420294
H	5.751394	-2.883988	-0.023756
H	4.573011	-3.683798	1.011528
H	4.949340	2.976258	1.700511
H	3.472687	2.046677	1.989878
H	5.049266	1.374198	2.446196
H	4.351306	3.250317	-0.711484
H	4.000513	1.857947	-1.740168
H	2.862967	2.328235	-0.467240
H	6.595092	2.280245	-0.061932
H	6.706332	0.655093	0.632163
H	6.304131	0.862976	-1.079353

TS between **3c-*trans*** and **3c**

E(ωB97XD/6-311+G**)= -5049.558218

C	0.398934	0.058929	-0.161004
C	0.175871	0.149234	1.237802
C	1.429333	-0.015224	1.909077
C	2.418145	-0.230066	0.897715
C	1.784960	-0.180098	-0.372263
Fe	0.968932	-1.677737	0.774437
C	-0.015728	-3.208879	-0.172991
C	-0.470024	-3.096636	1.166219
C	0.650129	-3.265005	2.036157
C	1.802304	-3.485420	1.207879
C	1.383101	-3.470158	-0.146806
P	0.685719	-3.042415	3.812928
C	1.189408	-4.416273	4.979563
C	2.280919	-3.898291	5.922068
P	1.861826	0.015670	3.688441
C	1.506024	1.819188	4.146033
C	2.555343	2.656373	3.398789
Se	0.013909	-1.101130	4.543842
C	1.729729	-5.554940	4.106774
C	-0.012865	-4.908608	5.795388
C	1.743813	1.935376	5.658700
C	0.106810	2.334886	3.799593
H	2.811448	-3.614411	1.568143
H	2.021555	-3.590042	-1.009373
H	-0.621112	-3.095960	-1.060171
H	-1.476426	-2.878935	1.490552
H	3.464830	-0.422464	1.083183
H	2.265071	-0.331158	-1.327630
H	-0.359131	0.114890	-0.928187
H	-0.780243	0.276031	1.722213
H	0.304516	-5.703431	6.481522
H	-0.439896	-4.098359	6.391683
H	-0.795774	-5.301838	5.143682
H	2.556387	-4.685921	6.633241
H	3.170818	-3.595737	5.366841
H	1.930809	-3.035807	6.495573
H	1.994362	-6.396805	4.755158
H	0.985202	-5.904210	3.386938
H	2.625345	-5.253929	3.558906
H	0.003747	3.368709	4.149572
H	-0.066878	2.331835	2.722049
H	-0.682212	1.746398	4.276531
H	1.668581	2.983857	5.967062
H	1.004844	1.366373	6.230595
H	2.737737	1.572632	5.936604
H	2.469281	3.706019	3.701803
H	3.573680	2.326036	3.625405
H	2.410992	2.605787	2.316085

2c-*cis*

E(ωB97XD/6-311+G**)= -5049.591638

C	0.041572	0.054676	-0.005513
C	0.026512	0.045190	1.413619
C	1.371396	0.025682	1.870414
C	2.222714	0.032470	0.737029
C	1.408863	0.033759	-0.440724
Fe	1.007022	-1.609053	0.655259
C	0.414453	-3.140181	-0.540372
C	1.843093	-3.009981	-0.515631
C	2.247894	-3.208799	0.849515
C	1.093476	-3.431516	1.640646
C	-0.038908	-3.389410	0.782455
P	3.061018	-2.260717	-1.656117
C	3.208571	-3.416946	-3.134519
C	3.875839	-4.665229	-2.524597
P	2.150750	-0.229549	-2.098120
Se	3.778657	1.089717	-2.477985
C	0.769322	-0.000702	-3.372218
C	-0.355546	-1.031469	-3.240294
C	0.215369	1.422830	-3.191252
C	1.384863	-0.103130	-4.773289
C	4.171793	-2.769522	-4.138315
C	1.914400	-3.849314	-3.822341
H	3.302310	0.006057	0.738097
H	1.694024	-0.005769	2.900013
H	-0.858284	0.041709	2.032123
H	-0.838101	0.060782	-0.626088
H	3.264357	-3.132104	1.206423
H	1.076340	-3.583119	2.709349
H	-1.068861	-3.507024	1.084988
H	-0.219619	-3.027292	-1.404329
H	4.093164	-5.382334	-3.323453
H	4.817340	-4.417867	-2.027173
H	3.221658	-5.158575	-1.799762
H	4.373447	-3.472133	-4.954433
H	3.765743	-1.853217	-4.572046
H	5.123331	-2.510358	-3.667508
H	2.135422	-4.641546	-4.546729
H	1.191155	-4.249488	-3.106894
H	1.449360	-3.032177	-4.372927
H	-1.082511	-0.857698	-4.040256
H	0.013568	-2.050659	-3.348070
H	-0.887688	-0.962433	-2.293146
H	0.610358	0.123993	-5.512628
H	2.205094	0.605645	-4.897158
H	1.762960	-1.104131	-4.986930
H	-0.549937	1.596117	-3.955093
H	-0.242767	1.577376	-2.213228
H	1.003989	2.166772	-3.318668

TS between **2c**-***cis*** and **3c**-***trans***

E(ωB97XD/6-311+G**)= -5049.539451

C	0.002361	-0.003122	0.047803
C	0.002199	-0.018469	1.469862
C	1.351118	-0.022701	1.914172
C	2.200479	-0.023381	0.765280
C	1.353474	-0.002188	-0.391353
Fe	1.024031	-1.656693	0.739958
C	2.403743	-3.128612	0.597672
C	1.701710	-3.229937	1.846568
C	0.321362	-3.405050	1.580968
C	0.142107	-3.435152	0.171889
C	1.412153	-3.267081	-0.436529
P	4.219342	-2.915299	0.507647
C	4.631594	-2.801574	-1.353603
C	3.899040	-1.825517	-2.265753
P	4.000669	-0.316454	0.718422
Se	4.544227	-1.270239	2.646780
C	4.737958	1.422635	0.661561
C	6.262692	1.293896	0.613182
C	4.227923	2.106876	-0.611607
C	4.306415	2.202933	1.903199
C	4.438991	-4.255233	-1.830077
C	6.129359	-2.467073	-1.380859
H	1.696261	-0.092811	2.934749
H	-0.872123	-0.051644	2.102602
H	-0.869425	-0.016698	-0.589253
H	1.688523	-0.022709	-1.417130
H	2.151780	-3.113116	2.820410
H	-0.459473	-3.474766	2.323268
H	-0.796457	-3.542117	-0.351052
H	1.581211	-3.238614	-1.499086
H	4.887670	-4.368724	-2.822614
H	4.935256	-4.968584	-1.164124
H	3.386683	-4.536715	-1.901517
H	6.504122	-2.518406	-2.408662
H	6.311925	-1.455118	-1.006741
H	6.712233	-3.163157	-0.770941
H	4.278816	-1.928091	-3.288571
H	2.821773	-1.981160	-2.291314
H	4.081958	-0.796468	-1.946529
H	4.667737	3.106948	-0.688231
H	4.512903	1.551111	-1.510518
H	3.141027	2.220218	-0.604256
H	6.712324	2.291893	0.578838
H	6.648883	0.774773	1.493648
H	6.590527	0.749798	-0.277747
H	4.795300	3.183471	1.907925
H	3.225188	2.362129	1.920788
H	4.594841	1.682221	2.821790

2d-cis

E(ωB97XD/6-311+G**)=-2916.106023

C	0.041439	0.054674	-0.009922
C	0.018031	0.041771	1.409083
C	1.359838	0.018980	1.873300
C	2.217621	0.028028	0.744727
C	1.411268	0.032433	-0.438410
Fe	1.003087	-1.610828	0.651504
C	0.426612	-3.145663	-0.554153
C	1.853666	-3.010235	-0.513095
C	2.245054	-3.209776	0.855704
C	1.082090	-3.436927	1.633492
C	-0.041040	-3.397087	0.762899
P	3.074637	-2.259709	-1.650035
C	3.212538	-3.417275	-3.129901
C	3.880694	-4.664946	-2.519455
P	2.155675	-0.229863	-2.097931
Te	3.976913	1.233791	-2.502562
C	0.764962	-0.003644	-3.370352
C	-0.361466	-1.032506	-3.230407
C	0.211843	1.420594	-3.190511
C	1.365801	-0.111657	-4.777604
C	4.168478	-2.775823	-4.143664
C	1.911258	-3.847539	-3.805027
H	3.297347	0.003864	0.754046
H	1.675686	-0.015804	2.904871
H	-0.870800	0.036027	2.021741
H	-0.834090	0.063732	-0.635908
H	3.256704	-3.129869	1.225551
H	1.052305	-3.590699	2.701598
H	-1.073991	-3.518002	1.053358
H	-0.194970	-3.033246	-1.426953
H	4.084053	-5.388428	-3.316049
H	4.829956	-4.420554	-2.035539
H	3.232407	-5.149692	-1.783581
H	4.361357	-3.482238	-4.958624
H	3.760864	-1.860407	-4.578075
H	5.124714	-2.517937	-3.681896
H	2.121660	-4.646039	-4.525503
H	1.192117	-4.237812	-3.080382
H	1.448512	-3.030316	-4.357305
H	-1.114796	-0.827596	-3.998032
H	-0.002393	-2.048623	-3.388009
H	-0.858698	-0.995967	-2.263315
H	0.576732	0.095546	-5.507648
H	2.171212	0.609836	-4.924740
H	1.756026	-1.108967	-4.987315
H	-0.563106	1.587174	-3.946182
H	-0.235194	1.582344	-2.208601
H	0.996366	2.166212	-3.333064

TS between **2d** and **2d-*cis***

E(ωB97XD/6-311+G**)=-2916.075009

C	1.834353	1.376040	0.093464
C	2.682640	0.945179	1.168186
C	3.954081	0.622547	0.629729
C	3.923483	0.878615	-0.770654
C	2.627274	1.342564	-1.105711
Fe	2.454210	-0.495059	-0.263277
C	2.739511	-2.539500	-0.175944
C	1.665346	-2.131194	0.656156
C	0.690974	-1.467594	-0.163241
C	1.182266	-1.494719	-1.506549
C	2.443215	-2.142209	-1.507817
P	-0.819037	-0.523370	0.287530
C	-1.229722	-0.958851	2.089293
C	-1.411108	-2.480727	2.187874
P	0.036035	1.431821	0.085705
C	-0.870424	3.067747	-0.025380
C	0.101598	4.135457	0.490664
C	-1.242073	3.348548	-1.488633
C	-2.137461	3.046319	0.832994
Te	-2.647830	-0.906936	-1.171815
C	-2.550480	-0.272979	2.457299
C	-0.149012	-0.456549	3.051930
H	2.261198	1.572837	-2.094756
H	4.737451	0.718541	-1.461829
H	4.796904	0.234979	1.182338
H	2.375999	0.834179	2.196490
H	0.678013	-1.054158	-2.353639
H	3.074673	-2.289931	-2.370714
H	3.632124	-3.047939	0.155884
H	1.618648	-2.285988	1.720493
H	-1.713486	-2.732560	3.209541
H	-2.192278	-2.826109	1.506822
H	-0.496461	-3.031075	1.962295
H	-2.805757	-0.538997	3.488106
H	-2.464554	0.813999	2.405827
H	-3.365875	-0.587061	1.803690
H	-0.452866	-0.693307	4.076553
H	0.826555	-0.910960	2.882688
H	-0.033142	0.627346	2.971755
H	-0.364180	5.120049	0.374779
H	0.336890	3.985636	1.546993
H	1.038723	4.140974	-0.072659
H	-2.658785	4.003121	0.717949
H	-2.822901	2.254554	0.519037
H	-1.899224	2.912488	1.890651
H	-1.755472	4.314898	-1.561006
H	-0.351803	3.385830	-2.121081
H	-1.904392	2.571939	-1.877103

2d

E(ωB97XD/6-311+G**)=-2916.122186

C	-0.171090	-0.117643	0.086932
C	-0.129893	-0.098918	1.505133
C	1.245001	-0.021665	1.913888
C	2.035070	0.025231	0.716117
C	1.163175	-0.043619	-0.400928
Fe	0.940335	-1.686225	0.844068
C	-0.064897	-3.330649	1.525929
C	0.379045	-3.581039	0.203707
C	1.793772	-3.443015	0.173613
C	2.233368	-3.098593	1.479336
C	1.076027	-3.018794	2.331429
P	0.959947	-2.276886	4.001801
C	2.101963	-3.323813	5.086911
C	3.533219	-3.366614	4.535553
P	2.010197	-0.312437	3.551814
C	1.336410	1.087454	4.630278
C	1.514914	0.719363	6.105662
C	1.523430	-4.745838	5.102970
C	2.122183	-2.753015	6.507628
C	2.258255	2.270104	4.280910
C	-0.114014	1.494805	4.363486
H	-0.975778	-0.200398	2.168400
H	-1.063090	-0.199258	-0.515987
H	1.461899	-0.055712	-1.438274
H	3.114459	0.050239	0.685181
H	-1.084988	-3.315718	1.879358
H	-0.254091	-3.817897	-0.637881
H	2.426008	-3.555620	-0.694359
H	3.249913	-2.873512	1.759687
H	2.192260	-5.387437	5.685907
H	0.532304	-4.771816	5.559256
H	1.450625	-5.162341	4.094361
H	2.654993	-3.452633	7.159189
H	2.648318	-1.797101	6.548794
H	1.113357	-2.618524	6.904269
H	4.169498	-3.868885	5.271221
H	3.590475	-3.942452	3.611008
H	3.943702	-2.369402	4.361777
H	1.943751	3.150489	4.851772
H	3.302472	2.058417	4.526950
H	2.199467	2.527136	3.218584
H	1.277126	1.590996	6.724559
H	0.844317	-0.091340	6.400848
H	2.546016	0.428927	6.329411
H	-0.385116	2.304173	5.051008
H	-0.241608	1.868222	3.345593
H	-0.806625	0.668102	4.528125
Te	-1.277983	-2.263691	4.815781

3d

E(ωB97XD/6-311+G**)= -2916.126764

C	-0.042272	0.045260	0.169719
C	-0.024563	0.019258	1.589672
C	1.337608	-0.007072	2.023359
C	2.153067	0.010819	0.845448
C	1.303727	0.041037	-0.290562
Fe	0.956883	1.673249	0.910422
C	-0.076437	3.309253	0.237337
C	-0.058657	3.276790	1.657157
C	1.302855	3.313645	2.092153
C	2.117995	3.361610	0.914862
C	1.268979	3.360695	-0.221791
P	2.056598	3.292990	3.758533
Te	0.652759	1.586893	4.925400
P	2.091460	-0.039695	3.689495
C	1.548607	-1.726908	4.358411
C	0.040501	-1.979711	4.400513
C	2.138942	-1.855652	5.769297
C	2.210198	-2.761305	3.432851
C	1.479022	4.939354	4.496613
C	-0.034009	5.158594	4.548966
C	2.118151	6.024786	3.614744
C	2.067571	5.021961	5.911696
H	3.233121	0.026537	0.839887
H	1.623110	0.089842	-1.320909
H	-0.923708	0.099174	-0.452065
H	-0.886787	0.067967	2.237446
H	3.198139	3.368736	0.909120
H	1.588865	3.361293	-1.253137
H	-0.956822	3.262702	-0.386526
H	-0.919391	3.183259	2.301983
H	-0.149500	-2.986493	4.790448
H	-0.476101	-1.272324	5.055928
H	-0.407373	-1.918486	3.406918
H	1.951486	-2.862707	6.157398
H	3.219915	-1.689676	5.769132
H	1.688696	-1.140510	6.463744
H	2.032139	-3.768232	3.825912
H	1.798558	-2.715641	2.420997
H	3.292401	-2.612742	3.370029
H	-0.244717	6.144142	4.980310
H	-0.481248	5.129298	3.553636
H	-0.535229	4.413984	5.174407
H	1.859396	6.007965	6.341091
H	1.632878	4.269457	6.575913
H	3.151776	4.878774	5.904808
H	1.919320	7.010641	4.049096
H	3.203183	5.901659	3.546007
H	1.706845	6.012400	2.601801

3d-*trans*

E(ωB97XD/6-311+G**)= -2916.123537

C	1.143120	1.419495	0.326460
C	1.072953	2.023537	-0.968268
C	0.679433	3.377390	-0.808416
C	0.499747	3.626598	0.579876
C	0.784301	2.425449	1.279699
Fe	-0.744716	2.111800	-0.037755
C	-2.031167	0.511939	-0.299830
C	-2.206042	1.479043	-1.338898
C	-2.567066	2.720761	-0.756338
C	-2.622066	2.540676	0.651985
C	-2.283553	1.190955	0.937085
P	-1.824141	-1.266385	-0.711484
C	-2.306910	-2.240920	0.839179
C	-1.424261	-2.055352	2.074760
P	1.506635	-0.285786	0.857744
C	3.379473	-0.408393	0.575352
C	4.002242	0.566871	1.587336
Te	0.603087	-1.605713	-1.070784
C	-2.261430	-3.719229	0.420597
C	-3.766467	-1.869090	1.151699
C	3.860037	-0.046160	-0.831362
C	3.801141	-1.841212	0.927326
H	-2.069339	1.285794	-2.393038
H	-2.738204	3.645715	-1.286382
H	-2.843093	3.303070	1.384013
H	-2.218703	0.764109	1.924428
H	1.242900	1.516827	-1.906210
H	0.505342	4.084805	-1.605729
H	0.166476	4.554386	1.020565
H	0.708736	2.273124	2.346541
H	-2.589892	-4.342980	1.258920
H	-2.919525	-3.919370	-0.429466
H	-1.247166	-4.028393	0.152749
H	-4.149734	-2.544399	1.924356
H	-3.867929	-0.846899	1.519551
H	-4.405356	-1.975695	0.269806
H	-1.852136	-2.616688	2.913829
H	-0.415629	-2.433764	1.897649
H	-1.328938	-1.013402	2.380515
H	5.094146	0.488810	1.542081
H	3.730424	1.602814	1.367555
H	3.691940	0.339869	2.611656
H	4.951988	-0.129553	-0.878812
H	3.447590	-0.713579	-1.593280
H	3.592437	0.979484	-1.093870
H	4.893609	-1.920199	0.909212
H	3.459552	-2.124638	1.927182
H	3.404307	-2.570552	0.215202

TS between **3d-*trans*** and **2d-*cis***

E(ωB97XD/6-311+G**)= -2916.0492854

C	-2.070055	-2.269012	1.770644
C	-1.734945	-3.000471	0.587299
C	-0.555762	-2.420678	0.023216
C	-0.162621	-1.311259	0.844143
C	-1.101350	-1.228436	1.935044
Fe	-2.033788	-0.997910	0.136014
C	-1.844841	0.824113	-0.744672
C	-2.140031	-0.183526	-1.738032
C	-3.391476	-0.786167	-1.417560
C	-3.897573	-0.161573	-0.234463
C	-2.952598	0.821265	0.189338
P	-0.321879	1.869307	-0.845021
C	-0.334537	3.009537	0.736123
C	-0.577841	2.392427	2.112948
P	1.172778	-0.066272	0.523919
Te	1.743176	-0.251483	-1.938327
C	2.666434	-0.750432	1.560513
C	3.849937	0.201191	1.342854
C	2.238137	-0.752558	3.036224
C	3.004215	-2.166821	1.086578
C	-1.430786	4.048104	0.394878
C	1.047399	3.687277	0.723012
H	-0.056940	-2.726502	-0.882696
H	-2.271925	-3.847114	0.191667
H	-2.901508	-2.468578	2.426986
H	-1.078902	-0.504772	2.733268
H	-1.499578	-0.445322	-2.564755
H	-3.871796	-1.574058	-1.973990
H	-4.831431	-0.385641	0.254775
H	-3.079313	1.466228	1.040408
H	-1.398715	4.852918	1.139743
H	-1.269166	4.500774	-0.590153
H	-2.432775	3.608795	0.411127
H	1.086452	4.456846	1.503921
H	1.838650	2.955764	0.922954
H	1.255353	4.165033	-0.240303
H	-0.555305	3.188004	2.869296
H	-1.538550	1.881540	2.192330
H	0.211474	1.674415	2.354463
H	3.072279	-1.109736	3.653850
H	1.974526	0.254880	3.378531
H	1.385171	-1.417385	3.205737
H	4.712371	-0.150478	1.922866
H	4.140213	0.241111	0.287547
H	3.609602	1.218511	1.672858
H	3.895006	-2.528084	1.617232
H	2.180363	-2.860017	1.285016
H	3.224051	-2.187744	0.011976

Calculations at M06-2X/6-311+G** level of theory

3a

E(M06-2X/6-311+G**)= -2723.006582

C	-0.003734	-0.072615	-0.060948
C	0.098549	-0.037437	1.351528
C	1.485016	-0.051390	1.704500
C	2.222488	-0.094630	0.479437
C	1.309814	-0.103851	-0.601355
Fe	1.037632	1.681188	0.567956
C	-0.040572	3.437311	0.011820
C	0.063073	3.345795	1.421666
C	1.449111	3.374193	1.775489
C	2.184946	3.483608	0.553568
C	1.271784	3.518454	-0.526288
P	2.337898	3.084192	3.350187
O	1.652194	1.619848	3.838820
P	2.368146	0.191648	3.290273
C	1.533692	-0.978567	4.482975
C	0.015048	-0.824826	4.575995
C	2.160327	-0.684803	5.853221
C	1.899217	-2.397542	4.033985
C	1.479958	4.186338	4.590159
C	-0.035025	3.996934	4.676806
C	1.815111	5.630022	4.200121
C	2.113833	3.849580	5.947117
H	3.300416	-0.078557	0.409867
H	1.564439	-0.108058	-1.650179
H	-0.921594	-0.041231	-0.628805
H	-0.729364	0.060385	2.034875
H	3.262941	3.493039	0.483830
H	1.525783	3.571452	-1.573932
H	-0.957831	3.410235	-0.557229
H	-0.762294	3.202392	2.100023
H	-0.353198	-1.376433	5.447692
H	-0.269393	0.223965	4.690999
H	-0.479261	-1.238147	3.696308
H	1.788209	-1.403838	6.589716
H	3.250404	-0.762408	5.821247
H	1.896599	0.319739	6.192206
H	1.490165	-3.122814	4.744425
H	1.486031	-2.620613	3.046363
H	2.982922	-2.539890	3.994708
H	-0.413979	4.504071	5.570640
H	-0.538734	4.435865	3.815047
H	-0.297235	2.938521	4.748261
H	1.727348	4.529658	6.712643
H	1.871563	2.826596	6.244396
H	3.202012	3.951338	5.918350
H	1.391437	6.316574	4.939860

H	2.895541	5.796703	4.166805
H	1.396502	5.884877	3.222523

3a-*trans*

E(M06-2X/6-311+G**)= -2723.006529

C	-0.036903	0.050275	-0.161836
C	-0.191106	0.214066	1.241928
C	1.101506	0.248830	1.820588
C	2.069232	0.104938	0.779038
C	1.348244	-0.010463	-0.450017
Fe	0.880339	-1.612331	0.844237
C	-0.086673	-3.540311	0.873791
C	0.557472	-3.305239	2.118789
C	1.939401	-3.116505	1.875508
C	2.170423	-3.223022	0.464800
C	0.900522	-3.496364	-0.137979
P	3.616697	-2.881103	-0.611206
C	4.976921	-3.929215	0.137939
C	4.584060	-5.388654	-0.117791
P	3.827619	-0.288349	1.098072
C	4.718791	1.184257	0.367033
C	6.212147	0.836966	0.376543
O	4.117946	-1.341746	-0.179831
C	5.227077	-3.701509	1.628147
C	6.247689	-3.583576	-0.650012
C	4.443614	2.363808	1.305758
C	4.271361	1.517657	-1.055184
H	0.734827	-3.597952	-1.200627
H	-1.143105	-3.704176	0.726300
H	0.073297	-3.245819	3.082007
H	2.674107	-2.849790	2.619553
H	1.793122	-0.191018	-1.417822
H	-0.837356	-0.042770	-0.880262
H	-1.127679	0.274703	1.774857
H	1.326853	0.306629	2.875717
H	7.070923	-4.227214	-0.324254
H	6.105584	-3.729175	-1.724312
H	6.539433	-2.544181	-0.480617
H	5.389505	-6.049197	0.218597
H	3.675186	-5.657501	0.427582
H	4.416642	-5.579575	-1.181847
H	6.139461	-4.228814	1.928240
H	5.363738	-2.641492	1.858403
H	4.408949	-4.092922	2.233912
H	4.988815	3.245569	0.954928
H	3.379026	2.613634	1.329173
H	4.771752	2.151022	2.327424
H	4.903890	2.315307	-1.459405
H	4.362596	0.645600	-1.706707
H	3.237185	1.867148	-1.075119

H	6.795912	1.700587	0.042571
H	6.554257	0.564200	1.379260
H	6.418153	0.003556	-0.299006

2a

E(M06-2X/6-311+G**)= -2722.985377

C	0.050782	0.159799	0.008071
C	0.072412	0.198010	1.423867
C	1.427978	0.163831	1.845379
C	2.244786	0.111350	0.691563
C	1.402062	0.091517	-0.463043
Fe	0.998092	-1.601444	0.683286
C	0.426660	-3.203660	-0.563039
C	1.850795	-3.066647	-0.509524
C	2.232200	-3.303610	0.851950
C	1.067937	-3.552806	1.615943
C	-0.048765	-3.490747	0.739924
P	3.055867	-2.266765	-1.634011
C	3.236592	-3.414495	-3.113524
C	3.941550	-4.654099	-2.536752
P	2.115693	-0.256978	-2.115243
O	3.256259	0.647254	-2.473472
C	0.746185	-0.026937	-3.366928
C	-0.420324	-1.008938	-3.260311
C	0.258831	1.421792	-3.180304
C	1.390260	-0.125677	-4.757117
C	4.169439	-2.717972	-4.111396
C	1.943288	-3.864462	-3.789574
H	3.322906	0.045942	0.665507
H	1.776082	0.164054	2.866840
H	-0.794561	0.235898	2.065417
H	-0.844588	0.153018	-0.590778
H	3.241911	-3.226587	1.227759
H	1.031827	-3.741018	2.677980
H	-1.083064	-3.620756	1.020623
H	-0.189132	-3.053424	-1.435550
H	4.165261	-5.350743	-3.351124
H	4.881223	-4.389749	-2.046326
H	3.306197	-5.171477	-1.812658
H	4.347438	-3.377438	-4.967050
H	3.750792	-1.780064	-4.482759
H	5.132645	-2.486457	-3.651171
H	2.170484	-4.641524	-4.527896
H	1.242156	-4.287949	-3.065920
H	1.454541	-3.047091	-4.317617
H	-1.151351	-0.771499	-4.039972
H	-0.097997	-2.039269	-3.414875
H	-0.933220	-0.954913	-2.300138
H	0.654000	0.176243	-5.508182
H	2.256627	0.534011	-4.829104

H	1.707086	-1.141714	-4.995541
H	-0.450573	1.656544	-3.979984
H	-0.242730	1.577923	-2.225038
H	1.096435	2.119055	-3.246063

2a-trans

E(M06-2X/6-311+G**)= -2723.002509

C	0.051334	0.072152	-0.028380
C	0.012469	0.062614	1.398392
C	1.344581	0.101869	1.878266
C	2.217400	0.148171	0.757585
C	1.427700	0.134655	-0.419799
Fe	1.012725	-1.632586	0.628627
C	1.880760	-3.610009	0.698744
C	0.719192	-3.562924	1.517060
C	-0.380277	-3.186697	0.706748
C	0.092479	-2.991810	-0.632846
C	1.495919	-3.270571	-0.621497
P	-0.725436	-2.205800	-2.081850
C	-2.248227	-3.301309	-2.342586
C	-3.037366	-3.650099	-1.077646
P	-1.435521	-0.348837	-0.998399
O	-2.635582	-0.535360	-0.112359
C	-1.647178	1.074815	-2.172199
C	-2.836563	0.811484	-3.097590
C	-0.385157	1.332320	-2.999543
C	-1.953379	2.287338	-1.279191
C	-3.180872	-2.619684	-3.345459
C	-1.683802	-4.588244	-2.963623
H	-1.383488	-2.983719	1.050166
H	0.683863	-3.752813	2.579089
H	2.879689	-3.853544	1.026378
H	2.150416	-3.174893	-1.475359
H	-0.893208	-0.030436	1.979262
H	1.644613	0.084484	2.914719
H	3.295926	0.169421	0.792406
H	1.803812	0.104143	-1.431231
H	-2.102648	3.166042	-1.913984
H	-2.858528	2.120316	-0.692324
H	-1.128916	2.498872	-0.593462
H	-3.061355	1.725727	-3.655201
H	-2.613020	0.026863	-3.822956
H	-3.726645	0.531578	-2.529370
H	-0.599853	2.116342	-3.732407
H	0.438123	1.682670	-2.375233
H	-0.064032	0.441302	-3.545664
H	-2.505721	-5.290713	-3.136715
H	-1.192260	-4.393001	-3.920105
H	-0.964894	-5.070719	-2.295684
H	-3.942177	-3.336761	-3.668612

H	-3.695664	-1.773577	-2.887112
H	-2.646115	-2.275973	-4.235719
H	-3.943198	-4.191715	-1.372545
H	-2.462373	-4.308514	-0.425381
H	-3.330301	-2.755440	-0.524510

3b

E(M06-2X/6-311+G**)= -3045.978359

C	-0.055091	-0.070425	0.070664
C	0.015595	-0.052695	1.485553
C	1.391091	-0.073119	1.866432
C	2.160361	-0.098959	0.662011
C	1.270743	-0.096410	-0.439616
Fe	0.974568	1.677117	0.736002
C	-0.091515	3.428934	0.143231
C	-0.019866	3.354047	1.556200
C	1.355070	3.387279	1.938192
C	2.123118	3.479022	0.736209
C	1.233271	3.503629	-0.364961
P	2.161599	3.207509	3.570679
S	0.993941	1.600787	4.426629
P	2.194416	0.055582	3.505312
C	1.554352	-1.463815	4.419734
C	0.034422	-1.618894	4.466840
C	2.115679	-1.386828	5.844954
C	2.170399	-2.663331	3.686503
C	1.490362	4.674023	4.546982
C	-0.032466	4.794865	4.600336
C	2.080347	5.915497	3.863876
C	2.054350	4.550209	5.967848
H	3.239499	-0.082183	0.616889
H	1.549580	-0.084611	-1.482268
H	-0.959180	-0.028133	-0.518107
H	-0.819262	0.040164	2.163508
H	3.202352	3.486589	0.690898
H	1.511844	3.540865	-1.407086
H	-0.994783	3.392282	-0.447177
H	-0.852318	3.215822	2.229364
H	-0.218466	-2.492288	5.077885
H	-0.450928	-0.747183	4.912797
H	-0.379212	-1.781174	3.470548
H	1.876319	-2.310541	6.381177
H	3.202386	-1.268411	5.839614
H	1.683615	-0.549757	6.398110
H	1.900378	-3.585437	4.211010
H	1.800326	-2.735747	2.660366
H	3.261649	-2.599197	3.657238
H	-0.303260	5.636777	5.246866
H	-0.450233	4.989304	3.611553
H	-0.498930	3.895437	5.009926

H	1.796041	5.445796	6.541672
H	1.640456	3.682143	6.486017
H	3.143304	4.455030	5.957688
H	1.791271	6.809292	4.425895
H	3.172687	5.875735	3.832126
H	1.708055	6.022272	2.841548

3b-*trans*

E(M06-2X/6-311+G**)= -3045.973693

C	0.349267	-0.178343	-0.125071
C	0.491787	-0.198201	1.283786
C	1.886029	-0.132354	1.599142
C	2.586164	-0.074304	0.354627
C	1.644575	-0.091338	-0.701111
Fe	1.288922	1.616263	0.544155
C	0.515592	3.424591	-0.318641
C	-0.003502	3.228759	0.984418
C	1.088246	3.197901	1.905920
C	2.283367	3.389265	1.148661
C	1.928899	3.521994	-0.214927
P	0.867452	2.669636	3.642302
S	2.808604	1.773952	4.000913
P	2.831972	-0.208021	3.169627
C	1.787401	-1.255792	4.340007
C	0.494372	-0.627051	4.856472
C	2.705781	-1.536914	5.538673
C	1.496129	-2.572937	3.608634
C	1.133448	4.277173	4.597722
C	-0.076353	5.154624	4.248808
C	2.422504	5.020000	4.247813
C	1.103972	3.929576	6.090083
H	3.659509	-0.002604	0.256161
H	1.869366	-0.032148	-1.754928
H	-0.586489	-0.188350	-0.663686
H	-0.322095	-0.214968	1.991038
H	3.284584	3.357540	1.551481
H	2.617174	3.638300	-1.038731
H	-0.058189	3.459199	-1.232288
H	-1.040178	3.065929	1.242029
H	2.172738	-2.155831	6.267525
H	3.610412	-2.067797	5.233030
H	3.000985	-0.608407	6.035194
H	1.092134	-3.297475	4.322950
H	0.768636	-2.440243	2.806090
H	2.405101	-3.002739	3.176444
H	-0.027674	-1.355352	5.487363
H	0.704367	0.255726	5.464387
H	-0.181334	-0.326177	4.056738
H	-0.031132	6.079817	4.832444
H	-0.081919	5.423540	3.189276

H	-1.019195	4.652922	4.485066
H	2.468404	5.953913	4.818754
H	3.309276	4.432707	4.495309
H	2.458908	5.271411	3.185589
H	1.141854	4.849825	6.681865
H	0.187760	3.394071	6.354906
H	1.957942	3.309244	6.371995

2b-cis

E(M06-2X/6-311+G**)= -3045.955555

C	0.052628	0.160015	-0.001038
C	0.059366	0.191220	1.415302
C	1.409423	0.146834	1.851514
C	2.239065	0.098119	0.707005
C	1.409463	0.088503	-0.455476
Fe	0.983148	-1.610721	0.677384
C	0.410933	-3.215941	-0.569567
C	1.834569	-3.076441	-0.515901
C	2.215966	-3.311770	0.846871
C	1.051930	-3.562681	1.610145
C	-0.064156	-3.502161	0.733592
P	3.058246	-2.278156	-1.624243
C	3.230563	-3.415962	-3.114099
C	3.922169	-4.659427	-2.526410
P	2.140057	-0.244397	-2.108023
S	3.671667	0.933961	-2.514171
C	0.754633	-0.008807	-3.367893
C	-0.384136	-1.020407	-3.218817
C	0.233919	1.426957	-3.188117
C	1.353146	-0.122061	-4.774845
C	4.176669	-2.734113	-4.109435
C	1.939799	-3.859230	-3.798588
H	3.317645	0.035154	0.688957
H	1.746610	0.139838	2.876544
H	-0.815031	0.233732	2.046403
H	-0.836774	0.167296	-0.607669
H	3.225241	-3.231906	1.223353
H	1.015572	-3.749680	2.672379
H	-1.098546	-3.633095	1.013592
H	-0.206286	-3.071095	-1.441298
H	4.147341	-5.357840	-3.338691
H	4.860524	-4.401419	-2.030032
H	3.277677	-5.172003	-1.807104
H	4.371179	-3.412168	-4.946914
H	3.760618	-1.806097	-4.506959
H	5.130843	-2.486071	-3.638914
H	2.168493	-4.645075	-4.526948
H	1.226398	-4.270301	-3.079770
H	1.465979	-3.042882	-4.341429
H	-1.142595	-0.802724	-3.977368

H	-0.038741	-2.041293	-3.381750
H	-0.868900	-0.977681	-2.244915
H	0.567687	0.102785	-5.502664
H	2.171183	0.585948	-4.911860
H	1.726130	-1.124628	-4.987311
H	-0.524149	1.615522	-3.954782
H	-0.220982	1.593552	-2.211675
H	1.042791	2.148126	-3.316241

2c

E(M06-2X/6-311+G**)= -3045.973213

C	-0.186440	0.007450	0.101058
C	-0.120270	-0.026778	1.514795
C	1.258478	0.037285	1.902087
C	2.025587	0.129319	0.696531
C	1.138584	0.100499	-0.406764
Fe	0.945116	-1.681736	0.799062
C	-0.024193	-3.405435	1.558919
C	0.400269	-3.702080	0.241525
C	1.812312	-3.552893	0.185981
C	2.266378	-3.158258	1.468915
C	1.124378	-3.056731	2.330297
P	1.002010	-2.260343	3.972924
C	2.095116	-3.312375	5.079831
C	3.516079	-3.435632	4.519186
P	2.053843	-0.287817	3.524662
C	1.330668	1.067423	4.632700
C	1.539632	0.684770	6.098451
C	1.441419	-4.699649	5.137099
C	2.144129	-2.706242	6.482859
C	2.191530	2.296330	4.295610
C	-0.140260	1.411834	4.393072
H	-0.951564	-0.168609	2.189419
H	-1.088386	-0.053129	-0.488880
H	1.419253	0.134086	-1.448205
H	3.104506	0.153237	0.651578
H	-1.037416	-3.375442	1.930930
H	-0.240867	-3.981282	-0.580323
H	2.433630	-3.692792	-0.685458
H	3.283160	-2.905123	1.726391
H	2.075737	-5.357148	5.739466
H	0.451937	-4.651082	5.593471
H	1.344341	-5.139092	4.140823
H	2.643434	-3.413601	7.151616
H	2.716211	-1.776875	6.496122
H	1.141582	-2.515816	6.871712
H	4.137053	-3.940512	5.265473
H	3.536208	-4.041992	3.613200
H	3.966614	-2.462107	4.311153
H	1.837598	3.150315	4.882367

H	3.245206	2.129262	4.531911
H	2.110914	2.561375	3.237360
H	1.274690	1.538667	6.730053
H	0.900570	-0.154307	6.382314
H	2.583210	0.431609	6.304913
H	-0.436215	2.181721	5.114191
H	-0.293068	1.819225	3.392696
H	-0.787874	0.546006	4.533915
S	-0.866730	-2.184377	4.623768

3c

E(M06-2X/6-311+G**)= -5049.352549

C	-0.062949	-0.069884	0.101063
C	-0.006438	-0.061028	1.516685
C	1.364774	-0.082457	1.911411
C	2.146482	-0.098213	0.714609
C	1.267934	-0.090521	-0.396012
Fe	0.958283	1.675906	0.784066
C	-0.099486	3.426778	0.173525
C	-0.042184	3.360475	1.587589
C	1.328453	3.394169	1.983452
C	2.109139	3.475808	0.788666
C	1.230459	3.495779	-0.321697
P	2.122995	3.250484	3.626109
Se	0.842554	1.595870	4.588235
P	2.156853	0.009630	3.558954
C	1.558841	-1.570880	4.402233
C	0.044831	-1.775377	4.439227
C	2.119977	-1.550252	5.829074
C	2.209186	-2.714332	3.609801
C	1.492466	4.781875	4.533864
C	-0.025469	4.953003	4.578628
C	2.118454	5.970500	3.789762
C	2.054515	4.713887	5.958875
H	3.225999	-0.079990	0.680284
H	1.557367	-0.070570	-1.435680
H	-0.961107	-0.024637	-0.496490
H	-0.847826	0.024588	2.187813
H	3.188786	3.481574	0.754077
H	1.519791	3.524958	-1.361175
H	-0.996764	3.387572	-0.425776
H	-0.881307	3.229579	2.254235
H	-0.179512	-2.702171	4.978703
H	-0.465870	-0.958434	4.955508
H	-0.369119	-1.868384	3.434103
H	1.907091	-2.506032	6.318271
H	3.202992	-1.402004	5.829288
H	1.667051	-0.754497	6.425042
H	1.972060	-3.668150	4.091853
H	1.835486	-2.750301	2.583172

H	3.297604	-2.613686	3.578887
H	-0.268889	5.851771	5.155926
H	-0.441697	5.078893	3.578036
H	-0.518779	4.104892	5.060407
H	1.821922	5.643929	6.487147
H	1.618559	3.884845	6.521170
H	3.140390	4.588407	5.953425
H	1.861666	6.898373	4.310803
H	3.208723	5.893972	3.755185
H	1.743648	6.041150	2.765339

3c-*trans*

E(M06-2X/6-311+G**)= -5049.347888

C	-0.115902	0.251684	0.500918
C	0.132467	-0.064565	1.863515
C	1.529302	-0.236739	2.021986
C	2.157181	-0.031660	0.754304
C	1.124223	0.278253	-0.180939
Fe	0.844376	-1.667208	0.623346
C	-0.411475	-3.371467	0.300815
C	0.456641	-3.615431	1.398315
C	1.791972	-3.536784	0.931799
C	1.766689	-3.252727	-0.470499
C	0.390642	-3.159106	-0.845082
P	3.067929	-3.156656	-1.760518
C	4.485713	-4.239938	-1.138211
C	3.884270	-5.624090	-0.855541
P	3.925367	-0.416458	0.491488
C	4.689590	1.307686	0.328499
C	6.171308	1.123179	-0.017372
Se	3.880746	-1.015290	-1.735440
C	5.263351	-3.729778	0.074100
C	5.451041	-4.346562	-2.328142
C	4.563002	1.928566	1.727249
C	4.012121	2.219165	-0.694370
H	0.039144	-2.938272	-1.842348
H	-1.489487	-3.336547	0.337892
H	0.155764	-3.791029	2.420122
H	2.671568	-3.638432	1.546868
H	1.271150	0.429496	-1.239677
H	-1.086321	0.407964	0.054249
H	-0.613834	-0.186790	2.633809
H	2.038805	-0.532045	2.927981
H	6.290596	-4.994224	-2.056743
H	4.959373	-4.772031	-3.206207
H	5.854054	-3.366703	-2.597663
H	4.696198	-6.340397	-0.694418
H	3.250679	-5.620621	0.032700
H	3.286411	-5.982163	-1.699074
H	6.034501	-4.462790	0.336468

H	5.759227	-2.783100	-0.149459
H	4.633736	-3.575770	0.949758
H	5.058105	2.904925	1.734596
H	3.516230	2.077962	2.003761
H	5.037984	1.304219	2.489275
H	4.489258	3.205002	-0.671603
H	4.101923	1.830450	-1.711305
H	2.952284	2.352540	-0.466940
H	6.675873	2.094398	0.004996
H	6.668877	0.466955	0.702204
H	6.300652	0.697344	-1.015154

2c

E(M06-2X/6-311+G**)= -5049.326834

C	0.052012	0.157949	0.000003
C	0.057387	0.187984	1.416453
C	1.406895	0.143889	1.854174
C	2.238129	0.097241	0.710945
C	1.409887	0.088023	-0.452293
Fe	0.982411	-1.612720	0.678069
C	0.408752	-3.217039	-0.570398
C	1.832350	-3.077751	-0.517682
C	2.215097	-3.313936	0.844726
C	1.051723	-3.565806	1.608576
C	-0.065149	-3.504209	0.732991
P	3.059448	-2.281076	-1.624628
C	3.227940	-3.419215	-3.115284
C	3.917388	-4.661866	-2.522428
P	2.138317	-0.244444	-2.106305
Se	3.797358	1.026261	-2.530042
C	0.751677	-0.003644	-3.369060
C	-0.382930	-1.019922	-3.214156
C	0.226303	1.429916	-3.189060
C	1.345336	-0.119199	-4.777457
C	4.175842	-2.743293	-4.112747
C	1.936900	-3.863818	-3.798772
H	3.316941	0.037779	0.693120
H	1.743050	0.136403	2.879521
H	-0.817777	0.230180	2.046495
H	-0.836720	0.166538	-0.607446
H	3.224646	-3.234585	1.220601
H	1.016351	-3.754115	2.670610
H	-1.099328	-3.635425	1.013612
H	-0.209174	-3.072406	-1.441580
H	4.142754	-5.362159	-3.332939
H	4.855647	-4.403716	-2.025883
H	3.271518	-5.172102	-1.802739
H	4.373067	-3.427743	-4.944389
H	3.759909	-1.818750	-4.518192
H	5.128444	-2.489750	-3.642005

H	2.166043	-4.653253	-4.523059
H	1.222177	-4.271158	-3.079169
H	1.464473	-3.050037	-4.347040
H	-1.145307	-0.804096	-3.969258
H	-0.034466	-2.039311	-3.379750
H	-0.863203	-0.979221	-2.238120
H	0.553134	0.092015	-5.502064
H	2.154787	0.596857	-4.923128
H	1.728485	-1.119018	-4.984948
H	-0.540512	1.610795	-3.948892
H	-0.219322	1.597816	-2.208565
H	1.030285	2.154473	-3.328296

2c-*trans*

E(M06-2X/6-311+G**)= -5049.344801

C	-0.181730	0.007776	0.096039
C	-0.123577	-0.027191	1.509965
C	1.253156	0.034255	1.904438
C	2.027424	0.126235	0.703209
C	1.146113	0.099853	-0.404616
Fe	0.945767	-1.683414	0.798584
C	-0.026022	-3.413347	1.544891
C	0.411430	-3.704125	0.230796
C	1.823367	-3.550099	0.188050
C	2.264866	-3.157861	1.475722
C	1.114382	-3.063112	2.327839
P	0.989144	-2.273489	3.974713
C	2.095772	-3.318952	5.082466
C	3.513306	-3.434855	4.510215
P	2.038619	-0.294033	3.530330
C	1.326562	1.072172	4.632187
C	1.515953	0.690069	6.100787
C	1.456519	-4.711783	5.153638
C	2.154270	-2.702788	6.480627
C	2.212875	2.284533	4.299435
C	-0.134768	1.443384	4.375825
H	-0.958840	-0.167551	2.180355
H	-1.080610	-0.051121	-0.498720
H	1.432456	0.134246	-1.444483
H	3.106622	0.148356	0.663777
H	-1.042327	-3.388903	1.908922
H	-0.221462	-3.983585	-0.597310
H	2.453010	-3.685773	-0.678086
H	3.278512	-2.902227	1.742482
H	2.111752	-5.362315	5.741124
H	0.477232	-4.673849	5.632040
H	1.341503	-5.152017	4.159544
H	2.668270	-3.401402	7.147420
H	2.718167	-1.768327	6.480582
H	1.154614	-2.519266	6.880025

H	4.143991	-3.925889	5.257553
H	3.531166	-4.051888	3.611573
H	3.953803	-2.459945	4.288188
H	1.869011	3.145591	4.881736
H	3.261435	2.099488	4.544821
H	2.145475	2.549252	3.240137
H	1.250510	1.547447	6.727400
H	0.867533	-0.143605	6.379959
H	2.555422	0.430402	6.319161
H	-0.422663	2.222850	5.089815
H	-0.270110	1.847554	3.371604
H	-0.801307	0.591957	4.515635
Se	-1.028494	-2.221551	4.680384

3d

E(M06-2X/6-311+G**)= -2915.739668

C	-0.062973	-0.067977	0.139738
C	-0.028079	-0.072601	1.556162
C	1.336501	-0.094749	1.972787
C	2.136387	-0.097041	0.787899
C	1.275447	-0.081041	-0.336454
Fe	0.944814	1.674541	0.850397
C	-0.099057	3.423852	0.212221
C	-0.063668	3.370452	1.627634
C	1.300336	3.403432	2.045400
C	2.099510	3.471434	0.861979
C	1.238605	3.484306	-0.262442
P	2.073455	3.313424	3.703645
Te	0.643975	1.589107	4.815647
P	2.108366	-0.057608	3.633658
C	1.561842	-1.710610	4.376628
C	0.055083	-1.963046	4.405720
C	2.130622	-1.767365	5.799060
C	2.238899	-2.783487	3.510507
C	1.492554	4.922399	4.513967
C	-0.019197	5.141503	4.552505
C	2.146588	6.044126	3.693232
C	2.060433	4.932283	5.937859
H	3.216285	-0.077531	0.770856
H	1.581384	-0.048982	-1.371133
H	-0.951890	-0.018466	-0.471138
H	-0.880414	0.000673	2.215209
H	3.179572	3.474967	0.844610
H	1.544698	3.501530	-1.297428
H	-0.987023	3.381380	-0.400567
H	-0.914028	3.252344	2.282717
H	-0.138747	-2.944646	4.852307
H	-0.472879	-1.216996	5.005926
H	-0.369085	-1.965576	3.400410
H	1.943386	-2.756713	6.228479

H	3.209660	-1.593263	5.803822
H	1.663547	-1.022516	6.448236
H	2.037471	-3.771919	3.936038
H	1.855254	-2.769103	2.487187
H	3.323000	-2.645604	3.475251
H	-0.233650	6.099620	5.038892
H	-0.443591	5.176287	3.547874
H	-0.531077	4.360421	5.121251
H	1.852555	5.898895	6.407658
H	1.609312	4.151602	6.555450
H	3.142895	4.780856	5.936055
H	1.924379	7.009707	4.158982
H	3.233356	5.930770	3.653046
H	1.763124	6.063797	2.669930

3d-*trans*

E(M06-2X/6-311+G**)= -2915.735092

C	-0.366278	0.229063	-0.351198
C	-0.635498	0.115228	1.033557
C	0.568521	-0.271168	1.678748
C	1.581813	-0.378893	0.693535
C	1.010343	-0.074613	-0.582214
Fe	0.853839	1.616249	0.736844
C	0.930376	3.556049	-0.131508
C	0.138273	3.615821	1.040225
C	0.947112	3.232434	2.143084
C	2.240175	2.932707	1.649873
C	2.241965	3.126764	0.233571
P	3.690624	2.660405	-0.781144
Te	2.558875	2.003934	-2.918353
P	1.641119	-0.222900	-2.301743
C	3.129900	-1.387481	-2.212572
C	3.511105	-1.660274	-3.674987
C	2.626755	-2.695754	-1.583398
C	4.365156	-0.870491	-1.475413
C	4.398929	4.353668	-1.261996
C	3.404050	5.313556	-1.913448
C	5.588689	4.104366	-2.195255
C	4.902378	4.963645	0.054590
H	-1.070965	0.513619	-1.118984
H	-1.581773	0.308995	1.515087
H	0.703352	-0.418962	2.739567
H	2.612043	-0.626912	0.890434
H	0.592135	3.734880	-1.140986
H	-0.910250	3.869926	1.082135
H	0.623021	3.146865	3.169255
H	3.077423	2.567424	2.226984
H	4.346701	-2.366639	-3.704050
H	2.677109	-2.093224	-4.232598
H	3.827997	-0.743984	-4.180036

H	3.399715	-3.463319	-1.692217
H	2.405305	-2.584540	-0.521358
H	1.723532	-3.057845	-2.082865
H	5.125863	-1.659044	-1.453975
H	4.790971	-0.004826	-1.986839
H	4.156643	-0.573688	-0.448030
H	5.405004	5.913299	-0.155981
H	4.076828	5.162276	0.742517
H	5.619064	4.305690	0.553799
H	3.890873	6.279908	-2.085064
H	3.053193	4.945569	-2.880937
H	2.536080	5.483154	-1.272780
H	6.102144	5.050271	-2.395512
H	6.309623	3.416485	-1.745187
H	5.269874	3.684867	-3.152825

2d

E(M06-2X/6-311+G**)= -2915.712804

C	-0.038920	0.116505	0.003009
C	-0.026858	0.075541	1.418630
C	1.333703	0.064989	1.862613
C	2.148630	0.128445	0.683259
C	1.306053	0.151199	-0.452442
Fe	0.999536	-1.637036	0.729076
C	1.088839	-3.097670	2.217447
C	-0.032696	-3.366200	1.366835
C	0.451527	-3.610479	0.057860
C	1.866316	-3.502405	0.083106
C	2.260553	-3.202899	1.407918
P	1.193121	-2.451025	3.935471
C	-0.534986	-2.628674	4.691683
C	-0.468036	-2.238477	6.172383
P	2.143423	-0.430019	3.432908
C	1.746540	0.934866	4.668854
C	0.283338	1.352031	4.795639
C	2.548257	2.130614	4.121615
C	2.321944	0.521705	6.028196
Te	2.838005	-3.592226	5.212062
C	-1.585744	-1.762004	3.991830
C	-0.913275	-4.116242	4.605725
H	3.267346	-3.039171	1.764856
H	2.528506	-3.618509	-0.760907
H	-0.157395	-3.833399	-0.804764
H	-1.073121	-3.369461	1.642965
H	3.228090	0.089390	0.680764
H	1.627260	0.172855	-1.482475
H	-0.919805	0.105382	-0.621013
H	-0.898764	0.006990	2.048541
H	2.452474	2.968643	4.819152

H	3.609621	1.892026	4.021251
H	2.170192	2.454444	3.148160
H	2.205437	1.349393	6.735585
H	1.821252	-0.356445	6.440760
H	3.384603	0.280464	5.951475
H	0.219764	2.260877	5.404088
H	-0.156414	1.574221	3.820103
H	-0.316276	0.589570	5.290821
H	-2.553093	-1.937257	4.473045
H	-1.357369	-0.700599	4.087039
H	-1.695174	-1.992609	2.933864
H	-1.455433	-2.402622	6.614508
H	0.261000	-2.846247	6.709635
H	-0.210843	-1.187959	6.312844
H	-1.890098	-4.247650	5.081874
H	-0.983262	-4.477216	3.579571
H	-0.184653	-4.732196	5.135726

2d-*trans*

E(M06-2X/6-311+G**)= -2915.730485

C	-0.191345	0.005698	0.095181
C	-0.136440	-0.029483	1.509079
C	1.239421	0.030298	1.906367
C	2.016689	0.122738	0.706982
C	1.137630	0.097763	-0.402577
Fe	0.936674	-1.686263	0.798939
C	-0.029392	-3.427295	1.530673
C	0.423895	-3.710091	0.220522
C	1.834711	-3.543834	0.192660
C	2.259278	-3.151336	1.485650
C	1.099578	-3.068782	2.327418
P	0.967003	-2.286576	3.979072
C	2.096943	-3.323046	5.082300
C	3.515075	-3.419717	4.507133
P	2.020909	-0.306144	3.532074
C	1.334626	1.077097	4.629294
C	1.504916	0.695305	6.100235
C	1.482928	-4.726954	5.154451
C	2.153156	-2.706734	6.480662
C	2.254921	2.265016	4.298848
C	-0.113554	1.486563	4.359296
H	-0.974451	-0.165971	2.177199
H	-1.089046	-0.052068	-0.501438
H	1.426287	0.132922	-1.441784
H	3.095987	0.143550	0.669709
H	-1.050519	-3.415998	1.881742
H	-0.198617	-3.993299	-0.614122
H	2.474580	-3.671449	-0.667214
H	3.267426	-2.885998	1.762990
H	2.158916	-5.368612	5.728172

H	0.510599	-4.712195	5.648032
H	1.361805	-5.163725	4.159518
H	2.683420	-3.397079	7.143341
H	2.701286	-1.762990	6.477447
H	1.153831	-2.539689	6.888478
H	4.153034	-3.900317	5.255124
H	3.539539	-4.039651	3.610808
H	3.941937	-2.439721	4.281986
H	1.930658	3.136092	4.877402
H	3.296795	2.052993	4.550629
H	2.200461	2.529351	3.238683
H	1.245759	1.557652	6.722609
H	0.843054	-0.128816	6.377179
H	2.538894	0.422498	6.328064
H	-0.384722	2.280748	5.063497
H	-0.231528	1.883084	3.349835
H	-0.806752	0.657501	4.503903
Te	-1.278155	-2.271884	4.774731

Calculations at BP86/6-311+G** level of theory

3a-*trans*

E(BP86/6-311+G**)= -2723.755894

C	-0.077666	-0.059622	-0.086166
C	-0.183865	0.066773	1.340501
C	1.143099	0.133116	1.881085
C	2.091262	0.052739	0.786830
C	1.314620	-0.064180	-0.433393
Fe	0.925450	-1.611155	0.843501
C	-0.124492	-3.400901	0.807376
C	0.513372	-3.211863	2.080124
C	1.924320	-3.067774	1.858597
C	2.178363	-3.161068	0.430099
C	0.889041	-3.373663	-0.203932
P	3.669720	-2.887957	-0.628568
C	5.000742	-4.011525	0.157856
C	4.519411	-5.456204	-0.095516
P	3.884986	-0.270626	1.073032
C	4.728965	1.273743	0.336739
C	6.248883	1.010668	0.397150
O	4.222275	-1.326535	-0.235790
C	5.274294	-3.784655	1.653496
C	6.289411	-3.741200	-0.650740
C	4.358637	2.436903	1.280748
C	4.302049	1.583737	-1.106402
H	0.731256	-3.459178	-1.278321
H	-1.194099	-3.516711	0.638658
H	0.010985	-3.154480	3.045063
H	2.666529	-2.860849	2.625815

H	1.724145	-0.192343	-1.433290
H	-0.909010	-0.162521	-0.782542
H	-1.108641	0.076942	1.915649
H	1.403401	0.188100	2.937514
H	7.087292	-4.433380	-0.327272
H	6.129757	-3.887913	-1.732049
H	6.649627	-2.711798	-0.496545
H	5.305778	-6.170267	0.207656
H	3.609859	-5.690329	0.481193
H	4.304418	-5.633758	-1.163357
H	6.155315	-4.376655	1.964194
H	5.489755	-2.726998	1.875145
H	4.428324	-4.107803	2.278217
H	4.895350	3.351336	0.971813
H	3.278571	2.655386	1.254469
H	4.642078	2.222836	2.325739
H	4.881885	2.442321	-1.491923
H	4.487341	0.722760	-1.766763
H	3.234816	1.847866	-1.165726
H	6.799094	1.914432	0.079483
H	6.577714	0.757949	1.419426
H	6.537035	0.185072	-0.272013

3a

E(BP86/6-311+G**)= -2723.745566

C	-0.008546	0.010130	0.020312
C	-0.002185	0.012691	1.455411
C	1.362445	0.043151	1.897620
C	2.208047	0.066424	0.738435
C	1.366746	0.032271	-0.442854
Fe	1.036614	-1.608313	0.687638
C	0.463211	-3.152024	-0.500284
C	1.908316	-3.005129	-0.482614
C	2.321728	-3.181453	0.900478
C	1.159486	-3.415512	1.705025
C	0.011820	-3.398974	0.841984
P	3.101215	-2.257079	-1.673252
C	3.244577	-3.480186	-3.142989
C	3.923315	-4.722913	-2.517489
P	2.105309	-0.215828	-2.123482
O	3.235288	0.760834	-2.414827
C	0.724218	0.051154	-3.420693
C	-0.448307	-0.940978	-3.360728
C	0.229131	1.501293	-3.200072
C	1.414837	-0.023930	-4.799669
C	4.200478	-2.835273	-4.167842
C	1.930373	-3.908290	-3.812593
H	3.296337	0.080574	0.727565
H	1.701022	0.035972	2.932362
H	-0.884204	-0.019300	2.093317

H	-0.901077	-0.026743	-0.598647
H	3.347368	-3.093048	1.255938
H	1.146848	-3.559733	2.784274
H	-1.023813	-3.535230	1.151095
H	-0.178360	-3.054924	-1.373299
H	4.122972	-5.466474	-3.310111
H	4.883921	-4.467700	-2.041872
H	3.279683	-5.201204	-1.761364
H	4.407990	-3.550509	-4.983810
H	3.775457	-1.924594	-4.617588
H	5.160951	-2.559852	-3.703835
H	2.136579	-4.669391	-4.588140
H	1.230679	-4.355159	-3.088869
H	1.430283	-3.063263	-4.307537
H	-1.153076	-0.715275	-4.180941
H	-0.116831	-1.981735	-3.491778
H	-1.011683	-0.877483	-2.418977
H	0.700836	0.296198	-5.578627
H	2.292863	0.637960	-4.836169
H	1.738436	-1.047159	-5.047811
H	-0.482564	1.761558	-4.004055
H	-0.287962	1.631516	-2.237325
H	1.068203	2.212261	-3.239186

2a

E(BP86/6-311+G**)= -2723.760626

C	0.031805	0.021915	0.011008
C	-0.001591	-0.054000	1.456521
C	1.350201	-0.062474	1.936083
C	2.231696	0.016136	0.804853
C	1.428580	0.073357	-0.387438
Fe	0.994959	-1.630740	0.617147
C	1.966666	-3.466171	0.697992
C	0.806087	-3.466391	1.544578
C	-0.339953	-3.143723	0.738877
C	0.107113	-2.937907	-0.629753
C	1.543182	-3.151398	-0.635363
P	-0.751573	-2.201389	-2.098707
C	-2.268354	-3.352253	-2.379925
C	-3.146923	-3.608889	-1.141775
P	-1.486194	-0.326021	-0.983043
O	-2.712925	-0.482046	-0.089843
C	-1.677763	1.139318	-2.171288
C	-2.953146	0.929477	-3.009613
C	-0.453876	1.310120	-3.089798
C	-1.851207	2.379178	-1.263679
C	-3.122427	-2.739140	-3.506896
C	-1.645621	-4.679109	-2.872604
H	-1.357373	-3.009429	1.098785
H	0.796653	-3.657124	2.616949

H	2.990995	-3.656335	1.014808
H	2.185547	-3.040063	-1.507896
H	-0.911867	-0.140424	2.046552
H	1.653799	-0.138276	2.979124
H	3.320236	0.015597	0.838584
H	1.805762	0.102418	-1.407442
H	-2.005990	3.270991	-1.896259
H	-2.723269	2.268845	-0.601209
H	-0.960899	2.557957	-0.639352
H	-3.180068	1.855465	-3.566441
H	-2.826841	0.122927	-3.748150
H	-3.818783	0.693413	-2.371040
H	-0.641254	2.143146	-3.790551
H	0.454030	1.560795	-2.520241
H	-0.254141	0.405249	-3.685724
H	-2.453837	-5.401044	-3.088784
H	-1.058709	-4.540027	-3.795507
H	-0.989603	-5.129147	-2.110020
H	-3.874697	-3.477144	-3.837734
H	-3.667237	-1.847895	-3.161389
H	-2.514852	-2.464804	-4.385851
H	-4.022835	-4.214242	-1.440861
H	-2.602485	-4.180966	-0.375522
H	-3.503777	-2.670305	-0.691267

2a-*cis*

E(BP86/6-311+G**)= -3046.761160

C	-0.053383	0.055810	0.047577
C	-0.002417	0.040728	1.483158
C	1.385389	0.001199	1.890636
C	2.181972	0.011298	0.678054
C	1.295395	0.039953	-0.449413
Fe	0.983409	1.676564	0.764707
C	-0.087326	3.303614	0.114868
C	-0.036066	3.260287	1.549864
C	1.350787	3.311892	1.959231
C	2.146883	3.368666	0.747617
C	1.260613	3.368215	-0.380454
P	2.149776	3.194598	3.618501
S	0.964141	1.598121	4.540941
P	2.182470	0.066336	3.553684
C	1.542487	-1.519826	4.429493
C	0.014195	-1.674218	4.493114
C	2.134651	-1.487980	5.854805
C	2.160323	-2.691558	3.634555
C	1.477197	4.729413	4.558989
C	-0.053955	4.849110	4.628290
C	2.070103	5.945713	3.813551
C	2.070507	4.650912	5.982017
H	3.270524	0.014032	0.643707

H	1.591324	0.073572	-1.496939
H	-0.956955	0.105841	-0.558886
H	-0.857134	0.098999	2.153435
H	3.235240	3.390098	0.713659
H	1.556727	3.384199	-1.428345
H	-0.989917	3.259877	-0.493539
H	-0.889089	3.156461	2.216780
H	-0.241222	-2.599216	5.042539
H	-0.462133	-0.831157	5.018812
H	-0.428211	-1.751803	3.488570
H	1.893546	-2.430230	6.378581
H	3.231841	-1.381914	5.835713
H	1.723290	-0.654933	6.446520
H	1.922801	-3.644976	4.139316
H	1.761517	-2.744194	2.608766
H	3.258564	-2.609693	3.573907
H	-0.328383	5.745016	5.215461
H	-0.498318	4.959006	3.627632
H	-0.512361	3.975234	5.118395
H	1.810010	5.565415	6.544276
H	1.676879	3.785660	6.538530
H	3.169664	4.568655	5.959026
H	1.812954	6.872235	4.357278
H	3.169787	5.889369	3.750039
H	1.669843	6.032471	2.790651

3b-*trans*

E(BP86/6-311+G**)= -3046.756627

C	0.301990	-0.051064	-0.137083
C	0.438241	-0.109709	1.291337
C	1.851368	-0.055461	1.621828
C	2.565125	0.042565	0.363495
C	1.615628	0.049887	-0.708734
Fe	1.260148	1.617796	0.589024
C	0.491803	3.306573	-0.319079
C	-0.027533	3.145439	1.008690
C	1.084697	3.137779	1.938094
C	2.292160	3.293142	1.153453
C	1.922456	3.395452	-0.228846
P	0.848325	2.711535	3.713069
S	2.783630	1.749446	4.137141
P	2.802836	-0.211986	3.200304
C	1.768380	-1.363194	4.337873
C	0.392447	-0.848104	4.787801
C	2.660536	-1.569353	5.583181
C	1.635283	-2.697184	3.570948
C	1.167970	4.386218	4.611452
C	-0.041865	5.269890	4.235752
C	2.476907	5.091019	4.218277
C	1.144202	4.091043	6.125452

H	3.647769	0.114145	0.267812
H	1.847341	0.140386	-1.768961
H	-0.638344	-0.047920	-0.686892
H	-0.383718	-0.151458	2.001218
H	3.304399	3.277211	1.552115
H	2.610406	3.489127	-1.068369
H	-0.094757	3.322680	-1.236707
H	-1.074674	3.011498	1.277622
H	2.181348	-2.293605	6.265703
H	3.654960	-1.960687	5.313375
H	2.800756	-0.625429	6.134481
H	1.197010	-3.461605	4.237091
H	0.983655	-2.602483	2.688349
H	2.615795	-3.073877	3.233157
H	-0.044934	-1.559751	5.512556
H	0.470202	0.131748	5.282687
H	-0.317997	-0.752489	3.954231
H	0.020797	6.228575	4.781389
H	-0.062773	5.497940	3.157996
H	-0.997864	4.791542	4.507451
H	2.566582	6.043382	4.772658
H	3.359662	4.478330	4.459254
H	2.504262	5.324877	3.142639
H	1.186393	5.039197	6.690711
H	0.221599	3.563617	6.420294
H	2.002965	3.473851	6.432726

3b

E(BP86/6-311+G**)= -3046.741004

C	0.006062	0.020210	0.008910
C	-0.000626	0.022403	1.443996
C	1.359847	0.032004	1.899056
C	2.216442	0.046363	0.748521
C	1.386307	0.021843	-0.439489
Fe	1.023125	-1.613855	0.684150
C	0.432899	-3.153347	-0.503277
C	1.878882	-3.017569	-0.485089
C	2.290491	-3.198149	0.899620
C	1.125770	-3.422947	1.702676
C	-0.020893	-3.395316	0.838987
P	3.094984	-2.277649	-1.658534
C	3.237106	-3.481965	-3.145558
C	3.906016	-4.730456	-2.519755
P	2.136567	-0.208590	-2.120395
S	3.636544	1.058826	-2.449593
C	0.730521	0.058185	-3.421556
C	-0.405111	-0.974114	-3.316197
C	0.196499	1.490889	-3.200512
C	1.378167	-0.021273	-4.817713
C	4.205818	-2.834270	-4.156783

C	1.929661	-3.902642	-3.832327
H	3.305006	0.054516	0.744844
H	1.688866	0.018619	2.936767
H	-0.889730	0.003385	2.072720
H	-0.881738	0.001033	-0.616753
H	3.315952	-3.117495	1.257326
H	1.111645	-3.568685	2.781686
H	-1.057921	-3.521490	1.147790
H	-0.206732	-3.053025	-1.376916
H	4.127462	-5.460684	-3.318846
H	4.855017	-4.478720	-2.019451
H	3.246792	-5.222834	-1.786651
H	4.423095	-3.549120	-4.970698
H	3.786624	-1.922751	-4.609968
H	5.160437	-2.558282	-3.681460
H	2.140351	-4.679945	-4.590195
H	1.209907	-4.326127	-3.114123
H	1.454014	-3.059198	-4.353689
H	-1.125298	-0.795360	-4.134218
H	-0.034382	-2.003777	-3.422574
H	-0.960663	-0.906144	-2.371034
H	0.628945	0.269704	-5.574523
H	2.238339	0.660565	-4.895422
H	1.718784	-1.039796	-5.061202
H	-0.567843	1.705762	-3.968694
H	-0.269032	1.620993	-2.212239
H	1.006002	2.229606	-3.299488

2b

E(BP86/6-311+G**)= -3046.756460

C	-0.166437	-0.124496	0.041174
C	-0.123092	-0.093964	1.477187
C	1.269483	-0.014469	1.888785
C	2.064736	0.011374	0.672977
C	1.180992	-0.063493	-0.453262
Fe	0.940066	-1.686761	0.823506
C	-0.101903	-3.297683	1.539015
C	0.313518	-3.570169	0.194485
C	1.743976	-3.461851	0.131272
C	2.226508	-3.117188	1.441872
C	1.076665	-3.007941	2.326805
P	0.978355	-2.261040	4.017658
C	2.101650	-3.353526	5.123802
C	3.563959	-3.340639	4.638622
P	2.065255	-0.272294	3.538545
C	1.351747	1.131483	4.643237
C	1.647553	0.782485	6.113845
C	1.522015	-4.781615	5.035930
C	2.024850	-2.843986	6.573859
C	2.179328	2.370549	4.226188

C	-0.146960	1.424167	4.459305
H	-0.975388	-0.182650	2.147475
H	-1.067456	-0.202153	-0.565776
H	1.480839	-0.087884	-1.499798
H	3.153152	0.035169	0.640604
H	-1.119996	-3.256184	1.921805
H	-0.347827	-3.798035	-0.640040
H	2.360089	-3.597541	-0.756551
H	3.264293	-2.927530	1.705658
H	2.126194	-5.450808	5.673473
H	0.479842	-4.812134	5.388444
H	1.554223	-5.174028	4.006906
H	2.548670	-3.558892	7.232114
H	2.515403	-1.865198	6.688802
H	0.981750	-2.758790	6.915402
H	4.176688	-3.925262	5.347464
H	3.674358	-3.810848	3.650258
H	3.979385	-2.321929	4.593282
H	1.851024	3.241459	4.821959
H	3.257860	2.224074	4.400906
H	2.031744	2.620864	3.162626
H	1.427960	1.659236	6.748760
H	1.017548	-0.049441	6.463872
H	2.707786	0.519093	6.269291
H	-0.446376	2.222153	5.163750
H	-0.363492	1.783074	3.441734
H	-0.763581	0.535591	4.663364
S	-0.898859	-2.209191	4.700191

2b-cis

E(BP86/6-311+G**)= -5050.317138

C	-0.061107	0.057131	0.083360
C	-0.020186	0.035531	1.519273
C	1.364239	-0.006129	1.936396
C	2.169753	0.011876	0.729586
C	1.291288	0.044425	-0.404217
Fe	0.970016	1.675422	0.812996
C	-0.095013	3.300642	0.150558
C	-0.053929	3.263608	1.586152
C	1.329503	3.316875	2.005240
C	2.134695	3.365700	0.799068
C	1.256609	3.361784	-0.335490
P	2.120160	3.241712	3.670848
Se	0.829209	1.593265	4.707487
P	2.153875	0.016496	3.604029
C	1.548143	-1.624387	4.407800
C	0.024154	-1.819286	4.459915
C	2.137575	-1.646207	5.834197
C	2.194442	-2.744651	3.561917
C	1.480619	4.834886	4.541616

C	-0.047083	4.995676	4.600905
C	2.103189	6.002481	3.743277
C	2.069893	4.809809	5.968023
H	3.258536	0.014240	0.703659
H	1.594811	0.083527	-1.449409
H	-0.960345	0.109581	-0.529348
H	-0.878479	0.090418	2.185518
H	3.223278	3.387162	0.773536
H	1.560430	3.372352	-1.381273
H	-0.993224	3.254832	-0.464184
H	-0.910599	3.163256	2.249182
H	-0.209202	-2.781705	4.951988
H	-0.472956	-1.022054	5.036477
H	-0.418110	-1.844809	3.452610
H	1.919550	-2.618391	6.311423
H	3.231758	-1.511923	5.823060
H	1.705079	-0.853453	6.465299
H	1.976951	-3.724977	4.022517
H	1.799851	-2.759715	2.533413
H	3.290661	-2.635559	3.509512
H	-0.300291	5.931797	5.132323
H	-0.490084	5.053725	3.595274
H	-0.527306	4.165046	5.143638
H	1.831799	5.756614	6.485029
H	1.654198	3.982728	6.565464
H	3.166634	4.698927	5.951948
H	1.865475	6.958119	4.244015
H	3.201426	5.918501	3.687002
H	1.708053	6.051970	2.716063

3c-cis

E(BP86/6-311+G**)= -5050.312836

C	-0.081675	0.119696	0.651452
C	0.237853	-0.287855	1.991780
C	1.660342	-0.452023	2.078101
C	2.237365	-0.137424	0.786016
C	1.141633	0.210964	-0.093482
Fe	0.906060	-1.688919	0.634463
C	-0.471112	-3.151401	0.181871
C	0.330731	-3.541535	1.307793
C	1.706833	-3.548895	0.895040
C	1.770294	-3.175369	-0.506748
C	0.406526	-2.929091	-0.928619
P	3.129807	-3.179482	-1.762521
C	4.507234	-4.334000	-1.082356
C	3.842920	-5.715888	-0.887714
P	4.033264	-0.379694	0.467483
C	4.683142	1.426460	0.263387
C	6.171021	1.321741	-0.131339
Se	3.992009	-1.033713	-1.787613

C	5.224885	-3.879895	0.197907
C	5.535019	-4.421364	-2.233540
C	4.565643	2.040997	1.676189
C	3.914483	2.291900	-0.748060
H	0.111806	-2.616689	-1.929640
H	-1.552623	-3.023301	0.178840
H	-0.034906	-3.759444	2.310506
H	2.553632	-3.781963	1.535858
H	1.237892	0.452020	-1.150429
H	-1.082760	0.295968	0.259470
H	-0.476930	-0.474124	2.792286
H	2.218938	-0.783369	2.952523
H	6.336641	-5.129380	-1.958120
H	5.071947	-4.777218	-3.168401
H	6.000821	-3.442204	-2.430800
H	4.622379	-6.467985	-0.670341
H	3.126852	-5.719256	-0.051881
H	3.308763	-6.043707	-1.795622
H	6.016235	-4.608838	0.453952
H	5.700229	-2.897001	0.061431
H	4.549212	-3.805478	1.061611
H	5.007633	3.053635	1.674456
H	3.515074	2.132559	1.995050
H	5.104395	1.440796	2.428484
H	4.350015	3.307780	-0.773463
H	3.967939	1.881431	-1.769225
H	2.853216	2.389513	-0.471948
H	6.629498	2.326947	-0.126293
H	6.734297	0.691149	0.576522
H	6.298831	0.897366	-1.140004

3c

E(BP86/6-311+G**)= -5050.292741

C	0.006632	0.023643	0.012067
C	-0.001324	0.021259	1.447170
C	1.358749	0.024384	1.903705
C	2.216791	0.039930	0.754324
C	1.387672	0.025794	-0.433628
Fe	1.018582	-1.615720	0.682620
C	0.424377	-3.155568	-0.503680
C	1.869905	-3.020028	-0.492756
C	2.288972	-3.197959	0.890114
C	1.128745	-3.425211	1.698833
C	-0.022457	-3.397928	0.841203
P	3.085913	-2.278771	-1.668878
C	3.229788	-3.489147	-3.152783
C	3.901979	-4.727654	-2.508471
P	2.131491	-0.201140	-2.116399
Se	3.756164	1.165807	-2.454044
C	0.726855	0.071992	-3.419522

C	-0.398086	-0.972725	-3.317561
C	0.176938	1.498218	-3.198427
C	1.381346	-0.002845	-4.811750
C	4.200373	-2.849802	-4.166545
C	1.928200	-3.928225	-3.840201
H	3.305310	0.043298	0.748497
H	1.686661	0.005919	2.941757
H	-0.891046	0.004178	2.074897
H	-0.880752	0.011243	-0.614263
H	3.316169	-3.114733	1.242258
H	1.120223	-3.571218	2.777934
H	-1.057745	-3.525760	1.154866
H	-0.218221	-3.059811	-1.375677
H	4.123764	-5.468614	-3.297466
H	4.851075	-4.466902	-2.013163
H	3.244869	-5.210665	-1.767294
H	4.435115	-3.577772	-4.963800
H	3.774691	-1.952992	-4.642388
H	5.146073	-2.551139	-3.687238
H	2.150350	-4.717328	-4.582557
H	1.206657	-4.346968	-3.121037
H	1.447714	-3.100663	-4.382596
H	-1.122951	-0.794551	-4.131535
H	-0.016088	-1.997289	-3.433840
H	-0.949757	-0.916965	-2.369472
H	0.626738	0.256995	-5.574403
H	2.222845	0.702005	-4.892221
H	1.753748	-1.013930	-5.038685
H	-0.578084	1.708749	-3.977053
H	-0.306202	1.620080	-2.217450
H	0.980215	2.245862	-3.281338

2c

E(BP86/6-311+G**)= -5050.308517

C	-0.206713	-0.145065	0.054339
C	-0.149343	-0.116324	1.489317
C	1.246025	-0.016805	1.886578
C	2.028906	0.027128	0.662690
C	1.134149	-0.058872	-0.453989
Fe	0.936833	-1.690290	0.821809
C	-0.075269	-3.327290	1.527690
C	0.355856	-3.588181	0.186361
C	1.783814	-3.447873	0.133431
C	2.249369	-3.093782	1.447223
C	1.090135	-3.009083	2.323658
P	0.971412	-2.266891	4.015458
C	2.094611	-3.357750	5.133590
C	3.559661	-3.326448	4.657490
P	2.056773	-0.273384	3.527754
C	1.367901	1.142246	4.632739

C	1.635921	0.777844	6.104209
C	1.529650	-4.790624	5.034270
C	1.998858	-2.858640	6.585951
C	2.236089	2.357944	4.228323
C	-0.118869	1.477244	4.430432
H	-0.990924	-0.223853	2.171004
H	-1.112050	-0.238964	-0.543739
H	1.423740	-0.074292	-1.503617
H	3.116282	0.071211	0.617500
H	-1.095231	-3.311458	1.908084
H	-0.294484	-3.831204	-0.652583
H	2.409192	-3.570120	-0.749775
H	3.280551	-2.880562	1.718953
H	2.130920	-5.455176	5.679359
H	0.482915	-4.829761	5.372351
H	1.578190	-5.180988	4.005231
H	2.529874	-3.569859	7.242307
H	2.469730	-1.872123	6.714897
H	0.951106	-2.796301	6.918886
H	4.174527	-3.903928	5.370201
H	3.681530	-3.795073	3.669875
H	3.962982	-2.302781	4.614252
H	1.925406	3.237555	4.820627
H	3.307571	2.180173	4.416571
H	2.109689	2.613899	3.163365
H	1.424630	1.653337	6.743648
H	0.983836	-0.044295	6.437250
H	2.688583	0.492597	6.271835
H	-0.403231	2.285937	5.128840
H	-0.315061	1.837313	3.409111
H	-0.764389	0.608261	4.631955
Se	-1.063555	-2.233492	4.735060

2c-*cis*

E(BP86/6-311+G**)= -2916.679294

C	-0.077678	0.058160	0.127107
C	-0.051801	0.022524	1.562910
C	1.327536	-0.018051	1.994999
C	2.146377	0.015183	0.797448
C	1.280221	0.054383	-0.345842
Fe	0.941480	1.673778	0.878486
C	-0.111494	3.297513	0.194228
C	-0.085749	3.274181	1.630286
C	1.292627	3.325595	2.064284
C	2.111468	3.359090	0.866741
C	1.245826	3.349212	-0.277568
P	2.066448	3.303499	3.739782
Te	0.630437	1.586441	4.936558
P	2.101459	-0.049221	3.670306
C	1.558314	-1.759167	4.378244

C	0.044389	-2.023760	4.399716
C	2.132548	-1.834586	5.808677
C	2.260885	-2.806214	3.483769
C	1.488000	4.970939	4.517680
C	-0.031113	5.202746	4.549370
C	2.168209	6.068575	3.667676
C	2.061089	4.999008	5.950283
H	3.235393	0.020772	0.784343
H	1.595614	0.104424	-1.387144
H	-0.970280	0.112702	-0.495096
H	-0.916116	0.062999	2.222932
H	3.200358	3.376775	0.853889
H	1.561746	3.348938	-1.319912
H	-1.003032	3.250175	-0.430087
H	-0.948746	3.188375	2.287690
H	-0.151548	-3.028052	4.819526
H	-0.490630	-1.291975	5.027671
H	-0.390454	-1.994525	3.389173
H	1.955565	-2.841242	6.227844
H	3.219054	-1.648608	5.820586
H	1.656458	-1.100276	6.478732
H	2.081646	-3.817427	3.891626
H	1.876615	-2.785271	2.451579
H	3.351514	-2.647349	3.449681
H	-0.247816	6.184479	5.010320
H	-0.465642	5.206315	3.538276
H	-0.550490	4.434568	5.146223
H	1.863289	5.983521	6.410704
H	1.600695	4.227792	6.589118
H	3.151246	4.835421	5.954997
H	1.968051	7.058065	4.116977
H	3.261905	5.934066	3.627561
H	1.784064	6.082381	2.635319

3c-*trans*

E(BP86/6-311+G**)= -2916.675134

C	-0.358516	0.452330	-0.441899
C	-0.711567	0.336310	0.942303
C	0.431127	-0.171479	1.648768
C	1.494178	-0.357913	0.700268
C	1.009774	0.012202	-0.617019
Fe	0.895935	1.597291	0.721189
C	0.964148	3.487037	-0.069645
C	0.208405	3.489450	1.150722
C	1.059443	3.014938	2.206643
C	2.342683	2.715413	1.640076
C	2.301197	3.015526	0.221997
P	3.756769	2.673421	-0.848593
Te	2.621376	1.978354	-3.022281
P	1.686379	-0.246689	-2.321072

C	3.168407	-1.466119	-2.168347
C	3.608293	-1.721978	-3.627568
C	2.583795	-2.773272	-1.584609
C	4.380607	-0.987935	-1.354678
C	4.377043	4.445916	-1.310615
C	3.307427	5.383722	-1.892243
C	5.539931	4.265454	-2.307722
C	4.918988	5.030327	0.013493
H	-1.004328	0.811679	-1.242093
H	-1.669237	0.608837	1.383596
H	0.495144	-0.350798	2.721283
H	2.493305	-0.712746	0.939986
H	0.591372	3.751772	-1.057462
H	-0.838799	3.771788	1.253317
H	0.770588	2.873521	3.247328
H	3.203934	2.312169	2.171198
H	4.407123	-2.484538	-3.640985
H	2.774203	-2.090965	-4.246602
H	4.007153	-0.806794	-4.094903
H	3.352876	-3.565460	-1.624678
H	2.269292	-2.660851	-0.536164
H	1.714214	-3.122943	-2.165922
H	5.174450	-1.757353	-1.389743
H	4.793707	-0.054684	-1.766923
H	4.141179	-0.805340	-0.297356
H	5.368974	6.021181	-0.178523
H	4.117990	5.162455	0.758321
H	5.698446	4.387078	0.454537
H	3.754354	6.370845	-2.112304
H	2.883753	4.993343	-2.832334
H	2.479183	5.541344	-1.184576
H	6.026847	5.239632	-2.493314
H	6.305239	3.574472	-1.917122
H	5.192943	3.872613	-3.277378

3c

E(BP86/6-311+G**)= -2916.652277

C	-0.008376	-0.002328	0.000583
C	-0.023993	0.002761	1.435584
C	1.333111	0.023404	1.899064
C	2.196803	0.042150	0.754415
C	1.375365	0.009067	-0.438791
Fe	1.016475	-1.627942	0.684899
C	0.446890	-3.178346	-0.499805
C	1.890990	-3.030031	-0.475583
C	2.298799	-3.197458	0.911912
C	1.132680	-3.432241	1.710513
C	-0.010499	-3.418998	0.841846
P	3.108031	-2.284826	-1.647687
C	3.234592	-3.486968	-3.139195

C	3.910711	-4.733111	-2.515665
P	2.133962	-0.217990	-2.118999
Te	3.940606	1.296761	-2.464377
C	0.726324	0.063803	-3.431643
C	-0.419305	-0.958825	-3.322913
C	0.201196	1.499942	-3.212119
C	1.367833	-0.025701	-4.829162
C	4.188464	-2.839786	-4.163401
C	1.917729	-3.909873	-3.804815
H	3.285291	0.064968	0.755587
H	1.655770	0.015125	2.938859
H	-0.917161	-0.021236	2.058228
H	-0.892869	-0.025813	-0.629048
H	3.321909	-3.103950	1.273124
H	1.114757	-3.571942	2.790229
H	-1.047578	-3.553755	1.146546
H	-0.189233	-3.088892	-1.377365
H	4.116176	-5.468980	-3.313742
H	4.869098	-4.481351	-2.033802
H	3.262884	-5.219098	-1.768058
H	4.382663	-3.548679	-4.988388
H	3.768718	-1.919180	-4.597681
H	5.154641	-2.575299	-3.705522
H	2.117663	-4.690695	-4.561841
H	1.208155	-4.329928	-3.074782
H	1.434975	-3.069243	-4.323973
H	-1.159618	-0.740973	-4.113069
H	-0.069123	-1.989353	-3.477473
H	-0.944903	-0.916125	-2.360086
H	0.615943	0.266226	-5.583099
H	2.230343	0.653813	-4.916056
H	1.703493	-1.046505	-5.071222
H	-0.562275	1.715000	-3.981335
H	-0.263513	1.636579	-2.224332
H	1.014381	2.235153	-3.314235

3c-*trans*

E(BP86/6-311+G**)=-2916.667729

C	-0.196206	-0.137382	0.050760
C	-0.148825	-0.112149	1.486044
C	1.243693	-0.021085	1.892724
C	2.036249	0.020258	0.675128
C	1.148709	-0.057435	-0.447999
Fe	0.933981	-1.691232	0.822340
C	-0.087858	-3.328726	1.512588
C	0.354824	-3.586263	0.174626
C	1.783247	-3.447683	0.135087
C	2.237171	-3.097942	1.453998
C	1.070231	-3.016216	2.321603
P	0.950695	-2.282145	4.019380

C	2.100893	-3.364646	5.129485
C	3.559827	-3.327941	4.634285
P	2.040287	-0.286453	3.538447
C	1.366190	1.144816	4.633609
C	1.610142	0.785539	6.110846
C	1.552470	-4.804249	5.049888
C	2.023314	-2.850987	6.577460
C	2.264254	2.339460	4.231444
C	-0.110312	1.510014	4.413909
H	-0.995309	-0.214910	2.163407
H	-1.097970	-0.223098	-0.553962
H	1.445704	-0.071075	-1.495586
H	3.124131	0.056478	0.638161
H	-1.112474	-3.311012	1.881783
H	-0.288363	-3.824872	-0.670976
H	2.416920	-3.568786	-0.742455
H	3.266025	-2.886702	1.735152
H	2.176286	-5.455827	5.687030
H	0.513365	-4.858024	5.409467
H	1.588396	-5.200331	4.022340
H	2.588747	-3.538809	7.230147
H	2.468863	-1.849921	6.681297
H	0.982123	-2.811819	6.935124
H	4.183941	-3.901518	5.342082
H	3.668454	-3.800822	3.647447
H	3.960639	-2.303919	4.583277
H	1.968895	3.226846	4.819958
H	3.330141	2.138181	4.427583
H	2.150815	2.595535	3.165044
H	1.412357	1.671835	6.739693
H	0.934562	-0.017290	6.445229
H	2.653590	0.476881	6.293708
H	-0.385454	2.326457	5.106878
H	-0.290273	1.869667	3.389507
H	-0.776803	0.655967	4.614266
Te	-1.302184	-2.285511	4.829522

4a

E(ωB97XD/6-311+G**)= -2798.501221

C	0.035217	0.039048	0.002795
C	0.031693	-0.018672	1.432901
C	1.379857	-0.046254	1.874708
C	2.224445	0.001015	0.730710
C	1.404358	0.054720	-0.427848
Fe	0.970637	-1.631778	0.606686
C	1.933155	-3.463875	0.677848
C	0.803830	-3.461761	1.543267
C	-0.349606	-3.159312	0.771360
C	0.069603	-2.970531	-0.588358

C	1.486597	-3.166503	-0.635816
P	-0.828411	-2.210836	-1.974312
C	-2.262311	-3.321704	-2.356481
C	-3.108009	-3.630433	-1.114443
P	-1.470489	-0.285956	-0.962222
O	-2.672173	-0.496330	-0.087394
C	-1.651967	1.116858	-2.160003
C	-2.835559	0.834103	-3.090530
C	-0.378878	1.347799	-2.984018
C	-1.955686	2.347204	-1.287935
C	-3.132013	-2.667359	-3.434482
C	-1.629970	-4.611329	-2.907304
H	-1.351013	-3.020038	1.148971
H	0.819677	-3.632664	2.609117
H	2.956465	-3.642023	0.971868
H	2.092688	-3.045483	-1.520759
H	-0.857401	-0.086994	2.041152
H	1.706867	-0.111299	2.901439
H	3.303643	-0.027306	0.736014
H	1.744843	0.033193	-1.451782
H	-2.095912	3.214890	-1.940565
H	-2.864455	2.205339	-0.698975
H	-1.130949	2.570893	-0.605087
H	-3.063230	1.739297	-3.662268
H	-2.597778	0.044668	-3.806048
H	-3.731339	0.554791	-2.529806
H	-0.583703	2.123910	-3.728938
H	0.441833	1.705706	-2.359140
H	-0.059863	0.441631	-3.504716
H	-2.430136	-5.308775	-3.175203
H	-1.029379	-4.413473	-3.797821
H	-0.995224	-5.099386	-2.162039
H	-3.859781	-3.399717	-3.797969
H	-3.691148	-1.820170	-3.032807
H	-2.534459	-2.333634	-4.286769
H	-3.982700	-4.211039	-1.425930
H	-2.554437	-4.237535	-0.395334
H	-3.453387	-2.719727	-0.619165
O	0.063673	-1.944748	-3.152132

TS between **4a** and **5a**

E(ωB97XD/6-311+G**)= -2798.434434

P	-0.083277	-0.098517	0.018560
P	0.181455	-0.112339	2.335633
O	1.481321	-0.250571	1.424780
C	-0.121998	-1.898802	-0.146609
C	0.932923	-2.859697	-0.023533
C	0.352198	-4.152152	-0.103058
C	-1.055164	-4.001557	-0.263191

C	-1.350948	-2.615471	-0.296051
Fe	-0.417139	-3.074563	1.472211
C	-0.234622	-1.753514	2.984332
C	-1.557405	-2.307393	2.973890
C	-1.446669	-3.712433	3.146356
C	-0.066578	-4.037684	3.262602
C	0.683782	-2.835013	3.167418
C	1.160926	0.700384	-1.120100
C	0.463383	0.812073	-2.486598
O	-1.422069	0.492573	-0.283115
C	0.576556	0.968375	3.787167
C	-0.621904	0.978724	4.740452
C	1.472796	2.100396	-0.576257
C	2.449879	-0.110141	-1.268667
C	1.823804	0.394075	4.469365
C	0.859026	2.378358	3.256407
H	1.759737	-2.747230	3.161278
H	0.344007	-5.030019	3.375171
H	-2.265799	-4.415586	3.156901
H	-2.469204	-1.753228	2.806938
H	1.969477	-2.624635	0.155840
H	0.882963	-5.088679	-0.018772
H	-1.775760	-4.803180	-0.325095
H	-2.322473	-2.153064	-0.381898
H	-0.406666	1.635667	5.589344
H	-1.524181	1.353819	4.247968
H	-0.832908	-0.019427	5.133632
H	1.075175	3.050532	4.093109
H	1.723601	2.380881	2.588934
H	0.001835	2.780824	2.708919
H	2.147187	1.069754	5.268622
H	1.622167	-0.583453	4.915473
H	2.647061	0.289245	3.757232
H	3.130240	0.439810	-1.927621
H	2.940833	-0.260000	-0.307929
H	2.260433	-1.081793	-1.731936
H	1.157266	1.275679	-3.195910

5a

E(ωB97XD/6-311+G**)= -2798.517301

C	0.001864	-0.014546	-0.067939
C	-0.008304	-0.030087	1.354847
C	1.337713	-0.033181	1.808071
C	2.189695	-0.028289	0.657425
C	1.351243	-0.019789	-0.500355
Fe	1.001224	1.625018	0.685727
C	0.074834	3.353078	0.046845
C	0.047022	3.266840	1.466190
C	1.385429	3.184160	1.933132
C	2.263041	3.227758	0.799578

C	1.430716	3.332994	-0.363899
P	4.069702	3.046817	0.583905
O	4.331157	1.510811	1.279079
P	3.982432	0.121867	0.516587
C	4.822396	-1.102050	1.598295
C	4.420241	-0.965233	3.070783
O	4.397326	0.053324	-0.907413
C	4.841467	4.041658	1.966924
C	4.439093	3.624780	3.383309
C	4.462919	5.506690	1.711459
C	6.358995	3.857059	1.798016
C	6.332223	-0.859136	1.442982
C	4.453532	-2.496772	1.071810
H	1.716845	0.010534	-1.515433
H	-0.867574	0.022762	-0.706825
H	-0.885977	-0.007504	1.983401
H	1.654959	0.004720	2.838172
H	1.793501	3.354774	-1.381234
H	-0.785876	3.399527	-0.603171
H	-0.839957	3.231324	2.081364
H	1.678751	3.053573	2.961723
H	5.041095	-1.636388	3.673081
H	4.569714	0.053270	3.436047
H	3.379554	-1.252366	3.233522
H	6.878407	-1.624764	2.003290
H	6.636300	-0.913410	0.395639
H	6.617475	0.120142	1.835310
H	4.972481	-3.256494	1.665045
H	3.378509	-2.685345	1.150168
H	4.748239	-2.615099	0.026807
H	5.097584	4.116792	4.107790
H	3.418285	3.927114	3.620145
H	4.530858	2.545316	3.521881
H	6.890217	4.490967	2.515878

4b

E(ωB97XD/6-311+G**)=-3444.455310

C	0.076891	-0.002663	-0.034277
C	0.081880	0.007307	1.396945
C	1.430579	-0.026540	1.830968
C	2.269313	-0.047851	0.681885
C	1.445459	-0.031456	-0.473677
Fe	0.981316	-1.657799	0.635144
C	1.897063	-3.509045	0.802074
C	0.758720	-3.437724	1.652645
C	-0.377156	-3.139586	0.855785
C	0.064155	-3.021213	-0.507341
C	1.475266	-3.259792	-0.527740
P	-0.837194	-2.329922	-1.929924
C	-2.311386	-3.453176	-2.244053

C	-2.985052	-3.916308	-0.946015
P	-1.429393	-0.308211	-1.010130
S	-3.041666	-0.459471	0.118625
C	-1.548356	1.070228	-2.282624
C	-2.532925	0.662018	-3.381092
C	-0.187438	1.423550	-2.896003
C	-2.082510	2.294537	-1.521701
C	-3.322184	-2.725838	-3.133869
C	-1.749456	-4.682065	-2.977176
H	-1.379272	-2.960808	1.212851
H	0.757958	-3.560461	2.725198
H	2.911948	-3.701539	1.115272
H	2.094966	-3.199097	-1.409344
H	-0.803726	-0.011956	2.013614
H	1.761122	-0.050556	2.858251
H	3.347760	-0.096678	0.682697
H	1.778804	-0.101611	-1.497219
H	-2.098321	3.142206	-2.214205
H	-3.092004	2.130579	-1.144106
H	-1.438170	2.561870	-0.678848
H	-2.699061	1.519699	-4.040273
H	-2.134976	-0.154133	-3.987748
H	-3.499552	0.367911	-2.965709
H	-0.351706	2.196448	-3.653763
H	0.493677	1.835725	-2.149313
H	0.281551	0.567003	-3.380044
H	-2.567515	-5.396158	-3.115736
H	-1.341131	-4.421940	-3.954002
H	-0.965775	-5.178181	-2.396866
H	-4.107381	-3.431425	-3.422616
H	-3.795476	-1.897604	-2.602384
H	-2.856530	-2.351786	-4.048556
H	-3.854607	-4.523427	-1.217720
H	-2.319310	-4.544349	-0.351281
H	-3.330773	-3.079339	-0.339481

G(ωB97XD/6-311G**)= -3444.079363
E(ωB97XD/6-311G**)=-3444.437806

C	0.013262	0.006417	0.016221
C	0.029631	-0.002124	1.432817
C	1.384723	-0.019073	1.863657
C	2.218381	-0.022358	0.716492
C	1.368840	-0.013815	-0.442318
Fe	1.017109	-1.641451	0.657933
C	0.397777	-3.137734	-0.544399
C	1.829988	-3.022619	-0.531625
C	2.268407	-3.236685	0.813820
C	1.123836	-3.467078	1.616292
C	-0.026052	-3.407084	0.781808
P	2.911138	-2.356789	-1.836134
C	2.778792	-3.521172	-3.302168

C	3.645956	-4.737040	-2.937134
P	1.829428	-0.353312	-2.170484
S	0.254577	-0.548747	-3.341895
C	2.981321	1.026772	-2.710094
C	2.070168	2.233373	-2.988443
C	3.992264	1.409983	-1.622077
C	3.710442	0.608666	-3.988992
S	4.766054	-2.102135	-1.215622
C	3.334055	-2.831308	-4.550288
C	1.341210	-3.994695	-3.550692
H	3.294843	-0.091478	0.711065
H	1.726319	-0.052492	2.886945
H	-0.839744	-0.015040	2.072161
H	-0.852759	-0.020923	-0.627060
H	3.294559	-3.175119	1.142107
H	1.126341	-3.637471	2.681985
H	-1.050728	-3.518488	1.102044
H	-0.242134	-2.976504	-1.397686
H	3.525590	-5.488406	-3.723733
H	4.701226	-4.475263	-2.858432
H	3.331574	-5.185622	-1.990067
H	3.387520	-3.563559	-5.361619
H	2.684229	-2.017000	-4.877645
H	4.342153	-2.445817	-4.380857
H	1.348119	-4.631766	-4.440770
H	0.968511	-4.595795	-2.719450
H	0.658121	-3.165020	-3.731550
H	2.706584	3.093877	-3.217105
H	1.403745	2.053897	-3.832234
H	1.459951	2.486372	-2.116306
H	4.265389	1.469509	-4.374083

TS between **4b** and **5b**

E(ωB97XD/6-311+G**)=-3444.388089

C	-0.117242	-0.143284	-0.066584
C	-0.307513	-0.252815	1.344981
C	0.954629	-0.091416	1.973852
C	1.930129	0.128965	0.961734
C	1.276528	0.095150	-0.299096
Fe	0.994720	-1.673180	0.651165
C	1.724635	-3.069445	-0.643900
C	2.359284	-3.222649	0.616653
C	1.357891	-3.473066	1.595770
C	0.094888	-3.469914	0.948533
C	0.316598	-3.221007	-0.448783
P	-0.993058	-2.637253	-1.540757
S	-0.225386	-1.524169	-3.106339
P	-1.471803	-0.336066	-1.284868
S	-3.291663	-0.455223	-0.497563
C	-1.459781	1.315557	-2.258825

C	-0.090299	1.705224	-2.815877
C	-1.903014	2.411848	-1.272143
C	-2.467152	1.191379	-3.404799
C	-2.003773	-4.074485	-2.148406
C	-3.139713	-3.520621	-3.016694
C	-1.096502	-5.004801	-2.956975
C	-2.577663	-4.792262	-0.920103
H	1.524065	-3.615498	2.652914
H	-1.790410	-5.230609	-0.301438
H	1.749425	0.164218	-1.265321
H	2.990048	0.262392	1.117862
H	1.142118	-0.146745	3.035601
H	-1.255679	-0.452874	1.820863
H	2.197822	-2.815541	-1.580331
H	3.419363	-3.137294	0.802199
H	-0.867285	-3.583122	1.425121
H	-1.692168	-5.834491	-3.352980
H	-0.645363	-4.483536	-3.806437
H	-0.296994	-5.425133	-2.340400
H	-3.758338	-4.353695	-3.365946
H	-3.773846	-2.831764	-2.452993
H	-2.750968	-2.994166	-3.891189
H	-3.227612	-5.606333	-1.256169
H	-3.177160	-4.113567	-0.306622
H	-1.828354	3.379959	-1.779378
H	-2.930543	2.263788	-0.943902
H	-1.258146	2.447539	-0.388850
H	-2.549896	2.157894	-3.912230
H	-2.141581	0.450080	-4.139041
H	-3.456056	0.912685	-3.036612
H	-0.216268	2.601795	-3.431298
H	0.615541	1.953962	-2.022001

5b

E(ωB97XD/6-311+G**)=-3444.456385

C	0.007675	-0.051686	0.089237
C	0.067015	0.016323	1.504856
C	1.436511	0.084482	1.884185
C	2.224602	0.067206	0.703205
C	1.345928	-0.026440	-0.422085
Fe	0.968733	1.665886	0.666813
C	-0.053850	3.313444	-0.018057
C	0.051854	3.362773	1.394224
C	1.431731	3.352082	1.739405
C	2.187606	3.282532	0.540016
C	1.268117	3.266343	-0.557742
P	1.634799	3.162167	-2.326567
S	0.015194	2.945803	-3.400983
P	1.651090	-0.056044	-2.223204
S	3.064860	1.574279	-2.450662

C	2.823642	-1.518470	-2.457637
C	3.185434	-1.552459	-3.950154
C	1.973150	-2.751356	-2.109907
C	4.099842	-1.526372	-1.613538
C	2.645963	4.672908	-2.769482
C	1.758604	5.891548	-2.475024
C	2.974119	4.607533	-4.266473
C	3.940238	4.770096	-1.952969
H	-0.886283	-0.078984	-0.516367
H	-0.778320	0.044051	2.175903
H	1.810631	0.178195	2.892955
H	3.297960	0.163546	0.656206
H	-0.957377	3.286173	-0.607514
H	-0.774383	3.373945	2.088993
H	1.836141	3.349891	2.740596
H	3.260977	3.203284	0.469316
H	4.714087	-2.388546	-1.897198
H	4.700824	-0.626419	-1.766966
H	3.876824	-1.619406	-0.549519
H	3.736973	-2.472534	-4.171288
H	2.291754	-1.533254	-4.580128
H	3.815947	-0.705586	-4.232769
H	2.552552	-3.659310	-2.307945
H	1.688455	-2.758492	-1.053882
H	1.061462	-2.797128	-2.712940
H	4.511192	5.631464	-2.313683
H	3.739502	4.932038	-0.892682
H	4.570466	3.884495	-2.061961
H	3.497066	5.525516	-4.552477
H	3.622990	3.759951	-4.500979
H	2.067578	4.524011	-4.868545
H	2.311604	6.799691	-2.735007
H	0.839529	5.866900	-3.063525

G(ωB97XD/6-311G**)= -3444.084131
E(ωB97XD/6-311G**)=-3444.437880

C	0.001926	-0.004087	-0.009707
C	0.006415	0.004465	1.418260
C	1.354707	-0.003315	1.851649
C	2.192617	-0.010245	0.703185
C	1.364551	-0.019354	-0.448756
Fe	0.970912	-1.649186	0.685239
C	0.057359	-3.286846	-0.124533
C	0.005048	-3.341673	1.305605
C	1.332133	-3.331198	1.804283
C	2.217674	-3.270041	0.693804
C	1.439016	-3.233414	-0.492044
P	-1.485734	-3.254570	-1.102280
C	-1.279374	-4.677604	-2.323261
C	-0.016289	-4.674151	-3.186782
P	-1.503403	-0.033519	-1.013054

C	-1.493300	1.513396	-2.061129
C	-0.262713	1.590855	-2.972687
S	-3.106014	-0.245610	0.085048
S	-1.115509	-1.583114	-2.435288
C	-2.525656	-4.662124	-3.221364
C	-1.296172	-5.940418	-1.446379
C	-1.486348	2.700202	-1.085898
C	-2.774003	1.528382	-2.904949
H	-0.901819	-3.351923	1.892058
H	1.619841	-3.335301	2.844759
H	3.294966	-3.215535	0.743934
H	1.820138	-3.130784	-1.495811
H	-0.879300	-0.009947	2.034282
H	1.686963	-0.032257	2.878266
H	3.271596	-0.047327	0.704053
H	1.701778	-0.077636	-1.471687
H	-0.060130	-5.512375	-3.891047
H	0.081572	-3.755423	-3.770542
H	0.883059	-4.800936	-2.582332
H	-2.532749	-5.557888	-3.851240
H	-3.445410	-4.655397	-2.630089
H	-2.540530	-3.788316	-3.877640
H	-1.275205	-6.827668	-2.087645
H	-0.425595	-5.981902	-0.785721
H	-2.198916	-5.993055	-0.830745
H	-0.348080	2.490849	-3.589341
H	0.662073	1.673336	-2.399564
H	-0.186233	0.733605	-3.645215
H	-2.801560	2.454880	-3.486676

4c

E(ωB97XD/6-311+G**)= -7451.215068

C	0.073372	0.000987	-0.033891
C	0.063661	0.009352	1.397779
C	1.407400	-0.012377	1.845997
C	2.258479	-0.023316	0.705970
C	1.447513	-0.013881	-0.458476
Fe	0.987735	-1.646579	0.642602
C	1.921912	-3.488453	0.822367
C	0.770412	-3.430325	1.655836
C	-0.356919	-3.144221	0.842946
C	0.103009	-3.020884	-0.514145
C	1.517193	-3.244178	-0.513078
P	-0.791319	-2.342231	-1.948506
C	-2.255891	-3.484921	-2.283136
C	-2.909054	-3.985990	-0.988642
P	-1.417015	-0.314209	-1.032477
Se	-3.170180	-0.476296	0.167794
C	-1.533683	1.069654	-2.310802
C	-2.469341	0.637926	-3.442222
C	-0.159999	1.460737	-2.870576

C	-2.123173	2.282298	-1.573051
C	-3.281684	-2.754095	-3.152368
C	-1.687655	-4.694185	-3.042683
H	-1.366277	-2.978409	1.185907
H	0.753743	-3.554451	2.728193
H	2.934810	-3.668322	1.149339
H	2.148951	-3.177438	-1.385610
H	-0.828029	-0.018865	2.005202
H	1.725667	-0.036044	2.877194
H	3.337426	-0.060370	0.717183
H	1.793202	-0.077851	-1.478452
H	-2.130407	3.126740	-2.269683
H	-3.142094	2.097409	-1.232990
H	-1.517343	2.566354	-0.707665
H	-2.629239	1.492427	-4.106957
H	-2.034685	-0.170268	-4.034061
H	-3.443827	0.325896	-3.059521
H	-0.315883	2.231612	-3.632150
H	0.480418	1.886559	-2.095944
H	0.350600	0.620164	-3.339619
H	-2.500879	-5.413444	-3.183176
H	-1.292556	-4.413963	-4.019131
H	-0.892876	-5.191956	-2.479493
H	-4.059997	-3.465612	-3.444971
H	-3.761006	-1.939336	-2.605813
H	-2.827036	-2.362703	-4.065371
H	-3.770095	-4.603039	-1.265062
H	-2.225090	-4.612210	-0.412826
H	-3.265650	-3.169286	-0.361702

E(ωB97XD/6-311G**)= -7450.844562
E(ωB97XD/6-311+G**)=-7451.199290

C	0.010201	0.005085	0.018160
C	0.028811	-0.004541	1.434644
C	1.384255	-0.018529	1.863594
C	2.216304	-0.022085	0.715629
C	1.365380	-0.014145	-0.442609
Fe	1.016071	-1.642796	0.659997
C	0.401967	-3.141983	-0.542392
C	1.834627	-3.029100	-0.522550
C	2.265079	-3.240169	0.826282
C	1.115520	-3.465296	1.622955
C	-0.029296	-3.407705	0.781823
P	2.916994	-2.369836	-1.830626
C	2.782251	-3.538636	-3.304401
C	3.683521	-4.739028	-2.975231
P	1.834130	-0.351519	-2.169940
Se	0.137633	-0.545713	-3.440706
C	2.997771	1.030822	-2.709750
C	2.099777	2.231897	-3.045300
C	3.967223	1.443227	-1.594907

C	3.769688	0.586873	-3.954107
Se	4.921361	-2.111863	-1.162310
C	3.288776	-2.829332	-4.562051
C	1.350351	-4.046988	-3.514227
H	3.292975	-0.090610	0.709006
H	1.728386	-0.049369	2.886207
H	-0.840528	-0.018330	2.074147
H	-0.856321	-0.026108	-0.624477
H	3.289450	-3.176124	1.160017
H	1.113172	-3.632348	2.689254
H	-1.056200	-3.518614	1.095325
H	-0.233288	-2.981911	-1.399592
H	3.567424	-5.475046	-3.777015
H	4.733413	-4.454432	-2.906841
H	3.394144	-5.217314	-2.035053
H	3.328908	-3.555967	-5.379134
H	2.617707	-2.022546	-4.863935
H	4.296083	-2.431359	-4.419236
H	1.348439	-4.682547	-4.405437
H	1.015891	-4.656266	-2.672629
H	0.642255	-3.234821	-3.676091
H	2.749540	3.078676	-3.287860
H	1.450119	2.030381	-3.896936
H	1.474461	2.520332	-2.195467
H	4.340406	1.440298	-4.332501

TS between **4c** and **5c**

E(ωB97XD/6-311+G**)=-7451.152863

C	-0.001793	-0.003383	-0.001873
C	-0.008127	-0.004801	1.426620
C	1.339085	0.000074	1.874205
C	2.186301	0.014400	0.731458
C	1.366458	0.011191	-0.428471
Fe	0.969736	-1.640536	0.678048
C	1.310130	-3.201722	-0.588253
C	2.075142	-3.384822	0.593210
C	1.185837	-3.419670	1.703017
C	-0.137014	-3.252553	1.216707
C	-0.067359	-3.120320	-0.212462
P	-1.414446	-2.417110	-1.183339
Se	-0.690867	-1.502644	-3.059275
P	-1.521093	-0.048777	-1.031357
Se	-3.361823	0.094108	0.074470
C	-1.407460	1.605501	-2.020958
C	-0.093469	1.785316	-2.780228
C	-1.518136	2.741437	-0.986815
C	-2.580687	1.657813	-3.000816
C	-2.718074	-3.717346	-1.478612

C	-3.868567	-3.086222	-2.270375
C	-2.081383	-4.867950	-2.262130
C	-3.217956	-4.191042	-0.107390
H	1.467460	-3.527829	2.739525
H	-2.431397	-4.693573	0.461003
H	1.707079	-0.041758	-1.449873
H	3.265849	-0.003135	0.739047
H	1.661371	-0.025904	2.904231
H	-0.900285	-0.032993	2.034111
H	1.688448	-3.081281	-1.592077
H	3.151088	-3.459180	0.641923
H	-1.033887	-3.182113	1.813470
H	-2.843112	-5.628612	-2.465174
H	-1.688964	-4.527222	-3.225041
H	-1.270882	-5.340623	-1.700407
H	-4.642137	-3.844125	-2.431505
H	-4.312266	-2.247055	-1.729405
H	-3.532828	-2.728093	-3.246140
H	-4.029745	-4.910148	-0.256292
H	-3.607587	-3.355823	0.481488
H	-1.381094	3.694716	-1.509186
H	-2.490647	2.745432	-0.497127
H	-0.741624	2.667469	-0.219884
H	-2.579772	2.630055	-3.504162
H	-2.494222	0.881752	-3.766505
H	-3.536367	1.539216	-2.487407
H	-0.168661	2.700737	-3.375675
H	0.755920	1.903959	-2.106222

5c

E(ωB97XD/6-311+G**)= -7451.218737

C	0.043995	-0.093347	0.074506
C	0.058903	-0.025357	1.491541
C	1.415807	0.049442	1.913329
C	2.239986	0.033231	0.756848
C	1.397573	-0.066129	-0.394622
Fe	0.976401	1.626912	0.683700
C	-0.062991	3.268702	0.002451
C	0.031055	3.308268	1.415659
C	1.408526	3.314452	1.771792
C	2.174567	3.268231	0.578366
C	1.264000	3.245732	-0.526846
P	1.660003	3.180726	-2.293638
Se	-0.060229	2.862961	-3.479434
P	1.763316	-0.134117	-2.183996
Se	3.292535	1.576075	-2.409515
C	2.898887	-1.639680	-2.343308
C	3.279400	-1.746693	-3.827472
C	2.008639	-2.832851	-1.957294
C	4.162833	-1.655652	-1.480846
C	2.576932	4.771985	-2.700709

C	1.610229	5.926152	-2.397879
C	2.931545	4.756454	-4.192026
C	3.847071	4.937196	-1.858245
H	-0.830101	-0.126110	-0.559152
H	-0.807159	0.001617	2.135780
H	1.758631	0.145142	2.932932
H	3.313965	0.134532	0.742129
H	-0.961515	3.230109	-0.594123
H	-0.800634	3.300877	2.104009
H	1.805129	3.309034	2.776041
H	3.249599	3.205220	0.514733
H	4.735534	-2.565121	-1.696237
H	4.815022	-0.801905	-1.683910
H	3.924839	-1.664371	-0.416161
H	3.811847	-2.688097	-4.000689
H	2.395285	-1.734385	-4.470658
H	3.935358	-0.929199	-4.138762
H	2.561496	-3.764508	-2.118571
H	1.717765	-2.791973	-0.904024
H	1.099484	-2.874081	-2.564435
H	4.350350	5.857428	-2.171204
H	3.619410	5.032955	-0.795460
H	4.552798	4.114007	-1.994616
H	3.399983	5.711048	-4.451685
H	3.635234	3.955712	-4.434360
H	2.040588	4.628637	-4.809515
H	2.103946	6.871224	-2.645957
H	0.697430	5.847088	-2.991151

G(ωB97XD/6-311G**)= -7450.852558
E(ωB97XD/6-311+G**)=-7451.203251

C	0.004357	-0.003562	-0.010963
C	0.012319	0.004933	1.417296
C	1.361605	-0.002241	1.846663
C	2.196458	-0.011109	0.695790
C	1.365864	-0.019203	-0.453745
Fe	0.972589	-1.653629	0.684336
C	0.079931	-3.306960	-0.126624
C	-0.002530	-3.340244	1.302726
C	1.313986	-3.319223	1.829067
C	2.222492	-3.267161	0.736553
C	1.467916	-3.250425	-0.465803
P	-1.439490	-3.328969	-1.141991
C	-1.188695	-4.821435	-2.275387
C	0.081129	-4.846702	-3.128689
P	-1.496107	-0.013847	-1.025486
C	-1.498214	1.595807	-1.995298
C	-0.249289	1.740076	-2.872288
Se	-3.231988	-0.311843	0.138428
Se	-1.034862	-1.601179	-2.613413
C	-2.426906	-4.894390	-3.181208

C	-1.181364	-6.028027	-1.321868
C	-1.529684	2.730418	-0.960949
C	-2.761269	1.634121	-2.862722
H	-0.921937	-3.347058	1.869351
H	1.579485	-3.307085	2.875457
H	3.298312	-3.208626	0.808363
H	1.868012	-3.158655	-1.463360
H	-0.872116	-0.012278	2.035352
H	1.697171	-0.031088	2.872264
H	3.275360	-0.050145	0.693668
H	1.698981	-0.078087	-1.478034
H	0.077852	-5.747381	-3.753051
H	0.149630	-3.984353	-3.797189
H	0.980628	-4.879982	-2.512427
H	-2.403251	-5.825505	-3.757315
H	-3.351644	-4.879826	-2.598247
H	-2.461320	-4.062695	-3.890054
H	-1.142477	-6.952502	-1.907613
H	-0.310745	-6.010561	-0.660633
H	-2.083061	-6.061665	-0.703422
H	-0.326599	2.678986	-3.429169
H	0.662511	1.786456	-2.274843
H	-0.152120	0.932475	-3.601975
H	-2.802915	2.597303	-3.380757

4d

E(ωB97XD/6-311+G**)= -3184.240596

P	0.029752	-0.062491	0.043280
C	0.027260	-0.021379	1.940670
C	1.445843	-0.005243	2.522780
C	1.432403	-1.113147	-0.461110
C	2.835006	-0.853123	-0.275457
C	3.556233	-1.769874	-1.081519
C	2.622841	-2.600707	-1.760918
C	1.316755	-2.207236	-1.379563
Fe	2.273102	-0.602608	-2.203111
C	1.502015	1.229806	-2.426087
C	2.904475	1.269046	-2.718497
C	3.152478	0.389569	-3.800682
C	1.916317	-0.192498	-4.195922
C	0.892751	0.320429	-3.359449
P	0.763428	1.933514	-0.914343
Te	2.359555	3.094117	0.407926
C	-0.658994	3.060233	-1.469987
C	-1.615737	3.280905	-0.295919
C	-1.407711	2.494813	-2.682968
C	-0.022421	4.396233	-1.884438
C	-0.769290	1.195278	2.417792
C	-0.662902	-1.313532	2.405319
H	3.259479	-0.059174	0.318950

H	4.630781	-1.806855	-1.179324
H	2.862423	-3.385112	-2.462655
H	0.383281	-2.609130	-1.743779
H	3.633710	1.837785	-2.161453
H	4.119477	0.185104	-4.234543
H	1.777531	-0.921767	-4.979817
H	-0.146230	0.030462	-3.376151
H	-0.811075	5.021487	-2.315132
H	0.417493	4.922362	-1.037398
H	0.752163	4.261370	-2.644778
H	-2.358219	4.030363	-0.586961
H	-2.150949	2.365544	-0.034007
H	-1.088156	3.659117	0.583168
H	-2.235977	3.174210	-2.908136
H	-0.764699	2.453660	-3.564120
H	-1.827630	1.507621	-2.495160
H	-0.574296	-1.365071	3.495229
H	-1.719555	-1.333904	2.139045
H	-0.186189	-2.204642	1.987038
H	-0.873074	1.139557	3.505822
H	-0.258691	2.130823	2.179078
H	-1.773881	1.208080	1.987918
H	1.357217	0.067125	3.611463
H	1.980223	-0.929448	2.295054
H	2.031955	0.845739	2.178572

TS of disproportion of **2b**

G(ωB97XD/6-311G**)=-6091.672431
E(ωB97XD/6-311G**)=-6092.405766

C	-3.032993	1.337310	0.797926
Fe	-3.300408	1.531571	-1.171626
C	-4.424544	1.539397	0.504864
C	-4.319003	-0.423802	3.177766
H	-4.581729	-0.702340	4.204379
S	-4.895042	-2.209921	0.041808
P	-3.068385	-1.555171	-0.329954
C	-1.884956	-2.940796	-0.795738
H	-2.480905	-4.126545	0.938886
C	-1.570571	-3.779664	0.444607
C	-0.580821	-2.406134	-1.397608
C	2.833341	-1.412579	-2.908107
Fe	3.734121	-1.415247	-1.060969
C	2.730949	-2.296681	0.479420
C	3.691461	-3.207990	-0.027564
H	4.902775	-1.867857	-3.617701
C	3.121608	0.960129	2.937064
P	2.525751	0.460517	1.222803

C	2.284165	3.039361	-0.909749
C	2.371788	2.222808	3.375727
H	0.478687	1.824193	-0.863341
C	0.995580	2.507686	-1.539599
P	3.532993	1.726182	-0.358959
C	2.338840	-0.374926	-2.077394
C	3.439553	0.460590	-1.686950
C	4.609638	-0.079664	-2.315936
C	4.236181	-1.232000	-3.055145
C	4.973875	-2.602381	0.072711
C	4.809952	-1.311131	0.639569
C	3.409888	-1.102214	0.883145
C	-4.558147	2.792997	-0.144207
C	-3.267216	3.381014	-0.249851
C	-2.327985	2.496737	0.336512
S	0.150534	0.099888	1.190998
P	-2.180166	-0.200813	1.274953
C	-2.809497	-0.611087	2.999579
C	-2.407798	-2.046308	3.346691
C	1.926404	3.903749	0.302409
C	3.047302	3.881658	-1.944268
C	-1.893085	0.441546	-2.111278
C	-2.369396	1.437578	-3.000981
C	-3.770303	1.254539	-3.163303
C	-4.168862	0.143009	-2.377613
C	-3.009903	-0.369147	-1.714005

TS of the synproportion in case Ch=S

G(ωB97XD/6-311G**)= -6091.674200
E(ωB97XD/6-311G**)= -6092.4025804

C	-2.521409	1.506027	2.072899
C	-2.901820	1.675451	0.701892
C	-4.331637	1.703163	0.662934
C	-4.814791	1.562878	1.989850
C	-3.697961	1.437894	2.858851
P	-1.650020	1.701965	-0.619002
C	-1.877537	3.381138	-1.430010
C	-1.602881	4.409310	-0.321849
Fe	-3.688565	-0.056877	1.446929
C	-2.643744	-1.818439	1.540594
C	-3.117940	-1.582223	0.212653
C	-4.542423	-1.477370	0.278567
C	-4.936229	-1.661209	1.630625
C	-3.762462	-1.868249	2.408783
P	-1.967898	-1.395341	-1.179114
C	-2.448795	-2.692492	-2.431688
C	-1.492536	-2.545631	-3.624060
S	0.287078	-1.452261	-0.565658
P	2.625122	-1.349505	-0.137040
C	3.578995	-2.911301	-0.584589

C	2.981913	-4.080954	0.206033
C	3.313702	-0.066610	-1.244441
C	2.490918	0.801003	-2.031934
C	3.301820	1.852295	-2.531299
C	4.632813	1.644010	-2.078293
C	4.649709	0.461830	-1.291409
Fe	3.364010	1.774899	-0.467094
C	4.124869	2.213306	1.364751
C	3.612956	3.400250	0.779839
C	2.235308	3.192374	0.501884
C	1.891971	1.875421	0.904147
C	3.068006	1.247515	1.440469
P	3.388975	-0.519493	1.822431
C	2.072416	-0.939209	3.121858
C	0.712358	-0.259453	2.938010
C	2.686502	-0.482955	4.454851
C	1.876422	-2.457845	3.136781
C	5.085319	-2.825418	-0.323522
C	3.317070	-3.130810	-2.083062
S	-2.581681	0.434478	-2.105622
C	-3.252683	3.652060	-2.043277
C	-0.786012	3.479906	-2.506903
C	-3.902288	-2.599871	-2.904768
C	-2.209634	-4.042183	-1.736073

TS of disproportion of **2c**

G(ωB97XD/6-311G**)= -10098.457255
E(ωB97XD/6-311G**)=-10099.181059

C	-3.015548	2.393643	-2.421261
C	-4.409075	2.110161	-2.435588
C	-4.601044	0.794737	-1.943944
C	-3.318386	0.254712	-1.611105
C	-2.334372	1.256419	-1.917146
Fe	-3.643301	1.900590	-0.526184
C	-2.493953	2.526298	1.028371
C	-3.046238	1.222499	1.252701
C	-4.476528	1.355639	1.229961
C	-4.785645	2.716316	0.978155
C	-3.565576	3.436653	0.852678
P	-2.038241	-0.293917	1.171386
P	-3.080782	-1.261112	-0.623730
Se	-4.920098	-2.250907	-0.194981
Se	0.357435	0.276718	0.886133
P	2.835539	0.911581	0.844272
C	3.219338	2.205902	2.159110
C	2.398962	3.465900	1.864323
C	3.850752	-0.532236	1.322879
C	3.301684	-1.850422	1.438415
C	4.371953	-2.778381	1.492243
C	5.592440	-2.052204	1.430213

C	5.281107	-0.670054	1.333308
Fe	4.450343	-1.635343	-0.227319
C	3.129296	-1.339492	-1.719903
C	4.106512	-0.287666	-1.663833
C	5.385754	-0.901567	-1.873622
C	5.197618	-2.298846	-2.034237
C	3.804740	-2.567559	-1.940868
P	3.936403	1.438227	-1.060588
C	2.690418	2.197718	-2.266883
C	3.528624	2.526160	-3.513452
C	1.517733	1.301114	-2.667955
C	2.143269	3.493107	-1.660456
C	-2.364594	-1.204496	2.789211
C	-1.827801	-2.633181	2.673961
C	-3.837140	-1.222567	3.209874
C	-1.558248	-0.427412	3.844601
C	-1.897318	-2.350287	-1.608781
C	-2.719035	-2.972130	-2.746945
C	-1.341296	-3.446286	-0.696903
C	-0.743807	-1.542806	-2.211797
C	4.704215	2.568361	2.253135
C	2.750323	1.592403	3.488187

TS of the synproportion in case Ch=Se

G(ωB97XD/6-311G**)= -10098.457255
E(ωB97XD/6-311G**)= -10099.182289

P	3.392519	1.701709	1.060776
C	5.230728	-2.388878	-1.034606
H	5.706440	-0.610117	1.754668
C	3.900006	-2.073198	-0.659274
C	2.318300	-0.904434	2.949309
H	-0.310328	-3.848204	-1.160251
C	1.064903	-0.259028	3.552689
H	3.338293	-0.848404	4.847835
H	0.869727	-0.707159	4.532735
C	-2.495597	-2.248990	1.529584
C	-4.787648	-2.430515	1.508951
C	-3.629650	-2.542501	2.326526
Fe	-3.774196	-0.651371	1.536369
C	-2.769350	1.021023	2.152206
C	-3.388106	1.252468	0.881480
C	-4.792039	1.040212	1.054540
C	-5.028326	0.701621	2.413128
C	-3.777759	0.685163	3.090177
P	-2.360132	1.725248	-0.555583
C	-3.151711	3.322693	-1.175694
H	-4.910243	2.520000	-2.204476
C	-2.872373	4.344485	-0.060776
H	-2.588385	3.053264	-3.275284
C	1.352016	2.843208	-0.670617

C	2.683912	4.313469	0.847616
C	2.001335	2.941935	0.711066
C	0.925109	2.792064	1.789135
C	-0.866636	-2.447337	-3.488005
C	-1.832444	-2.833947	-2.360592
P	-1.836826	-1.420015	-1.126695
C	-2.948176	-1.952157	0.205311
C	-4.373497	-2.064866	0.202457
Se	0.357086	-0.856998	-0.132280
C	4.198853	1.477030	-0.573103
C	5.619496	1.566027	-0.749378
H	6.317263	1.914786	-0.002416
C	5.949695	1.048693	-2.029012
C	4.742177	0.650524	-2.666179
C	3.664631	0.914621	-1.782309
C	6.109272	-1.837343	-0.062299
C	5.324373	-1.172904	0.917132
C	3.941717	-1.301995	0.546726
Fe	4.952616	-0.347293	-0.882271
P	2.489566	-0.370530	1.148218

References

[1] Gaussian 09, Revision B.01, M. J. Frisch, G. W. Trucks, H. B. Schlegel, G. E. Scuseria, M. A. Robb, J. R. Cheeseman, G. Scalmani, V. Barone, B. Mennucci, G. A. Petersson, H. Nakatsuji, M. Caricato, X. Li, H. P. Hratchian, A. F. Izmaylov, J. Bloino, G. Zheng, J. L. Sonnenberg, M. Hada, M. Ehara, K. Toyota, R. Fukuda, J. Hasegawa, M. Ishida, T. Nakajima, Y. Honda, O. Kitao, H. Nakai, T. Vreven, J. A. Montgomery, Jr., J. E. Peralta, F. Ogliaro, M. Bearpark, J. J. Heyd, E. Brothers, K. N. Kudin, V. N. Staroverov, T. Keith, R. Kobayashi, J. Normand, K. Raghavachari, A. Rendell, J. C. Burant, S. S. Iyengar, J. Tomasi, M. Cossi, N. Rega, J. M. Millam, M. Klene, J. E. Knox, J. B. Cross, V. Bakken, C. Adamo, J. Jaramillo, R. Gomperts, R. E. Stratmann, O. Yazyev, A. J. Austin, R. Cammi, C. Pomelli, J. W. Ochterski, R. L. Martin, K. Morokuma, V. G. Zakrzewski, G. A. Voth, P. Salvador, J. J. Dannenberg, S. Dapprich, A. D. , O. Farkas, J. B. Foresman, J. V. Ortiz, J. Cioslowski, D. J. Fox, Gaussian Inc., Wallingford CT, **2010**
[2] G. Schaftenaar, J. H. Noordik, *J. Comput. Aided Mol. Design* 2000, **14**, 123.
[3] W. Humphrey, A.Dalke, K. J. Schulten, *Molec. Graphics*, 1996, **14**, 33-38.
